# Are recent cohorts of women with engineering bachelors less likely to stay in engineering?

**DOI:** 10.3389/fpsyg.2015.01144

**Published:** 2015-08-19

**Authors:** Shulamit Kahn, Donna K. Ginther

**Affiliations:** ^1^Markets, Public Policy and Law Department, Questrom School of Business, Boston UniversityBoston, MA, USA; ^2^Department of Economics and Center for Science, Technology and Economic Policy, University of KansasLawrence, KS, USA

**Keywords:** engineering careers, gender, leaving STEM, women engineers, retention

## Abstract

Women are an increasing percentage of Bachelors in Engineering (BSEs) graduates—rising from 1% in 1970 to 20% in the 2000s—encouraged by increasing K-12 emphasis on attracting girls to STEM and efforts to incorporate engineering and technology into K-12 curricula. Retention of women in STEM and in engineering in particular has been a concern historically. In this paper, we investigate whether this gap has increased because a larger proportion of females entering engineering find themselves ill-matched to this field, or whether the gap has decreased as engineering becomes more accommodating to women. Using 1993–2010 nationally representative NSF SESTAT surveys, we compare cohorts of BSEs at the same early-career stages (from 1–2 to 7–8 years post-bachelors). We find no evidence of a time trend in the gender gap in retention in engineering and a slightly decreasing gender gap in leaving the labor force. We find, as others have, that the majority of the gender retention gap is due to women leaving the labor force entirely and that this exit is highly correlated with child-bearing; yet women with engineering majors are half as likely as all college-educated women to leave the labor market. There are no clear time trends in female BSEs leaving the labor market. Single childless women are actually more likely than men to remain in engineering jobs. Some of the gender differences in retention we find are caused by differences in race and engineering subfield. With controls for these, there is no gender retention difference by 7–8 years post-bachelors for those full-time employed. There were two unusual cohorts—women with 1991–1994 BSEs were particularly likely to remain in engineering and women with 1998–2001 BSEs were particularly likely to leave engineering, compared to men. Cohorts before and after these revert toward the mean, indicating no time trend. Also, women who leave engineering are just as likely as men to stay in math-intensive STEM jobs.

## Introduction

**Figure 1:**
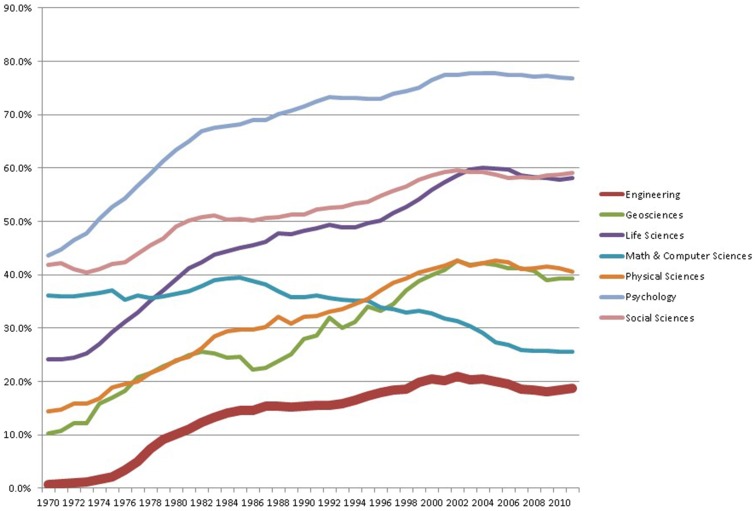
**Percent female among bachelors in engineering compared to other STEM fields over time**. Data Source: NSF WebCASPAR data base (https://ncsesdata.nsf.gov/webcaspar/).

Engineering has been and continues to be a field dominated by men. However, the percentage of women getting bachelors in engineering (BSE) has grown dramatically over the decades, from approximately from 1% in 1970 to 10% in 1980, 15% in 1990 and stabilizing near 20% in the 2000s (NSF WebCASPAR). This has been a period of consciousness-raising about the paucity of women in STEM fields, of rising math test scores among K-12 girls, of more girls taking high school math and science courses, and of women increasing their general college attendance relative to boys (Ceci et al., 2014). Figure 1 illustrates the growth in representation of females among BSE and in other STEM fields.

There has been considerable interest and research on women's retention in STEM in general, and in engineering in particular. Most recently, the National Academy of Engineering and National Research Council (National Academy of Engineering and National Research Council, 2014) convened a workshop on this topic. Women working in the engineering profession, represented by the Society of Women Engineers (SWE), have been very active in surveying women in their field to understand women's greater exit rates. In addition to the Society of Women Engineers (2009) and the National Academy of Engineering and National Research Council (2014) studies, Morgan (2000); Hunt (2010), and Singh et al. (2013) addressed women's exit from engineering particularly, while work such as Preston (1994, 2004), Xie and Shauman (2003), and Xu (2008) addressed exit more generally in all STEM fields.

Previous work on women's retention in engineering was primarily based on cross-sectional data which combines people from many different cohorts (which we identify here by the year of their bachelor's degree in engineering). Measuring retention at different career stages in a cross-section actually combines differences across career stages and differences across cohorts. For instance, in a 2010 cross-section, the only people who would be observed 1 year from their bachelor's degree are the millennials who graduated in 2009, and the only ones who would be observed at 20 years from their bachelors are the Gen X'ers who graduated in 1990.

There are reasons to believe that recent cohorts of engineering majors may behave differently than earlier cohorts did when they were the same age. On the one hand, we might expect later cohorts of women to be more likely to remain in the field because of women's increasing representation among engineering graduates. Hunt (2010) has shown that scientific fields of study with lower female percentages tend to have higher exit rates of women from the field.

On the other hand, we might expect that later cohorts of women engineering majors will be less likely to remain in the field than earlier cohorts. Women might be majoring in engineering in greater numbers because high school curricula have increasingly included engineering and computer education and educators have been encouraged to attract women to engineering (Carr et al., 2012). It may be that some of the women choosing engineering majors today might be less well-matched to the occupation and not find working in engineering satisfying. Therefore, a larger proportion of later cohorts of engineering BSE women may leave engineering after they have spent a few years working in non-engineering fields. In a similar vein, the recent National Academies conference report indicates that excessive workloads, unclear expectations, lack of work-life balance, and a “chilly climate” were associated with women leaving engineering (National Academy of Engineering and National Research Council, 2014). If it is the case that recent cohorts of BSE women are less well-matched to the engineering occupation, these climate issues may increase the propensity of women in more recent cohorts to leave engineering.

These possibilities suggest that we should compare different cohorts of BSE, particularly during the first years after they graduate. We compare whether recent cohorts of women with a BSE leave the engineering field with greater or lesser frequency than previous cohorts. We also test whether there is a general time trend in gender retention differences over the last few decades. Along with this, we might expect that those women who leave engineering might move to non-math-intensive occupations with greater proportions of women.

We test these hypotheses using NSF longitudinal SESTAT data that allow us to study cohorts as recent as 2009 bachelors in engineering (BSE) and as early as those with BSEs in 1985. We use data from eight different waves of the same survey spread over 18 years (1993–2010), allowing us to tease apart differences across cohorts from differences in retention that occur as careers develop, and to further to identify whether the career pattern is different across the cohorts. Moreover, given the panel nature of these surveys, we can follow specific individuals longitudinally for periods as long as 8 years which gives us a better sense of the timing of exit.

## Previous research

Preston (1994, 2004), Xie and Shauman (2003), Xu (2008), and Glass et al. (2013) have studied women's exit from science and engineering as a whole using a variety of national data sources. Preston found large differences in the 1980s and early 1990s. Using data from the 70s, 80s, and early 90s, Xie and Shauman found that women with bachelors in STEM (excluding social sciences) are about one quarter less likely than men to work in STEM occupations and that married women with children are the most affected. Xu (2008), using the 1999 National Survey of Postsecondary Faculty, found that women and men were equally likely to seek to leave STEM academic careers but that women had greater intentions to seek another position within academia. Glass et al. (2013) followed female college graduates from the National Longitudinal Survey of Youth 1979 and found that women in STEM occupations were more likely to leave their field early in their career compared with women in other professional occupations. They find that women in STEM occupations move to non-STEM occupations at very high rates and attribute women's departure from STEM careers to climate issues or job matching.

Research on gender differences in retention in engineering specifically are most germane to this paper. The Society of Women Engineers (2009) surveyed engineering alumni of 21 colleges from 1985 and later. In their 2005 cross section of graduates from these 21 schools whose BSE was their highest degree, there was an average 10% gender gap in the likelihood of working in engineering. Further, they found that 90% of this gender gap was a result of women leaving the labor force entirely. These gender differences were similar to those from the more nationally representative 2003 NSF SESTAT, although overall their retention rates were higher than those in SESTAT.

Morgan (2000) used the 1993 National Survey of College Graduates (NSCG) and captured employment of those who received BSEs between 1965 and 1989 but measured the gap only for those with highest degrees in engineering (i.e., only those who did not choose immediately post-bachelors to enter into a different field via a degree). As such, her estimate of exit is likely to be lower than ours. She found a 3 percentage point (*ppt*.) gender gap in the likelihood that full-time workers with highest degrees in engineering were employed in engineering jobs, defined using a survey question asking whether respondents were working in a field closely or somewhat related to their field of highest degree. In contrast, women in other fields were 6 *ppt*. more likely than men to remain in the field of their highest degree. She also found these women were 9 *ppt*. more likely than men to be out of the labor force and 7 *ppt*. more likely to be working part-time.

Hunt (2010) also uses the NSCG, but from both the 1993 and 2003 surveys. Like Morgan, she studied those with *highest degrees* in engineering and based her analysis on the question of how closely their job related to the field of highest degree. Hunt found about a 10% average gender difference in overall retention^1^, of which 70% could be accounted for by women leaving the labor force (similar to Morgan's 3% gender gap among full-time workers). Also like Morgan (2000), Hunt found that the gender differences in engineering were slightly larger than gender differences in other sciences or in non-STEM fields. Unlike Morgan (2000) and Society of Women Engineers (2009), Hunt estimated gender differences with regression models allowing her to control for field, age, degree level, and race among other factors. Holding these constant, women who studied engineering were slightly more likely than women in other fields to be working (about 1 *ppt*.) but considerably less likely than women in other fields to have a job related to her highest degree (on the order of 5 *ppt*. of those working or about 4 *ppt*. of those irrespective of whether they worked). Finally, Hunt finds that including the male share of the field in the regression model that estimates female exit more-than-explains the lower female retention of women in engineering compared to other non-STEM fields.

The only research using longitudinal data to examine retention in engineering was Greenfield's presentation in National Academy of Engineering and National Research Council (2014), which used data from the Department of Education's Baccalaureate and Beyond. She primarily analyzed the 1992–1993 BSE cohort whose sample was small (560, with 80 women). She measured retention as working in an engineering or architecture job. She found that retention rates for employed women engineering BSEs were *higher than* those of men's in engineering—13.7 *ppt*. higher after 1 year, 14.8 *ppt*. higher than men after 4 years, and 6.8 *ppt*. higher after 10 years. The retention rate of women in engineering was not lower than other fields at 4 years, but was substantially lower at 10 years. She also looked at 1-year retention rates across several later cohorts and found that later cohorts of women were less likely to stay in engineering immediately after receiving their bachelors.

A final relevant finding in Hunt (2010) is that the share of men in the specific sub-field of STEM study was positively highly correlated with women's exit from science (*r* = 0.51) She finds that including the male share of the field in a regression of female exit more-than-explains the lower female retention of women in engineering compared to other non-STEM fields.

Thus, all of these studies find gender differences in retention in engineering that are small relative to the percentages who stay in engineering, contradicting the general impression of much higher exit rates from engineering (e.g., see Singh et al., 2013). One (National Academy of Engineering and National Research Council, 2014) even found women more likely to remain in engineering.

## Materials and methods

SESTAT is collected by the National Science Foundation (NSF) and is the most comprehensive database on the employment, educational, and demographic characteristics of U.S. scientists and engineers available. SESTAT actually includes observations from three NSF surveys: the National Survey of Recent College Graduates (NSRCG), the National Survey of College Graduates (NSCG) and the Survey of Doctorate Recipients (SDR). From the NSCG respondents, SESTAT includes only those who received a degree in STEM or had ever worked in a STEM occupation. From the NSRCG, SESTAT includes recent bachelor's and master's degree recipients in STEM fields. The SDR samples US-awarded PhDs in STEM disciplines. SESTAT oversamples women and under-represented minorities (URMs) in order to allow more accurate measures of gender and racial differences.

Within each decade, SESTAT followed individuals through the different waves, adding new people to represent more recent graduates (from the NSRCG). The 1990s panel includes 4 waves: 1993, 1995, 1997, and 1999. The 2000s panel includes 4 waves: 2003, 2006, 2008, and 2010. SESTAT thus includes as many as four observations on a single individual over a 7 or 8 year span in each decade (although for various reasons many people are seen for fewer than four surveys^2^). Note that there are primarily 2-year gaps between survey waves, although there is one 4-year and one 3-year gap.

SESTAT collects information on education, employment including labor force status, occupation, employer characteristics, work activities, and comprehensive demographic information on gender, race/ethnicity, marital status, children, citizenship, and immigration status.

The measurement of who is working as an engineer is not straightforward. In the majority of this analysis, we define people as working in engineering if their primary occupation is categorized by the NSF as engineering. This excludes all jobs categorized as computer and information scientists, such as computer system analysts. Moreover, it excludes jobs categorized as being engineering-*related*, such as “electrical, electronic, industrial, and mechanical technologists and technicians” or architects. Based on the 2010 SESTAT, we calculate that 1.73 million people were employed full-time in engineering jobs, 2.31 million in computer jobs, and 0.46 million in engineering-related jobs. Beginning in 2003, SESTAT began including low to mid-level “engineering managers” within engineering occupations, but not “top level managers, executives, and administrators.” “Engineering managers” (or manageers, a term we have coined) represented 15.6% of the 1.73 million full-time engineering jobs in 2010. Because we want to compare cohorts working in the 1990s as well as the 2000s, we exclude engineering managers in our analysis of engineering retention across cohorts. That said, we also analyze whether BSEs moved into management jobs and if so, whether the job was required technical STEM education.

We use the SESTAT data to examine gender differences in remaining in engineering by cohort and years since degree. Our cohort analysis is based on the 28,117 individuals in SESTAT surveyed who received their first bachelor's degree in engineering (BSE)^3^ between 1985 and 2009. For ease of presentation, we divide cohorts into approximately 3- to 5-year BSE groupings starting with the 1985–1990 cohort and ending with the 2006–2009 cohort, choosing endpoints so each cohort has enough observations to create reasonably accurate statistics. Individuals in the analysis were observed in a SESTAT survey at either 1–2 years, 3–4 years, and/or 7–8 years post-BSE. We also examine outcomes for people working 15–16 years after the degree, but the number of women in this older cohort is small.

We begin our cohort analysis using descriptive statistics to examine gender differences in remaining in engineering by years since PhD for the outcomes of (1) being “engaged in engineering,” defined as working in an engineering occupation or enrolled in an advanced engineering degree program^4^; (2) working full-time in an engineering occupation for the subsample that is employed 35 or more hours per week; and (3) being out of the labor force—defined as not working and not looking for work. We then use linear probability regressions to estimate gender differences in these same outcomes, controlling for things that might be responsible for gender differences but that are not directly attributable to gender *per se*, including engineering subfield, survey year, immigrant status, race, and one measure of socioeconomic class, whether the parent had graduated college. We present the coefficient on gender from these models in order to examine differences in remaining in engineering across cohorts. We then take a closer look at factors associated with leaving the labor force by adding interaction terms to our linear probability models, specifically interaction terms for female X cohort X family-status. Finally, for those who leave engineering, we examine where they go—to engineering related, other mathematically intensive STEM, non-mathematical STEM, or non-STEM occupations.

Stata 13 was used for all statistical analysis including the linear probability multiple regression models. The paper only includes those results related to gender differences. Full regression results for all regression tables are available in the Supplementary Material.

## Results

### Average gender differences in retention post-bachelors

#### 2010 Averages

**Figure 2:**
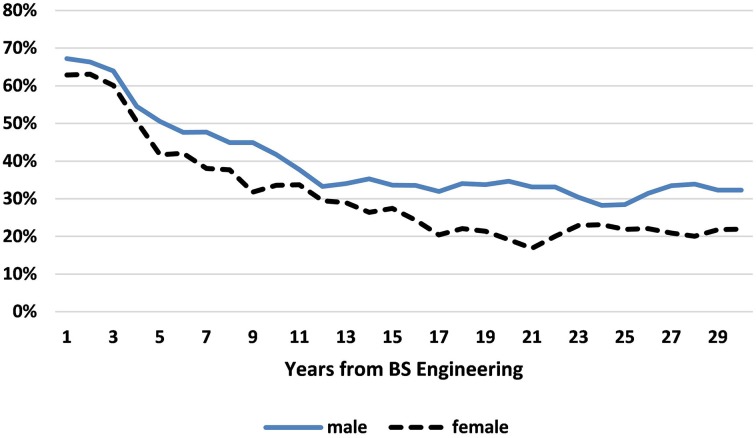
**Percent of female and male Bachelors of Engineering (BSEs) remaining in engineering, by years from BSE (3-year moving averages)**. Data Source: NSF SESTAT Survey 2010.

Figure 2 shows the proportion of women and men, respectively with BSEs who in 2010 are “engaged in engineering” graphed by years since the BSE. We use 3-year moving averages because of the erratic periodicity of SESTAT surveys and the small number of females at each point. Figure 2 demonstrates the starting point of this paper, that in the 2010 cross-sectional data, after a few years post-BSE a gap appears and women with BSEs become less likely to be working in engineering jobs than men. The average gender difference in remaining in engineering (for those within 30 years of the BSE) is 7.8 percentage points (or *ppt*.) At 10 years post-bachelors, the gender difference is 8.2 *ppt*.; at 20 years, it is 15.5 *ppt*. and at 30 years, it is 10.4 *ppt*. We note, however, that the sample size of women engineers who in 2010 were more than 18 years post-BSE is very small (<100 individuals per year), so the right-hand side of the graph must be considered only suggestive.

Some of the gender difference in engineering retention may simply be due to the fact that more women than men are not working at all (either unemployed or out of the labor force) or working part-time. Among those in the 2010 SESTAT within 30 years of their BSE, 19.2% of women but only 5.6% of men were not working, a difference of 13.6 *ppt*. The 2010 percentage of women not working among BSEs is similar to the 20.0% not working in 2010 among all US women with a bachelors or higher^5^.

Moreover, rather than leave the labor force, some people instead choose to work part-time. In 2010, 5.7% of those with BSEs in engineering (within the past 30 years) worked part-time. There is a large gender difference in the likelihood of working part time (as would be expected if women are the primary child-caregivers): 12.7% of women with BSEs but only 4.1% of men were working part-time.

Two facts suggest that there are fewer part-time jobs available within engineering than are desired by BSEs. First, 32.4% of women with BSEs who worked part-time were in engineering jobs compared to 38.5% of women with BSEs who worked full-time. Second, only 5.7% of all those with a BSE work part-time, much less than the 14.4% working part-time of those with non-engineering STEM bachelors. This suggests that if a person with a BSE wants to work part-time, she/he is much more likely to be forced to work outside of engineering. This paucity of part-time jobs within engineering may be due to choices made by employers insensitive to women's flexibility needs, a point we discuss in the conclusion.

Including only those BSE's working full-time eliminates 32.4% of female BSEs compared to 10.3% of male BSEs. The average gender difference in remaining in engineering among full-time-working BSEs (2010, first 30 years) is 1.6 *ppt*., much less than the *7.8 ppt*. average for the entire population.

**Figure 3:**
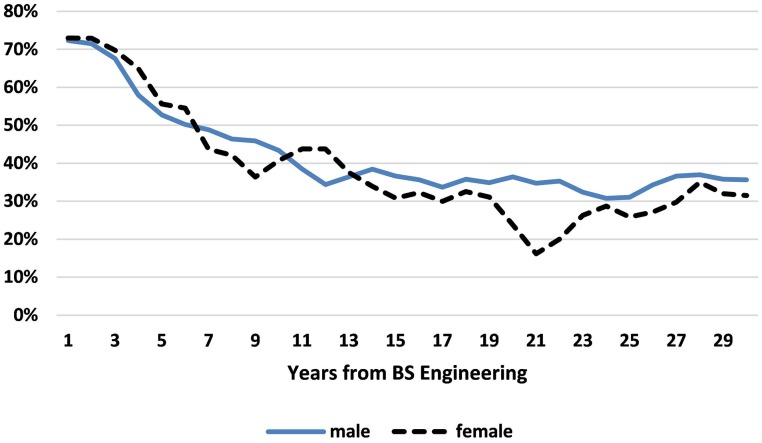
**Percent of female and male Bachelors of Engineering (BSEs) working full time who remain in engineering, by years from BSE**. Data Source: NSF SESTAT Surveys 1993–2010.

Figure 3 includes only those BSEs who are working *full-time* and graphs the percent in engineering for men and women separately. We see that in the 15 years after their undergraduate diploma, on average men and women are *equally likely* to remain in engineering, with periods when women are more likely than men to do so. Beyond 15 years post-BSE, however, men are consistently more likely to remain in engineering, with the gap fluctuating considerably due to even smaller sample sizes of full-time working women than in Figure 2.

#### 1993–2010 averages

As noted earlier, using a single SESTAT year (2010) confuses cohort and career stage differences. Instead, we use the data from all 8 SESTAT waves from 1993 to 2010 to measure the gender retention gap at three different early career stages (measured by years from BSE): 1–2 years after their bachelors, 3–4 years after their bachelors, and 7–8 years after their bachelors. We use 2-year career-stage spans because in most cases, SESTAT surveys were administered every 2 years (We also do limited analyses for the stage 9–16 years post-BSE).

Table 1 gives the average probability that men and women remain in engineering (either working or getting higher degrees) at the three different career stages averaging over individuals in the sample observed at this career stage. Before we discuss cohort-specific gender retention, we first describe this average retention at each career stage using both descriptive statistics (Table 1) and regression analysis (Table 2).

**Table 1:** **Average probability of remaining in engineering (working or studying) or out of the labor force: all cohorts combined**.

	**% of all BSE grads engaged in engineering**			**% of BSE grads working FT in engineering**			**% Out of the Labor Force**			**# Observations**	
	**Male (%)**	**Female (%)**	**Female-male difference (%)**	**Male (%)**	**Female (%)**	**Female-male difference (%)**	**Male (%)**	**Female (%)**	**Female-male difference (%)**	**Male**	**Female**
1–2 years post-BSE	61.38	60.54	−0.84	67.75	70.82	3.07^***^	5.55	8.41	2.86^***^	12162	4695
3–4 years post-BSE	61.35	57.79	−3.56^***^	65.95	66.45	0.50	4.39	6.76	2.37^***^	10733	3773
7–8 years post-BSE	53.58	45.33	−8.26^***^	56.04	53.00	−3.04^**^	1.77	10.30	8.53^***^	9205	2607

Gender difference t-test

*** *p < 0.01*,

** *p < 0.05*.

*15–16 averages cannot be given because the #observations in some cases are too small to report*.

**Table 2:** **Coefficient on female from linear probability models of remaining in engineering: all cohorts combined**.

	**Probability of remaining in engineering**		**Probability of leaving the labor force**
	**Population: all**	**Population: working FT**	**Population: all**
1–2 years post-BSE	0.0127	0.0362^***^	0.0073
	(0.0094)	(0.0100)	(0.0048)
3–4 years post-BSE	−0.0163	0.0185^*^	0.0102^**^
	(0.0102)	(0.0108)	(0.0047)
7–8 years post-BSE	−0.0620^***^	−0.0092	0.0834^***^
	(0.0119)	(0.0131)	(0.0044)
15–16 Years post-BSE if still in Eng at 7–9 years	−0.0072	0.0905^*^	0.1053^***^
	(0.0474)	(0.0508)	(0.0159)

Coefficient significance

*** *p < 0.01*,

** *p < 0.05*,

* *p < 0.1*.

*Standard errors in parentheses*.

*Controls include dummies for engineering subfield, survey year, BSE year, if parent had ≥BA/BS, immigrant status, race*.

*#obs: All population: 1–2 years: 16,857; 3–4 years: 14,506; 7–8 years: 11,812; 15–16 years: 884*.

*#obs: FT only: 1–2 years: 13,382; 3–4 years: 12,501; 7–8 years: 10,585; 15–16 years: 848*.

The first row of Table 1 tells us that 61% of both male and female BSEs enter an engineering job (or schooling) in the 1–2 years immediately after graduating with a BSE, 39% do not. There is no (significant) gender difference. By 3–4 years post-BSE, a gender difference had appeared, where women were 3.6 percentage points (*ppt.)* less likely than men to remain in engineering; and by 7–8 years, this gender difference had widened to 8.3 *ppt*. Columns 4 through 6 include only those working full time. Since women are more likely than men to leave the labor force as well as more likely to work part-time, excluding these two groups from the population (as well as the unemployed^6^) changes the gender difference considerably at all career stages. At 1–2 years, those women working full-time were significantly *more* likely than men (3.1 *ppt*.) to remain in engineering on average; at 3–4 years men and women were insignificantly different; and only by 7–8 years were women less likely to remain in engineering, with a significant gender difference of 3.0 *ppt*.

The last three columns confirm that at each career stage, on average females are more likely than men to be out of the labor force completely, but that the main movement out of the labor force occurs between 4 and 8 years of the BSE.

#### Regression analyses of average retention

Table 2 uses linear probability regressions to calculate these same measures at the same three career stages, controlling for engineering subfield, survey year, immigrant status, race, and one measure of socioeconomic class, whether the parent had graduated college.

We highlight only those Table 2 results that are qualitatively different from what was found in the simple descriptive statistics of Table 1. Compared to Table 1, at 3–4 years post-BSE, the addition of controls erased the gender difference for the population as a whole (Neither table finds a gender difference retention disadvantage for full-time workers at this stage). At 7–8 years, for the whole population, what was an 8.3 *ppt*. gender difference in Table 1 becomes 6.2 *ppt*. with controls (Table 2); in contrast, among those working full time, there is no longer a significant gender difference. Finally, with controls, gender differences in being *out of the labor force* (Table 2) are somewhat smaller than without controls (Table 1) and no longer significant at 1–2 years. Overall, then, the control variables do explain some of the gender differences observed in the descriptive statistics. In work not shown, we investigated which of the controls variables were the major mediating factors. We found that subfield was one important factor but that race/ethnicity was the most important control variable responsible for some of the average gender gap^7^. Women in engineering are less likely than men to be white (non-Hispanics)—the race with the highest retention rates—and more likely to be Asian or black, both groups with lower retention rates. This result suggests that racial retention rates are important to study in future research.

The last row models retention at an even later career stages by asking, “Of those who remain working in engineering 7–8 after their degree, what is the gender difference in the likelihood of remaining in engineering approximately 8 years later?” ^8^ This allows us to incorporate BSEs as early as 1984, even though the earliest BSEs we can observe at their careers' beginning are from 1991^9^. This row indicates that there was no significant gender retention difference during years 8–16 among those people who were still in engineering at the beginning of this stage. When we look only at those who are still full-time employed at year 15–16 post-BSE, on average women are *more* likely than men to remain in engineering.

### Differences across cohorts

Tables 3,4 present gender differences for cohorts defined by narrow ranges of BSE years. Table 3 gives averages per cohort/gender, while each panel of Table 4 gives coefficients from a linear probability regression run with interaction terms between the female dummy variables and a dummy variable for each cohort, as well as on other control variables.

**Table 3:** **Average probability of remaining in engineering (working or studying) or out of the labor force by BSE year cohort**.

**Cohort (BSE years)**	**% of all BSE grads engaged in engineering**			**% of BSE grads working FT in engineering**			**% Out of the Labor Force**			**# Observations**	
	**Male (%)**	**Female (%)**	**Female-male difference (%)**	**Male (%)**	**Female (%)**	**Female-male difference (%)**	**Male (%)**	**Female (%)**	**Female-male difference (%)**	**Male**	**Female**
**(A) 1–2 YEARS POST-BSE**											
1991–1994	57.34	65.94	8.59^***^	63.99	73.64	9.65^***^	4.20	4.28	0.07	4601	1077
1995–1997	62.89	60.48	−2.41	68.47	68.77	0.31	4.53	6.45	1.92^*^	2237	663
1998–2001	62.04	57.08	−4.96^*^	69.59	69.95	0.35	5.18	11.79	6.61^***^	1362	546
2002–2005	59.47	55.95	−3.51	66.87	66.46	−0.42	6.79	9.78	2.98^**^	1957	886
2006–2009	64.86	62.06	−2.80	70.45	74.18	3.73	6.82	10.06	3.24^***^	2005	1523
**(B) 3–4 YEARS POST-BSE**											
1989–1990	62.04	58.22	−3.82	68.44	68.75	0.31	4.74	7.66	2.92^***^	2526	561
1991–1994	61.94	67.55	5.61^**^	66.76	75.38	8.62^***^	4.65	5.18	0.52	2575	598
1995–1997	60.20	57.31	−2.89	63.78	62.26	−1.52	3.66	3.94	0.27	1104	328
1998–2001	60.39	53.18	−7.21^**^	64.86	63.34	−1.52	2.75	6.08	3.34^***^	933	366
2002–2005	61.51	53.45	−8.05^***^	66.65	62.04	−4.61^**^	5.07	8.93	3.85^***^	2510	1336
2006–2007	61.13	57.91	−3.22	64.12	68.10	3.97	4.36	6.77	2.42^*^	1085	584
**(C) 7–8 YEARS POST-BSE**											
1985–1990	56.75	49.14	−7.61^***^	59.13	58.14	−0.99	1.69	12.40	10.71^***^	4607	957
1991–1994	54.56	57.90	3.33	56.64	65.84	9.20^**^	1.88	9.94	8.06^***^	996	253
1995–1997	49.66	42.52	−7.15^*^	52.97	55.38	2.42	1.81	11.90	10.09^***^	919	234
1998–2001	56.20	43.19	−13.01^***^	59.44	49.25	−10.19^***^	1.84	8.92	7.07^***^	1763	789
2002–2003	44.93	38.80	−6.13^*^	45.57	43.67	−1.90	1.74	8.14	6.40^***^	920	374

Gender difference t-test

*** *p < 0.01*,

** *p < 0.05*,

* *p < 0.1*.

**Table 4:** **Gender differences in remaining in engineering or leaving the labor force by cohort (calculated as the coefficient on female−cohort interaction from a linear probability regression at each stage)**.

**Cohort (BSE years)**	**Probability of Remaining in Engineering**		**Probability of Leaving the Labor Force**
	**Population: All**	**Population: Working FT**	**Population: All**
**(A) 1–2 YEARS POST-BSE**			
1991–1994	0.1049^***^	0.1140^***^	−0.0102
	(0.0203)	(0.0210)	(0.0103)
1995–1997	−0.0074	0.0026	0.0036
	(0.0206)	(0.0213)	(0.0105)
1998–2001	−0.0303	0.0143	0.0414^***^
	(0.0242)	(0.0260)	(0.0123)
2002–2005	−0.0191	−0.0097	0.0125
	(0.0198)	(0.0214)	(0.0101)
2006–2009	0.0012	0.0430^**^	0.0032
	(0.0178)	(0.0194)	(0.0090)
**(B) 3–4 YEARS POST-BSE**			
1989–1990	−0.0327	0.0074	0.0249^*^
	(0.0308)	(0.0334)	(0.0140)
1991–1994	0.0717^***^	0.1014^***^	−0.0017
	(0.0222)	(0.0233)	(0.0101)
1995–1997	−0.0184	−0.0044	−0.0039
	(0.0292)	(0.0303)	(0.0133)
1998–2001	−0.0570^**^	−0.0066	0.0230^*^
	(0.0277)	(0.0295)	(0.0126)
2002–2005	−0.0552^***^	−0.0299	0.0183^**^
	(0.0188)	(0.0198)	(0.0086)
2006–2007	−0.0135	0.0434	0.0012
	(0.0252)	(0.0272)	(0.0115)
**(C) 7–8 YEARS POST-BSE**			
1985–1990	−0.0574^***^	0.0141	0.1057^***^
	(0.0206)	(0.0231)	(0.0075)
1991–1994	0.0696^*^	0.1213^***^	0.0781^***^
	(0.0396)	(0.0429)	(0.0145)
1995–1997	−0.0682^**^	0.0226	0.0987^***^
	(0.0323)	(0.0369)	(0.0118)
1998–2001	−0.1201^***^	−0.0926^***^	0.0695^***^
	(0.0221)	(0.0241)	(0.0081)
2002–2003	−0.0390	−0.0035	0.0568^***^
	(0.0275)	(0.0299)	(0.0101)
**(D) FROM 9–16 YEARS POST-BSE IF STILL IN ENGINEERING AT 7–9 YEARS**			
1984	−0.1313^**^	0.0310	0.1833^***^
	(0.0654)	(0.0731)	(0.0218)
1987–1994	−0.0623	−0.0058	0.0521
	(0.0978)	(0.0997)	(0.0326)
1995	0.3289^**^	0.2708^**^	−0.0504
	(0.1287)	(0.1324)	(0.0429)

*Controls include dummies for engineering subfield, survey year, BE year, if parent had ≥BA/BS, immigrant status, race*.

*Because of the irregular SESTAT periodicity, the following intermediate BE years are not in the data*.

*(A) 1999, 2000, 2003; (B) 1997, 1998, 2001; (C) 1993, 1994, 1997; (D) 1989, 1993*.

*#obs: All population: (A) 16,857; (B) 14,506; (C) 11,812; (D) 884*.

*#obs: FT only: (A) 13,382; (B) 12,501; (C) 10,585; (D) 848*.

We cannot compare exactly the same cohorts across all career stages, for two reasons. First, the latest BSE years are only observed in their first career stages, while the earliest BSE years are only seen in their later career stages. Second, we lose some BSE years when SESTAT did not have the standard 2-year periodicity^10^. Specifically, we do not observe those with BSEs in 1999, 2000, or 2003 at the 1–2 year mark, we do not observe those with BSEs in 1997, 1998, and 2001 at the 3–4 year mark, and we do not observe those with BSE's in 1993, 1994, and 1997 at the 7–8 year mark. In the analysis of the 8 to 16 year career stage, we have information about even fewer cohorts since the cohorts need to be observed both at the 7–9 year point (to see if they start the stage in engineering) and again at the 15–16 year point, meaning the last observed cohort have 1995 BSEs.

**Figure 4:**
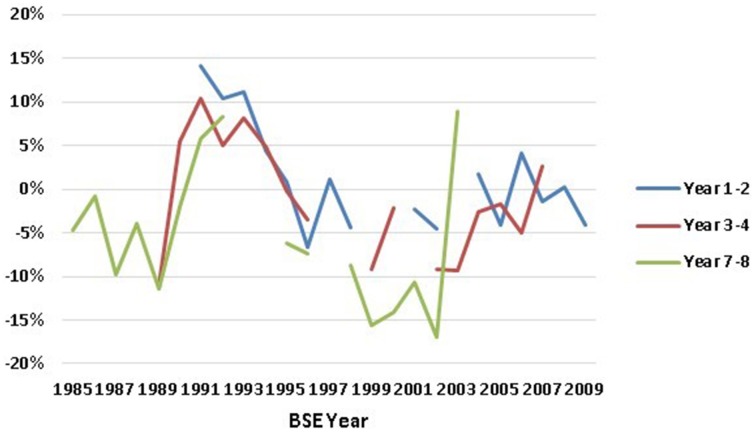
**Gender gap in retention in engineering, by BSE-year, calculated as coefficients on Female X BSE-year interaction terms in regression results of Table A1 in Supplementary Material**. Data Source: NSF SESTAT Surveys 1993–2010.

In addition, we have estimated linear probability models with single-year cohorts (Table A1 in Supplementary Material). Since each annual cohort sample is small, the majority of single-year-cohort gender gaps are not significantly different from zero. Nevertheless, this analysis does help us to analyze whether our arbitrary cohort definitions hid large variation within multi-year cohorts. The Supplementary Table A1 gender gap coefficients for the whole population are graphed as Figure 4. Our discussion below will primarily be based on the multi-year cohorts of Tables 3, 4; however, we refer to Table A1 in Supplementary Material analysis when results on gender differences in single years adds to our understanding.

#### Cohort differences at 1–2 years

In our earlier discussion of the averages across all cohorts, we found no differences in the retention of women and men in engineering in the first 2 years post-BSE receipt, with or without controls. There was a significant but modest difference in women leaving the labor force that seemed to be due to race and subfields. Among who were working full time, however, women were actually significantly more likely to remain in engineering than men at this stage (with and without controls).

This same pattern is not shared by all cohorts. For four out of the five cohorts—all those with 1995 to 2009 BSEs—the estimated average differences (Table 3 first columns) suggest that women were less likely than men to remain in engineering at this early career stage. While this difference was only significant for one cohort (those with BSEs 1998–2001), if we combined the four cohorts 1995–2009, the overall gender difference is highly significant (*p* = 0.001). Adding controls (Table 4 first column) lowers numerical estimates of the gender difference for these 4 cohorts. Moreover, not only are none of the gender differences in these four cohorts significant in Table 4 (not even 1998–2001), but the combined 1995–2006 effect is small and insignificant as well. The year-by-year results in the Supplementary Material Table A1 (graphed in Figure 4) show only a single year—2006—with a significant and negative gender difference at the 1–2 year stage between 1995 and 2009.

Returning to Table 3, the four cohorts (1995–2009) where women were less or equally likely to remain in engineering in the 2 years post-BSE are balanced by a single cohort where women are much *more* likely to remain, leading to a zero average gender difference. Women in the 1991–1994 cohort were 8.6 *ppt*. *more likely* than men to remain in engineering; adding controls (Table 4) increases the gender difference to a positive 10.5 *ppt*. (Table A1 in Supplementary Material demonstrates that significantly higher women's retention was observed for 1991, 1992, and 1993 BSEs). Comparing the 1991–1994 cohort to the one immediately after, Table 3 suggests that both a higher engagement of women in engineering and a lower engagement of men contributed to the gender difference.

Gender differences in leaving the labor force were significant for all four cohorts, although smaller in Table 4 with controls and not significant except for the 1998–2001 cohort. The more noisy year-by-year analysis of Table A1 in Supplementary Material indicates 4 years with significantly higher female labor force exit (1996, 1998, 2001, 2007) and 2 years with significantly lower female labor force exit (1995, 2009), scattered throughout the period.

Limiting the analysis to those who worked full-time, there were *no cohorts where women were significantly less likely than men to remain* within 2 years of their BSEs in either Table 3 or Table 4. Full-time working BSE women in the cohort of 1991–1994 were much more likely to remain in engineering than men, with full-time women 9.6 *ppt*. more likely to remain without controls and 11.4 *ppt*. more likely with them^11^. In addition, the most recent cohort of full-time working women, those who received their BSEs in 2006–2009, were also more likely than comparable men to remain in engineering in years 1–2, with the difference more significant with controls (*p* = 0.027) than without (*p* = 0.106). In the year-by-year analysis, this is reflected in positive coefficients in 2006–2008, significant (and large) in 2006.

#### Cohort differences at 3–4 years

In the averages discussed earlier, women were less likely than men to remain in engineering at 3–4 years post-BSE, although this was mostly explained by controls. Women were also more likely to leave the labor force. Limiting to those working full time, not only did the average gender difference in retention disappear, but with controls it seemed that FT working women were 1.8 *ppt*. *more* likely than men to stay in engineering at this career point.

When we divide this into cohorts, we find that this pattern was generally accurate for five of the six cohorts observed at this stage, with the exception again being those with BSEs 1991–1994. Thus, for each of the other five cohorts, women were less likely to remain in engineering than men at the 3–4 year point; these differences were significant for only two of the five cohorts: 1998–2001 and 2002–2005. This was true without (Table 3) or with (Table 4) controls. The year-by-year effects (Table A1 in Supplementary Material) corroborate these results.

In terms of exit from the labor force, significant gender differences are present for these two cohorts as well as for the earliest cohort (BSEs 1989–1990). As a consequence, limiting the analysis to full-time workers shrinks the gender retention differences for these 5 cohorts: without controls only the average gender gap for the 2002–2005 full-time cohort remained significantly negative; with controls, *none* of these five cohorts had significantly lower full-time female retention rates.

As we saw at 1–2 years, the exceptional cohort at 3–4 years was those with BSEs in 1991–1994. These women were 5.6 *ppt*. more likely to remain in engineering than men on average (Table 3), 7.2 *ppt*. more likely with controls (Table 4). Full-time working women were 10.1 *ppt*. more likely than full-time men to remain in engineering with controls, and there was no gender difference in exit from the labor force. The year-by-year results of Table A1 corroborate this unusual pattern for each year of this cohort, including 1990. Men's participation in engineering at this stage was not particularly low for this cohort; instead, women's participation was particularly high.

Based only on the 1–2 year career stage, we might have concluded that women in later cohorts were more likely than men to leave engineering, because the earliest cohort observed (1991–1994 BSEs) were so different than those after it. At the 3–4 year career stage, we can now observe earlier cohorts than 1991–1994 BSEs. We see that 1991–1994 BSE was *not* representative of earlier cohorts. Instead, it was only the 1991–1994 cohort that was exceptional in its staying power.

#### Cohort differences at 7–8 years

Seven to eight years post-BSE, averaging across cohorts women were less likely to remain in engineering with or without controls, with larger differences (8.3 *ppt*.) than seen at earlier stages. This had been primarily due to 8.5% more women than men leaving the full time labor force. Among those who worked full-time, the average gender difference in retention dropped to 3.0 *ppt*. and with controls became less than 1 *ppt*. and insignificant.

Again, the cohort analysis indicates that a higher retention of women compared to men in the 1991–1994 cohort had been balancing out negative gender differences among the other cohorts. Women from all other cohorts (1985–1990, 1995–2003) were significantly less likely than men to remain in engineering by year 7–8, with gender differences in cohorts ranging from 6.1 *ppt*. to 13.0 *ppt*. (Table 3). Adding controls (Table 4) makes these gender differences only modestly smaller and still significant, with the exception of the 2002–2003 cohort—the latest one—whose significance falls to *p* = 0.15.

Women were much more likely than men to have left the labor force at year 7–8 across *all* cohorts including the 1991–1994 cohort and the 1995–1997 cohort (with 8.1 *ppt*. and 10.1 *ppt*. gender differences), two cohorts that previously had not left in greater numbers than men.

Despite this, women in the 1991–1994 cohort who remained working full-time continued to be *much more likely* than men in this cohort to remain in engineering with and without controls (9.2 *ppt*. and 12.1 *ppt*., respectively), and also much more likely to remain in engineering than women in the previous or subsequent cohorts.

Only women in the 1998–2001 cohort continued to have a significant and large gender disadvantage in retention among those working full-time, 10.2 *ppt*. without controls and 9.3 *ppt*. with. This gender difference was equally due to men's high likelihood of remaining in engineering and women's low likelihood of remaining.

The year-by-year effects from Table A1 in the Supplementary Material and Figure 4 add interesting nuances. Every one of the separate year effects 1998–2002 showed significantly lower female retention for both the whole and the full-time sample, and significantly higher female rates of leaving the labor force. Among other things, this suggests that the cohort should have been defined as 1998–2002. BSEs from 1991 and 1992 (the only years between 1991 and1994 observed by SESTAT at the 7–8 year point^12^) had significantly positive gender differences for full-time women.

#### Cohort differences at later career stages

We only observe a limited number of BSE years at later career stages. The cohort analysis of Table 4 Panel D follows those who were observed working in engineering at approximately 7–9 years post-BSE through year 15–16. It includes only 884 observations, 152 of whom were female. The earliest observable cohort year of 1984 had large gender-differences (13.1 *ppt*.) in engineering retention by the 15th–16th year. This was due to an extremely high rate of women's leaving the labor force: no gender difference remained among those working full-time. Those with 1995 BSEs who had remained working in engineering through year 7–9 were *more* likely than men to remain in engineering at year 15–16 and equally likely as men to remain in the labor force. Given the SESTAT timing, we observe few people who received BSEs between 1985 and 1994 so results completely lacked power and significance. Because Panel D analysis is based on so few observations, we consider these results only suggestive.

#### Estimating cohort gender differences as careers unfold

**Figure 5:**
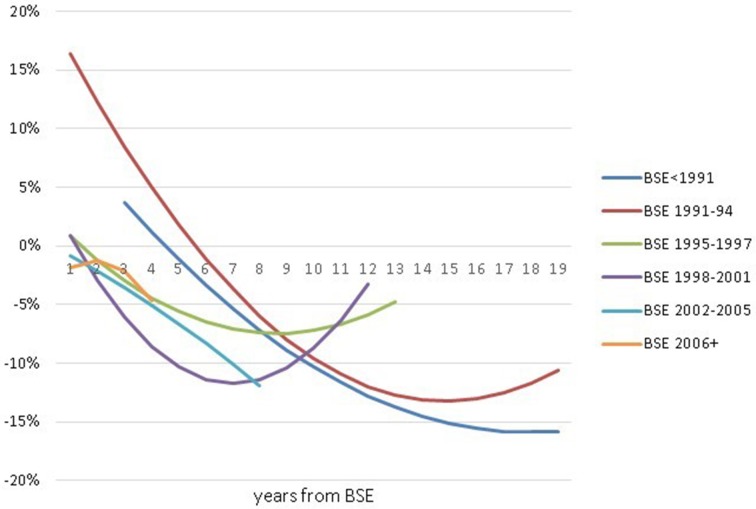
**Cohort-specific estimated time-paths of gender gaps in retention in engineering, calculated as the difference of the female and male retention rates by year-from-BSE predicted from regression**. Data Source: NSF SESTAT Surveys 1993–2010.

A final way we illustrate the differences between cohorts over careers is via six regressions, one for each of the six cohorts, each one on all years of data that we observe that cohort. In each regression, we estimated the likelihood of a cohort remaining in engineering as a function of the regular covariates (race, field dummies, year dummy, citizenship dummy) as well as on two polynomials functions (quartics) for year from BSE, one for male and one for female. This allows us to predict the gender differences in mobility as careers develop separately for men and women. These gender differences for the whole population are illustrated as Figure 5.

The average for each cohort illustrates similar differences to those found earlier, i.e., the cohorts of BSE < 1991, BSE 1995–1997 and particularly BSE 1998–2001 have negative gender differences and the cohort of 1991–1994 has the most positive gender difference.

However, this figure adds interesting information on patterns as careers develop, although we are reluctant to base too much of our analysis on this figure because the size of some cohorts at some post-BSE years is quite small. The earliest two cohorts have gender differences that start with women being more likely to be in engineering, but these differences becomes increasingly negative as they age and many have children. Interestingly, for the cohort of 1991–1994 this trend reverses and the gender gap begins narrowing at 16 years post-BSE, presumably when children's caregiving needs fall.

All later cohorts start at zero gender difference but immediately after, a gender gap appears and widens at careers develop, particularly due to women dropping out of the full-time labor force. The most enigmatic pattern is shown by the 1998–2001 cohort, with a strong U-shaped pattern bottoming out at year 7–8^13^. This reflects a reverse pattern in women's tendency to leave the labor force (also evident in the Table 3 averages), where women's probability of being out of the labor force first decreases and then increases^14^, a pattern that may reflect macroeconomic conditions during the 2000s.

#### Alternative measures of retention

It is possible that our definition of “engineering” jobs based on the NSF engineering occupations classifications is too narrow, since engineering is a field that may be used in a variety of other jobs. If we are allowed to use a more expansive definition of an “engineering job”—including jobs that are “engineering-related” (e.g., engineering technicians, architects) and management jobs “requiring technical expertise in engineering or the natural sciences”—we find generally the same qualitative gender differences in retention, although the broader measure leads to somewhat more negative gender gaps. The few qualitative differences from Table 4 are in later cohorts: 2006–2009 BSEs working full-time with controls no longer have a significantly positive coefficient at 1–2 years; at 3–4 years, 2006–2007 BSEs—but not its full-time subset—now have significantly negative coefficients; and the 2002–2003 cohort now has significantly negative retention gender differences at 7–8 years, but again not for its full-time subset.

#### Synthesis of cohort differences

Our main research question was to investigate whether the latest cohorts are unusual in terms of gender differences in retention, or more generally whether we observe a time trend across cohorts. We find no evidence that the gender differences in the cohort of the last half of the 2000s were consistently and significantly different than cohorts of the preceding decade. We tested and rejected that the gender gap was significantly different between the 2006+ cohort and the preceding one (2002–2005) at both the 1–2 and the 3–4 year stages (We do not observe BSEs from the last half of the 2000s at the 7–8 year stage).

Moreover, Figure 4 and Table A1 in the Supplementary Material show individual cohort-year gender retention gaps with variations from 1998 BSE and later that look more like noise than trend. We have statistically tested for general time trends in cohort-year gender retention gaps in any of the 9 time-series of Table A1 in the Supplementary Material (corresponding to retention by the whole population, by the full-time working population, and leaving the labor force, at each of the 3 career-stages)^15^. The only significant time-trend we find (at *p* ≤ 0.10 levels) is a trend toward larger negative gender differences in retention over time at the 1–2 years post-BSE stage (for both the whole population and the FT population). However, this estimated time trend is entirely due to the fact at the 1–2 year point, the 1991–1994 cohort—where women remain more than men—is the earliest cohort observed. This trend disappears at the career stages that include pre-1991 cohorts. Moreover, excluding the 1991–1994 cohort, there are no significant time trends in any of 9 time-series (as evident in Figure 4). The one other time-series that approaches being significant (with or without the 1991–1994 cohort) is a slightly decreasing tendency to leave the labor force at the 7–8 year career stage (both *p* = 0.11).

Our results highlight two cohorts as being unusual: (1) the cohort who received their BSE's 1991–1994, where in regression results women were more likely than men to stay in engineering at each career stage, for the whole population as well as for full-time workers only; and (2) the cohort of 1998–2002, where in women were substantially less likely than men to remain in engineering at the 7–8 year stages, even among those women working full-time.

We analyzed whether these two cohorts were unlikely to have occurred randomly. If we assume that all of annual coefficients on the gender retention differences at the three different career stages from Table A1 in the Supplementary Material were generated randomly from a normal distribution, we can examine whether the coefficients for these cohorts were sufficiently different from the mean coefficient such that they were less than 10% likely to have been generated randomly so that the coefficients appear in the normal distribution's top or bottom 5% tail. We found coefficients in the top 5% of the distribution at various career stages in the years 1991, 1992, and 1993; we found coefficients in the bottom 5% in 1999 and 2000 only at 7–8 year stage; and finally we found coefficients for 2002 in the bottom tail, again at the 7–8 year stages. In an alternative test to distinguish outliers (looking at distributions within each column in Table A1 in the Supplementary Material separately), the early 90s remained as outliers. However, neither 1999, 2000 nor 2002 were in the hypothetical bottom tail. We conclude that the finding that women with early 1990s BSEs were less likely than men to leave engineering at all three career points is quite robust, but that we are less certain that women with 1998–2002 BSEs were unusually likely to leave engineering at the 7–8 year point.

### Where do they go?

#### Leaving the labor force for family reasons

**Figure 6:**
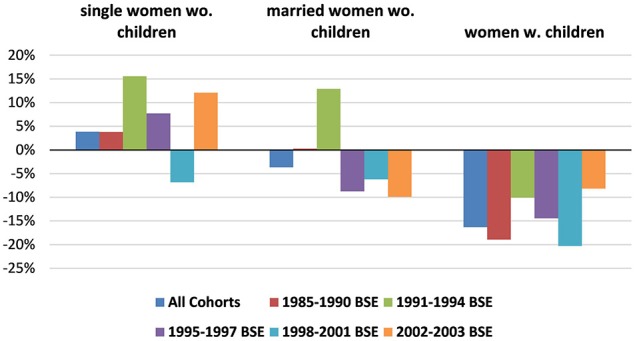
**Gender gap in retention in engineering by family-status of women at 7–8 years post-BSE for all BSEs (comparison group: all men)**. Data Source: NSF SESTAT Surveys 1993–2010.

Women leaving the labor force are responsible for a good portion of the gender retention differences observed, and variation in the rate of leaving the labor force over the career cycle and across cohorts propel some of our findings. As Table 1 showed, an average 10.3% of female BSEs did so by 7–8 years. For many women, leaving the labor force coincides with having children. For instance, of the women out of the labor force at 7–8 years post-BSE, 72% had children compared to only 29% of BSE women working full time. In this section, we investigate whether cohort differences observed were a result of changing fertility decisions over cohorts such as postponing child-bearing or marriage. To do this, we add family terms, specifically interaction terms for female X cohort X family-status to our regressions of Table 4 (first columns). We combine males of all family types into a single category because few men leave the labor force irrespective of family status. We divide women into three categories: single women without children, married women without children, and women with children^16^. The coefficients of the three family-status terms by cohort are graphed in Figure 6, where a value of 0 means that the women were similar to men. Figure 6 shows that single women without children are *more* likely than men to remain in engineering at the 7–8 year point for every cohort except for the unusual 1998–2001 cohort. This is true both overall and among the subset working full time. For the cohort with 1991–1994 BSEs, single childless women's advantage over men in staying (15.6 *ppt*.) is more than twice the average for all women in Table 4. This indicates that the 1991–1994 BSEs were not outliers because they tended to be single or childless: instead, they were outliers within the group of single (or married) women without children. For the remaining three cohorts of single childless women, the gender advantage is not always significant, but positive and jointly significant.

In contrast, women with children (right-hand set of histogram bars) are much less likely than men to remain in engineering at the 7–8 year point for all cohorts. For these women, the magnitudes of the gender differences for the four cohorts with female retention disadvantages are between 70 and 230% greater than in Table 4 gender differences, with 1998–2001 being largest and the earliest 1985–1990 cohort second largest. Gender differences for the fifth cohort—1991–1994 BSEs—switch from significantly positive to insignificantly negative.

Finally, marriage alone—even in the absence of children—seems to affect women in some cohorts. Thus, in the 1995–1997 and 2002–2003 cohorts, childless married women are significantly less likely to continue in engineering than single childless women.

We have also re-estimated our regression of the likelihood of *leaving the labor force* including gender-family status interactions and found that women of all family situations are significantly more likely than men to leave the labor force, although by far the largest differences are for those women with children. Specifically, married women without children are least likely to leave (gender difference 1.9 *ppt*.), single women without children are slightly (but significantly) more likely to leave (gender difference 3.3 *ppt*.), but women with children are a huge 18.4 *ppt*. more likely than men to leave the labor force by the 7–8 year career stage. Dividing into cohorts, the impact of children on remaining in the labor force has no time trend, with gender differences ranging from 13.8 *ppt*.–22.6 *ppt*.

**Figure 7:**
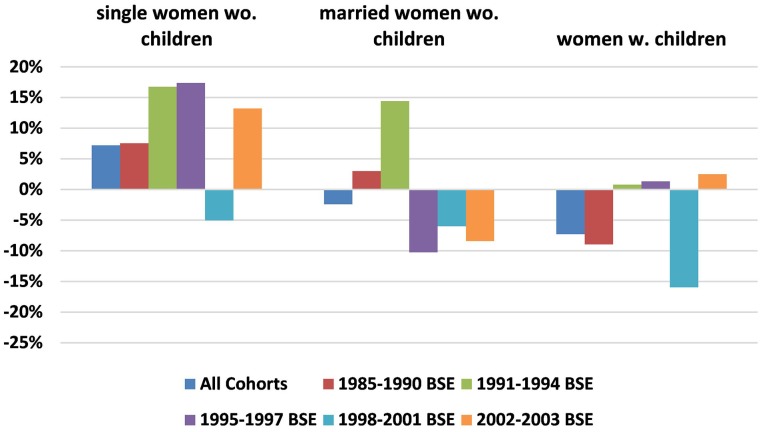
**Gender gap in retention in engineering by family-status of women at 7–8 years post-BSE for BSEs working full-time (comparison group: men working full-time)**. Data Source: NSF SESTAT Surveys 1993–2010.

Even for those who remain working full-time, children may lead women to leave the engineering occupation if engineering is particularly demanding in terms of hours or hours-inflexibility (Goldin, 2014). Figure 7 illustrates the gender engineering retention differences of those working full time, by family status.

For women without children—both single and married—the gender differences for those working full time are similar to the ones in Figure 6, with one difference in scale: single childless women with 1995–1997 BSEs who work full time are now *much* more likely (17.4 *ppt*.) to remain in engineering than comparable men.

For women with children working full time (right-hand set of bars), however, there are basically zero gender differences for 3 of the 5 cohorts (including the 1991–1994 cohort). Children did not deter these cohorts of women from remaining in engineering.

Among women with children working full-time, both the exceptional cohort of 1998–2001 BSEs and the earliest cohort (1985–1990) continue to have large and significant female disadvantages. But while the 1998–2001 cohort of women is less likely than men to remain in engineering irrespective of their family status, it takes marriage and/or children to deter the earliest 1985–1990 cohort. This may be representative of the period before 1985 as well, where marriage and children have a large impact not just on whether a women works, but on whether she works in engineering jobs.

To summarize, single women without children are actually more likely than men to remain in engineering. Children have the greatest effect pulling women out of the labor force and thus out of engineering jobs. Among women and men working full-time, women with children in three cohorts behave like men. Children and marriage deter even full-time working women from remaining in engineering for the earliest cohort. The cohort of women with 1998–2001 BSEs has the least attachment to engineering irrespective of family situation. The cohort of women with 1991–1994 BSEs only has a higher likelihood than men of staying in engineering if they have no children.

#### Leaving for other occupations

Even though children explain much of the gender differences in remaining in engineering in most cohorts, we are interested in knowing whether more recent cohorts of women who work full-time are more likely than previous cohorts not just to leave engineering, but to leave all technical or math-intensive fields (chemistry, physics, math, geology, economics) STEM jobs. This may occur if they were overly encouraged to enter fields that did not particularly interest them.

**Figure 8:**
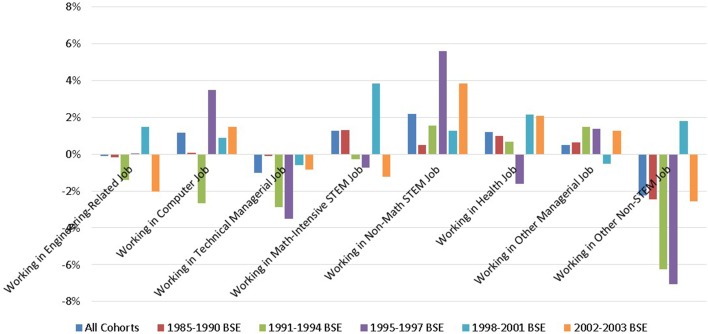
**Cohort-specific gender differences in the probability of being in occupations other than engineering among those working full-time (at 7–8 years post-BSE)**. Data Source: NSF SESTAT Surveys 1993–2010.

For those who have left engineering but remain working full-time at the 7–8 year post-BSE point, Figure 8 shows the gender difference in the percent of full-time working BSEs working in various types of occupations. The largest gender difference across all cohorts is that women are more likely than men to move to non-intensive STEM occupations, in which we include biology, psychology, and social science jobs. In fact, women are on average more than four times as likely as men to move from engineering BSEs to being in these non-mathematical STEM occupations, a sector that grew considerably over the study period and that increasingly attracted women majors (Figure 1). Women are also significantly more likely than men to move to health jobs (which included health management). We note that women in the latest cohort observed at the 7–8 year point (2002–2003) are more likely to move to both health and non-math STEM jobs.

While women are more likely to move to non-math-intensive STEM jobs, men are more likely to move to non-STEM jobs.

On average, women and men are equally likely to move out of the more technical, math-intensive jobs shown in the first, second and fourth sets of bars of Figure 8. Isolating cohorts, the 2002–2003 cohort does not demonstrate a consistent tendency to move from these jobs, suggesting that recent cohorts of women are not running away from technical/math fields. The only cohort with consistent behavior across these sectors is that of 1991–1994: although women in this cohort were more likely to stay in engineering than men, they were less likely to go into other technical, math-intensive jobs, perhaps because the more technical-focused women of this cohort remained in engineering.

The third set of bars represents technically-oriented managerial jobs. Men are clearly more likely to move to these jobs. However, women have a small advantage in moving to non-STEM management jobs. We presume that this difference is likely to be dominated by opportunities for advancement rather than choice.

## Summary and discussion

This paper uses NSF longitudinal SESTAT data to study recent cohort differences in gender-specific careers of people who received BSE. It concentrates on the first 8 years of people's post-bachelors career because we cannot observe many cohorts for longer periods. Our analysis misses data for certain cohorts due to the irregular periodicity of the SESTAT surveys. Nevertheless, the sample is large and complete enough to find significant results related to changes in gender differences over cohorts.

The paper's major contribution is to consider whether there are time patterns in the gender differences in leaving engineering for other jobs within the first 8 years after receipt of a Bachelors in Engineering (BSE). This is of particular interest if recent cohorts of female BSEs are opting out of engineering because they feel it is a bad match.

We find that overall, women are more likely than men to leave engineering by 3–4 years post-BSE for some cohorts and by 7–8 years post-BSE for all but one cohort. However, there are no clear time trends in this gender difference. Particularly, retention of women in the most recent cohorts is neither particularly high nor low.

We find that much of this gender difference is attributable to women leaving the labor force, similar to the findings of several others (Society of Women Engineers, 2009; Hunt, 2010). Thus, at 7–8 years post-BSE, the gender difference in leaving the labor force completely is 8.5 *ppt*., more than enough to account for the overall gender difference. Gender differences in leaving the labor force for BSEs was shown to be similar to that among all college graduates (calculated from American Community Survey). There is a small time trend toward women in later cohorts being less likely to leave the labor force at the 7–8 year career point.

Family status is of key importance. Women with children are most likely to leave the labor force and therefore engineering. Single women without children are actually less likely than men to leave engineering (by the 7–8 year point) for 4 of the 5 cohorts.

Similarly, women who remain working full-time on average are somewhat *more* likely than full-time men to remain in engineering jobs through the 3–4 year post-BSE point, and equally or more likely 7–8 years post-BSE for four of the five cohorts. Dividing by family status, single women without children who work full-time are more likely to remain for four of the five cohorts at the 7–8 year point and even women with children are equally likely to remain for 3 of the 5 cohorts.

Two cohorts stand out. The first is the cohort with BSEs in the early 90s (1991–1994) where women were 8–12% *more* likely than men to remain in engineering jobs through the 7–8 year point. Having children did discourage even these women to leave the labor force and thus engineering, but those with children who remained working full-time were equally likely as men to remain in engineering. Moreover, unlike the previous cohort (BSE < 1991), Figure 5 indicates that this cohort's gender gap in retention (not limited to full-time workers) bottoms out at 16 years post-BSE, again reflecting the unusual aspect of the 1991–1994 cohort in that they returned to engineering once their child-rearing responsibilities lightened.

On the other hand, the cohort of women with 1998–2001 BSEs seems more likely than any of those studied to leave engineering jobs for other jobs, particularly by the 7–8 year point, irrespective of family status. The unusual pattern of this cohort of women's labor force commitment (with more out of the labor force in the years immediately post-BSE than some years later, later followed by increased exit) suggested the possibility of macroeconomic factors' influencing this cohort.

The earliest cohort picked up by SESTAT at the 7–8 year point are 1985–1990 BSEs. Children and marriage lead this cohort of women to be more likely to leave engineering even if they remain working full-time. This suggests an improvement in the environment of engineering jobs since 1990 making it easier for mothers to remain in their jobs, perhaps the result of the 1993 Family and Medical Leave Act.

Full-time working women who left engineering were equally likely as full-time men to remain in technical, math-intensive jobs, with no clear time trend, again suggesting that recent cohorts of women BSEs are not more ill-suited to mathematical/technical work than previous ones.

In sum, women who get BSE behave similarly to other college-educated women in terms of their likelihood to leave the labor force for family reasons. There has been a slight decrease over time in this likelihood. Of those who remain working full-time, women and men are equally likely to stay connected to engineering and, if they do leave engineering, to use their technical skills. There is no evidence that later cohorts of women who work full-time are different than previous cohorts of women. With the large growth in female engineering majors and an unchanging rate of retention, we can expect future growth of women in engineering careers.

### Conflict of interest statement

The authors declare that the research was conducted in the absence of any commercial or financial relationships that could be construed as a potential conflict of interest.

## Footnotes

^1^This number is computed from Hunt's figures although she herself did not make this calculation.

^2^This includes: adding new cohorts; adding others if needed to balance others who had dropped out of the survey; non-response for one wave (people were dropped if they did not respond for 2 waves); aging out at age 75 etc.

^3^We limit this analysis to first bachelors because we are interested in those who originally chose engineering as a field in college, not those who came to it later. Also, those for whom the engineering BS is not their first bachelors degree may be at a different career stage. The vast majority of BSEs are first bachelors.

^4^After a handful of years from the BSE when some complete a masters, there is no distinction between engaged in and working in engineering.

^5^Also within 30 years of their bachelors. Calculated by the authors from the Bureau of the Census's American Community Survey.

^6^Unemployment rates of BSE engineers are similar for men and women.

^7^Thus estimating the gender gap at 7–8 years from BSE, controlling for race variables alone made the gender coefficient fall. Our race variables are defined as follows: We separated out non-black Hispanics and we combined black with other under-represented races such as Native American. Asians were a separate category. There were no gender differences in the percentage of men and women who were Hispanic.

^8^We use a range for beginning and end points because of the spacing of SESTAT surveys. To further increase our sample size, if someone was not observed in years 7 or 8 but was observed in year 9 still in engineering, we also include them in this panel.

^9^Analysis for 1984 BSEs uses SESTAT 1993 for the 9-year point and SESTAT 1999 for the 15-year point. Analysis of 1995 BSEs uses SESTAT 2003 and 2010 for the 8 and 15 year points, respectively. Those with 1985, 1986, 1989, and 1993 BSEs could not be observed at both career points so are not included in the Panel D analysis.

^10^Recall that SESTAT skips from 1999 to 2003 and then to 2006.

^11^As above, the three individual years 1991, 1992, and 1993 were separately significant in Table A1.

^12^Recall that there were no SESTAT surveys 2000–2002.

^13^The cohort of 1995–1997 BSEs also has a U-shape, but this nonlinearity is insignificant (*p* = 0.45) in sharp contrast to the 1998–2001 BSE cohort where the nonlinearity has a *p*-value of 0.02.

^14^This remains the case even if we exclude people who are currently in school. The same pattern of labor force participation is seen to a much smaller extent among men.

^15^To do this, we run regressions of the coefficients on a time trend variable. Each regression has 12–14 observations depending on the career stage.

^16^There are too few single women with children to separate them from married women with children. We have tried dropping them and results are similar, not surprising in light of the fact that children rather than marital status dominates the results for married women.

## References

[B1] Carr R. Bennett L. Strobel J. (2012). Engineering in the K-12 STEM standards of the 50 US States: an analysis of presence and extent. J. Eng. Educ. 101, 539–564. 10.1002/j.2168-9830.2012.tb00061.x

[B2] Ceci S. J. Ginther D. Kahn S. Williams W. (2014). Women in academic science: a changing landscape. Psychol. Sci. Public Interest 15, 75–141. 10.1177/1529100614541236 26172066

[B3] Glass J. L. Sassler S. Levitte Y. Michelmore K. (2013). What's so special about STEM? A comparison of women's retention in STEM and professional occupations. Social Forces. 92, 723–756. 10.1093/sf/sot092 25554713 PMC4279242

[B4] Goldin C. (2014). A grand gender convergence: its last chapter. Am. Econ. Rev. 4, 1091–1119. 10.1257/aer.104.4.1091

[B5] Hunt J. (2010). Why do women leave science and engineering? National Bureau of Economic Research Working Paper No. 15853. Available online at: http://www.nber.org/papers/w15853, (Accessed March, 2010).

[B6] Morgan L. (2000). Is engineering hostile to women? An analysis of data from the 1993 National Survey of College Graduates. Am. Sociol. Rev. 65, 316–321. 10.2307/2657444

[B7] National Academy of Engineering National Research Council. (NAE/NRC) (2014). Sara Frueh, rapporteur. Career Choices of Female Engineers: A Summary of a Workshop. Washington, DC: National Academies Press.

[B8] Preston A. E. (1994). Why have all the women gone? A study of exit of women from the science and engineering professions. Am. Econ. Rev. 84, 1446–1462.

[B9] Preston A. E. (2004). Leaving Science: Occupational Exit from Scientific Careers. New York, NY: Russell Sage Foundation.

[B10] Singh R. Fouad N. Fitzpatrick M. Liu J. Cappaert K. Figuereido C. (2013). Stemming the tide: predicting women engineer's intentions to leave. J. Vocat. Behav. 83, 281–294. 10.1016/j.jvb.2013.05.007

[B11] Society of Women Engineers (SWE) (2009). National Survey About Engineering. (Excerpts from Society of Women Engineers Magazine, 2007, 2008 and 2009). Available online at: http://www.nxtbook.com/nxtbooks/swe/nationalsurveyengineering/

[B12] Xie Y. Shauman K. A. (2003). Women in Science: Career Processes and Outcomes. Cambridge, MA: Harvard University Press.

[B13] Xu Y. J. (2008). Gender disparity in STEM disciplines: a study of faculty attrition and turnover intentions. Res. High. Educ. 49, 607–624. 10.1007/s11162-008-9097-4

