# Supplementary material for: Are recent cohorts of women with engineering bachelors less likely to stay in engineering?
Source: Front Psychol. 2015 Aug 19;6:1144. doi: 10.3389/fpsyg.2015.01144 (PMC4541081; doi:10.3389/fpsyg.2015.01144)
Supplement: Supplementary file 2 [file DataSheet2.DOCX]

**Are recent cohorts of women with engineering bachelors less likely to stay in engineering? Shulamit Kahn and Donna K. Ginther**

**Supplementary Material: Full Regression Results**

**Variable Definitions: Regression Output**

female : female (dummy variable)

femcoh1: female*cohort pre-1991 BSE (dummy variable)

femcoh2: female* 1991-1994 BSE cohort (dummy variable)

femcoh3: female* 1995-1997 BSE cohort (dummy variable)

femcoh4: female* 1998-2001 BSE cohort (dummy variable)

femcoh5: female* 2002-2005 BSE cohort (dummy variable)

femcoh6: female* 2006-2009 BSE cohort(dummy variable)

cfemXb1y_1_1985: * received BSE in 1985 (Similar variable for other BSE years)

reyr_rn: survey year (dummy variables). Note that given the structure data, this serves the

same role as would separate cohort dummies.

cit: citizenship status. 1 (omitted category): US native 2: US citizen naturalized

3: permanent resident 4: temporary resident (dummy variables)

race: 1 (omitted category): White 2: Hispanic 3: Black 4:Asian (dummy variables)

parentba: Parent had a BA or higher (dummy variable)

missingparentba: Information on parent’s education missing (dummy variable)

ba1field: Field of first BA (dummy variables)

517210: Aerospace, aeronautical and astronautical engineering (excluded category)

527250: Chemical engineering

537230: Architectural engineering

537260: Civil engineering

547270: Computer and systems engineering

547280: Electrical, electronics and communications engineering

557330: Industrial and manufacturing engineering

567350: Mechanical engineering

577220: Agricultural engineering

577240: Bioengineering and biomedical engineering

577290: Engineering sciences, mechanics and physics

577300: Environmental engineering

577310: Engineering, general

577320: Geophysical and geological engineering

577340: Materials engineering, including ceramics and textiles

577360: Metallurgical engineering

577370: Mining and minerals engineering

577380: Naval architecture and marine engineering

577390: Nuclear engineering

577400: Petroleum engineering

577410: OTHER engineering

**Full Regression Results – Table 3**

**Probability of Remaining in Engineering 1-2 Years Post-BSE**

**Population: All**

Source | SS df MS Number of obs = 16857

-------------+------------------------------ F( 36, 16820) = 56.15

Model | 429.376929 36 11.9271369 Prob > F = 0.0000

Residual | 3572.68071 16820 .212406701 R-squared = 0.1073

-------------+------------------------------ Adj R-squared = 0.1054

Total | 4002.05764 16856 .237426296 Root MSE = .46088

---------------------------------------------------------------------------------

engagedeng | Coef. Std. Err. t P>|t| [95% Conf. Interval]

----------------+----------------------------------------------------------------

female | .0127223 .0094467 1.35 0.178 -.0057942 .0312389

|

refyr_rn |

1995 | -.0104329 .01452 -0.72 0.472 -.0388937 .0180279

1997 | .0024347 .0147852 0.16 0.869 -.0265459 .0314153

1999 | .0511176 .0148207 3.45 0.001 .0220674 .0801677

2003 | .0375896 .0148822 2.53 0.012 .0084188 .0667603

2006 | .0584426 .0144386 4.05 0.000 .0301413 .0867438

2008 | .0614039 .01442 4.26 0.000 .0331393 .0896686

2010 | .1016196 .0141322 7.19 0.000 .073919 .1293202

|

cit |

2 | -.0343974 .014788 -2.33 0.020 -.0633834 -.0054114

3 | -.0602253 .0211178 -2.85 0.004 -.1016184 -.0188322

4 | .033815 .0180412 1.87 0.061 -.0015476 .0691777

|

race |

2 | -.0509095 .013832 -3.68 0.000 -.0780217 -.0237974

3 | -.0748193 .0150044 -4.99 0.000 -.1042295 -.0454091

4 | -.0911979 .0121143 -7.53 0.000 -.1149432 -.0674527

|

ba1field |

527250 | .0626899 .0221728 2.83 0.005 .0192289 .1061509

537230 | .0430792 .0447473 0.96 0.336 -.0446302 .1307886

537260 | .1585508 .0205691 7.71 0.000 .1182332 .1988684

547270 | -.391239 .022872 -17.11 0.000 -.4360705 -.3464075

547280 | .0195686 .0197818 0.99 0.323 -.0192059 .0583431

557330 | -.0403832 .0241687 -1.67 0.095 -.0877563 .00699

567350 | .1360824 .0197153 6.90 0.000 .0974384 .1747265

577220 | -.0229263 .0476656 -0.48 0.631 -.1163558 .0705033

577240 | -.2018407 .0263124 -7.67 0.000 -.2534158 -.1502656

577290 | -.17679 .0344806 -5.13 0.000 -.2443756 -.1092044

577300 | .112203 .0405159 2.77 0.006 .0327876 .1916185

577310 | -.0197426 .0301035 -0.66 0.512 -.0787487 .0392635

577320 | -.0322907 .0944798 -0.34 0.733 -.2174811 .1528998

577340 | -.0141002 .0334902 -0.42 0.674 -.0797445 .051544

577360 | .1877037 .0770538 2.44 0.015 .0366701 .3387373

577370 | .1046993 .0866949 1.21 0.227 -.0652318 .2746305

577380 | -.0588907 .0781366 -0.75 0.451 -.2120466 .0942653

577390 | .0261824 .0553177 0.47 0.636 -.0822461 .1346108

577400 | .1278566 .0634767 2.01 0.044 .0034357 .2522775

577410 | -.0935794 .033205 -2.82 0.005 -.1586646 -.0284942

|

parentba | -.0508131 .0076335 -6.66 0.000 -.0657757 -.0358506

missingparentba | -.3441709 .1135703 -3.03 0.002 -.5667807 -.1215612

_cons | .6060944 .02096 28.92 0.000 .5650105 .6471782

**Probability of Remaining in Engineering 1-2 Years Post-BSE**

**Population: Working Full Time**

Source | SS df MS Number of obs = 13382

-------------+------------------------------ F( 36, 13345) = 59.42

Model | 400.163257 36 11.115646 Prob > F = 0.0000

Residual | 2496.64639 13345 .18708478 R-squared = 0.1381

-------------+------------------------------ Adj R-squared = 0.1358

Total | 2896.80965 13381 .216486783 Root MSE = .43253

---------------------------------------------------------------------------------

engagedeng | Coef. Std. Err. t P>|t| [95% Conf. Interval]

----------------+----------------------------------------------------------------

female | .0361908 .0100375 3.61 0.000 .0165158 .0558657

|

refyr_rn |

1995 | -.014069 .0152709 -0.92 0.357 -.0440022 .0158641

1997 | -.0064791 .0154788 -0.42 0.676 -.0368198 .0238616

1999 | .0378386 .0154126 2.46 0.014 .0076278 .0680494

2003 | .0611282 .0158536 3.86 0.000 .0300529 .0922036

2006 | .0448454 .0152317 2.94 0.003 .0149891 .0747018

2008 | .0643096 .0150951 4.26 0.000 .0347211 .0938981

2010 | .0772321 .0153932 5.02 0.000 .0470592 .107405

|

cit |

2 | -.0009703 .0158685 -0.06 0.951 -.0320748 .0301341

3 | .0094951 .0233375 0.41 0.684 -.0362497 .05524

4 | -.0068245 .0233018 -0.29 0.770 -.0524994 .0388504

|

race |

2 | -.0301561 .0145385 -2.07 0.038 -.0586535 -.0016586

3 | -.053176 .0161547 -3.29 0.001 -.0848414 -.0215105

4 | -.0805717 .0131561 -6.12 0.000 -.1063595 -.054784

|

ba1field |

527250 | .1004011 .0237902 4.22 0.000 .0537689 .1470332

537230 | .0407757 .0456689 0.89 0.372 -.0487418 .1302932

537260 | .1848287 .0221407 8.35 0.000 .1414297 .2282277

547270 | -.4650709 .0245152 -18.97 0.000 -.5131242 -.4170176

547280 | .0174233 .0214394 0.81 0.416 -.0246011 .0594476

557330 | -.0584985 .025673 -2.28 0.023 -.1088211 -.0081758

567350 | .1488937 .021269 7.00 0.000 .1072034 .190584

577220 | .009494 .0496374 0.19 0.848 -.0878023 .1067904

577240 | -.1551043 .0310996 -4.99 0.000 -.2160639 -.0941447

577290 | -.1402763 .0387134 -3.62 0.000 -.21616 -.0643925

577300 | .182326 .0419315 4.35 0.000 .1001344 .2645176

577310 | -.0122754 .0321093 -0.38 0.702 -.0752141 .0506633

577320 | .1220563 .1091457 1.12 0.263 -.0918846 .3359973

577340 | .0260343 .0365524 0.71 0.476 -.0456137 .0976822

577360 | .257081 .0810747 3.17 0.002 .0981631 .4159989

577370 | .0999142 .0865265 1.15 0.248 -.06969 .2695183

577380 | -.1003074 .0845255 -1.19 0.235 -.2659894 .0653747

577390 | -.0096669 .0639086 -0.15 0.880 -.1349368 .115603

577400 | .1429041 .0672895 2.12 0.034 .0110071 .2748012

577410 | -.0694006 .0351278 -1.98 0.048 -.1382562 -.0005451

|

parentba | -.0211699 .0079665 -2.66 0.008 -.0367854 -.0055544

missingparentba | .0654653 .2298028 0.28 0.776 -.3849808 .5159114

_cons | .6369877 .0226597 28.11 0.000 .5925715 .6814038

---------------------------------------------------------------------------------

**Probability of Leaving the Labor Force: 1-2 Years Post-BSE**

Source | SS df MS Number of obs = 16857

-------------+------------------------------ F( 36, 16820) = 20.54

Model | 40.5924109 36 1.12756697 Prob > F = 0.0000

Residual | 923.175694 16820 .054885594 R-squared = 0.0421

-------------+------------------------------ Adj R-squared = 0.0401

Total | 963.768105 16856 .057176561 Root MSE = .23428

---------------------------------------------------------------------------------

olf | Coef. Std. Err. t P>|t| [95% Conf. Interval]

----------------+----------------------------------------------------------------

female | .0073244 .004802 1.53 0.127 -.0020881 .0167369

|

refyr_rn |

1995 | .0005661 .007381 0.08 0.939 -.0139013 .0150336

1997 | .0096164 .0075158 1.28 0.201 -.0051152 .0243481

1999 | .0043308 .0075338 0.57 0.565 -.0104362 .0190979

2003 | .0240932 .0075651 3.18 0.001 .0092649 .0389215

2006 | .0074421 .0073396 1.01 0.311 -.0069442 .0218285

2008 | .0085358 .0073301 1.16 0.244 -.005832 .0229035

2010 | .0201963 .0071838 2.81 0.005 .0061153 .0342773

|

cit |

2 | .0114455 .0075172 1.52 0.128 -.0032889 .02618

3 | .0322703 .0107348 3.01 0.003 .011229 .0533116

4 | .0490015 .0091709 5.34 0.000 .0310257 .0669774

|

race |

2 | .0172666 .0070312 2.46 0.014 .0034847 .0310485

3 | .0263292 .0076272 3.45 0.001 .0113791 .0412793

4 | .0332374 .006158 5.40 0.000 .021167 .0453078

|

ba1field |

527250 | .0231603 .0112711 2.05 0.040 .0010678 .0452528

537230 | -.0413642 .0227464 -1.82 0.069 -.0859494 .0032211

537260 | -.0149255 .0104559 -1.43 0.153 -.0354201 .0055691

547270 | -.0272495 .0116265 -2.34 0.019 -.0500386 -.0044604

547280 | -.0148701 .0100557 -1.48 0.139 -.0345803 .0048401

557330 | -.005058 .0122856 -0.41 0.681 -.0291392 .0190231

567350 | -.0199669 .0100219 -1.99 0.046 -.0396108 -.000323

577220 | .004867 .0242298 0.20 0.841 -.0426259 .05236

577240 | .1638571 .0133754 12.25 0.000 .13764 .1900743

577290 | .0298461 .0175275 1.70 0.089 -.0045097 .0642018

577300 | .001967 .0205954 0.10 0.924 -.0384022 .0423361

577310 | -.0074321 .0153025 -0.49 0.627 -.0374266 .0225624

577320 | .0741646 .0480269 1.54 0.123 -.0199731 .1683023

577340 | .0452422 .017024 2.66 0.008 .0118733 .0786111

577360 | -.0150327 .0391687 -0.38 0.701 -.0918075 .0617421

577370 | -.005303 .0440696 -0.12 0.904 -.091684 .081078

577380 | .0699842 .0397191 1.76 0.078 -.0078694 .1478379

577390 | .0481382 .0281196 1.71 0.087 -.0069792 .1032555

577400 | -.0159143 .032267 -0.49 0.622 -.0791611 .0473325

577410 | .0083585 .0168791 0.50 0.620 -.0247262 .0414432

|

parentba | .0348593 .0038804 8.98 0.000 .0272534 .0424652

missingparentba | .0542415 .0577311 0.94 0.347 -.0589176 .1674005

_cons | .0166899 .0106546 1.57 0.117 -.0041942 .0375741

---------------------------------------------------------------------------------

**Probability of Remaining in Engineering 3-4 Years Post-BSE**

**Population: All**

Source | SS df MS Number of obs = 14506

-------------+------------------------------ F( 36, 14469) = 51.90

Model | 395.752603 36 10.9931279 Prob > F = 0.0000

Residual | 3064.61677 14469 .211805707 R-squared = 0.1144

-------------+------------------------------ Adj R-squared = 0.1122

Total | 3460.36937 14505 .2385639 Root MSE = .46022

---------------------------------------------------------------------------------

engagedeng | Coef. Std. Err. t P>|t| [95% Conf. Interval]

----------------+----------------------------------------------------------------

female | -.0162809 .0102147 -1.59 0.111 -.036303 .0037411

|

refyr_rn |

1995 | .01546 .0158461 0.98 0.329 -.0156004 .0465205

1997 | .0113182 .015584 0.73 0.468 -.0192284 .0418648

1999 | -.0267346 .0159013 -1.68 0.093 -.0579032 .0044341

2003 | .0046794 .015841 0.30 0.768 -.026371 .0357298

2006 | .0400665 .0156653 2.56 0.011 .0093606 .0707724

2008 | .0264524 .0155718 1.70 0.089 -.0040704 .0569751

2010 | .0352498 .0146545 2.41 0.016 .006525 .0639745

|

cit |

2 | -.0190181 .0152134 -1.25 0.211 -.0488382 .010802

3 | .0071753 .0235002 0.31 0.760 -.0388881 .0532387

4 | -.0515711 .0178619 -2.89 0.004 -.0865826 -.0165595

|

race |

2 | -.0605769 .0154523 -3.92 0.000 -.0908654 -.0302883

3 | -.0504902 .0162649 -3.10 0.002 -.0823716 -.0186089

4 | -.1221403 .0134405 -9.09 0.000 -.1484855 -.0957952

|

ba1field |

527250 | .1505006 .024096 6.25 0.000 .1032693 .1977319

537230 | .0130982 .0431084 0.30 0.761 -.0713998 .0975963

537260 | .2688621 .0220272 12.21 0.000 .225686 .3120382

547270 | -.3065284 .0240586 -12.74 0.000 -.3536864 -.2593705

547280 | .0697557 .0207621 3.36 0.001 .0290593 .1104521

557330 | -.0210356 .0250837 -0.84 0.402 -.070203 .0281317

567350 | .1997321 .0208648 9.57 0.000 .1588345 .2406297

577220 | .0178659 .050834 0.35 0.725 -.0817752 .1175069

577240 | -.1463946 .0325699 -4.49 0.000 -.2102357 -.0825535

577290 | -.0655222 .0384375 -1.70 0.088 -.1408646 .0098202

577300 | .1700205 .0456802 3.72 0.000 .0804814 .2595596

577310 | .0288132 .0334849 0.86 0.390 -.0368215 .094448

577320 | -.0887171 .1236813 -0.72 0.473 -.3311483 .1537142

577340 | .0514274 .0359358 1.43 0.152 -.0190112 .1218661

577360 | .2157103 .0656061 3.29 0.001 .0871139 .3443066

577370 | .0881812 .1114065 0.79 0.429 -.1301899 .3065523

577380 | .1306589 .0882835 1.48 0.139 -.0423881 .3037058

577390 | .0618798 .0664399 0.93 0.352 -.068351 .1921105

577400 | .2819092 .0653965 4.31 0.000 .1537238 .4100947

577410 | -.0616332 .0351327 -1.75 0.079 -.1304979 .0072314

|

parentba | -.0137009 .0080575 -1.70 0.089 -.0294946 .0020928

missingparentba | .0432455 .114525 0.38 0.706 -.1812383 .2677292

_cons | .5515356 .022224 24.82 0.000 .5079737 .5950975

---------------------------------------------------------------------------------

**Probability of Remaining in Engineering 3-4 Years Post-BSE**

**Population: Working Full Time**

Source | SS df MS Number of obs = 12501

-------------+------------------------------ F( 36, 12464) = 51.16

Model | 361.00407 36 10.0278908 Prob > F = 0.0000

Residual | 2442.89183 12464 .195995814 R-squared = 0.1288

-------------+------------------------------ Adj R-squared = 0.1262

Total | 2803.8959 12500 .224311672 Root MSE = .44271

---------------------------------------------------------------------------------

engagedeng | Coef. Std. Err. t P>|t| [95% Conf. Interval]

----------------+----------------------------------------------------------------

female | .0184525 .0108341 1.70 0.089 -.002784 .0396891

|

refyr_rn |

1995 | -.0010772 .0165108 -0.07 0.948 -.0334408 .0312865

1997 | -.0166236 .016194 -1.03 0.305 -.0483663 .0151192

1999 | -.0660513 .0164519 -4.01 0.000 -.0982996 -.033803

2003 | -.0198637 .0165069 -1.20 0.229 -.0522198 .0124923

2006 | .0157297 .0163632 0.96 0.336 -.0163447 .0478041

2008 | .0023791 .0162479 0.15 0.884 -.0294693 .0342275

2010 | -.0012922 .0153628 -0.08 0.933 -.0314056 .0288213

|

cit |

2 | .0050609 .0163432 0.31 0.757 -.0269742 .037096

3 | -.0159777 .024861 -0.64 0.520 -.0647091 .0327536

4 | -.0770455 .020198 -3.81 0.000 -.1166366 -.0374544

|

race |

2 | -.0360723 .0161889 -2.23 0.026 -.067805 -.0043396

3 | -.0384205 .0168918 -2.27 0.023 -.0715311 -.00531

4 | -.1065942 .0145683 -7.32 0.000 -.1351504 -.078038

|

ba1field |

527250 | .1756178 .0253549 6.93 0.000 .1259183 .2253172

537230 | -.0120169 .0437413 -0.27 0.784 -.0977566 .0737228

537260 | .2469217 .0229845 10.74 0.000 .2018684 .2919749

547270 | -.3555259 .0250887 -14.17 0.000 -.4047035 -.3063482

547280 | .0552106 .021857 2.53 0.012 .0123676 .0980536

557330 | -.0407341 .0261615 -1.56 0.119 -.0920147 .0105466

567350 | .1962994 .0219214 8.95 0.000 .1533301 .2392686

577220 | -.0419027 .052219 -0.80 0.422 -.14426 .0604545

577240 | -.0621208 .0386366 -1.61 0.108 -.1378545 .0136129

577290 | -.0635378 .0410654 -1.55 0.122 -.1440323 .0169567

577300 | .1765406 .0478858 3.69 0.000 .0826769 .2704042

577310 | .0534915 .0352616 1.52 0.129 -.0156267 .1226097

577320 | -.0155009 .1386208 -0.11 0.911 -.2872191 .2562174

577340 | .0490306 .0379597 1.29 0.197 -.0253763 .1234374

577360 | .2692268 .0693756 3.88 0.000 .13324 .4052137

577370 | .0382943 .1085453 0.35 0.724 -.1744713 .2510599

577380 | .0984155 .0875628 1.12 0.261 -.0732211 .2700521

577390 | .0953976 .0710569 1.34 0.179 -.043885 .2346801

577400 | .2735451 .0664826 4.11 0.000 .143229 .4038613

577410 | -.0784594 .0364523 -2.15 0.031 -.1499115 -.0070074

|

parentba | .0051832 .0082946 0.62 0.532 -.0110756 .0214419

missingparentba | .106262 .1206395 0.88 0.378 -.1302101 .3427341

_cons | .6105471 .023409 26.08 0.000 .5646618 .6564324

---------------------------------------------------------------------------------

**Probability of Leaving the Labor Force: 3-4 Years Post-BSE**

Source | SS df MS Number of obs = 14506

-------------+------------------------------ F( 36, 14469) = 18.75

Model | 29.657931 36 .823831417 Prob > F = 0.0000

Residual | 635.868608 14469 .043946963 R-squared = 0.0446

-------------+------------------------------ Adj R-squared = 0.0422

Total | 665.526539 14505 .04588256 Root MSE = .20964

---------------------------------------------------------------------------------

olf | Coef. Std. Err. t P>|t| [95% Conf. Interval]

----------------+----------------------------------------------------------------

female | .0102328 .0046529 2.20 0.028 .0011126 .019353

|

refyr_rn |

1995 | -.0088729 .007218 -1.23 0.219 -.0230212 .0052753

1997 | -.0119961 .0070986 -1.69 0.091 -.0259103 .0019181

1999 | -.0240638 .0072432 -3.32 0.001 -.0382614 -.0098662

2003 | -.0294549 .0072157 -4.08 0.000 -.0435986 -.0153113

2006 | -.0099972 .0071356 -1.40 0.161 -.0239839 .0039896

2008 | -.014212 .0070931 -2.00 0.045 -.0281154 -.0003086

2010 | -.0210189 .0066753 -3.15 0.002 -.0341033 -.0079346

|

cit |

2 | .0109891 .0069298 1.59 0.113 -.0025943 .0245724

3 | .0095504 .0107045 0.89 0.372 -.0114318 .0305326

4 | .0530731 .0081362 6.52 0.000 .0371251 .0690211

|

race |

2 | .0048263 .0070386 0.69 0.493 -.0089704 .0186229

3 | .0012165 .0074088 0.16 0.870 -.0133057 .0157387

4 | .0303467 .0061223 4.96 0.000 .0183463 .0423471

|

ba1field |

527250 | .0305928 .0109759 2.79 0.005 .0090786 .052107

537230 | -.04198 .0196362 -2.14 0.033 -.0804695 -.0034905

537260 | -.0209042 .0100335 -2.08 0.037 -.0405713 -.0012372

547270 | -.0205842 .0109589 -1.88 0.060 -.042065 .0008966

547280 | -.0024429 .0094573 -0.26 0.796 -.0209804 .0160946

557330 | -.0074647 .0114258 -0.65 0.514 -.0298608 .0149314

567350 | -.0083598 .0095041 -0.88 0.379 -.026989 .0102694

577220 | -.0010264 .0231553 -0.04 0.965 -.0464137 .0443608

577240 | .1986969 .0148358 13.39 0.000 .1696168 .227777

577290 | .0246054 .0175086 1.41 0.160 -.0097137 .0589244

577300 | .0296401 .0208077 1.42 0.154 -.0111457 .0704258

577310 | .0489114 .0152526 3.21 0.001 .0190143 .0788086

577320 | -.0449517 .0563378 -0.80 0.425 -.155381 .0654775

577340 | .0086616 .016369 0.53 0.597 -.0234238 .0407469

577360 | .012804 .0298841 0.43 0.668 -.0457726 .0713806

577370 | -.0311737 .0507465 -0.61 0.539 -.1306434 .068296

577380 | -.0420364 .0402138 -1.05 0.296 -.1208606 .0367878

577390 | .064376 .0302639 2.13 0.033 .0050549 .1236971

577400 | -.018438 .0297886 -0.62 0.536 -.0768275 .0399514

577410 | .0262311 .0160032 1.64 0.101 -.0051372 .0575995

|

parentba | .0281798 .0036702 7.68 0.000 .0209856 .0353739

missingparentba | -.0228997 .052167 -0.44 0.661 -.1251537 .0793544

_cons | .0314537 .0101232 3.11 0.002 .011611 .0512965

---------------------------------------------------------------------------------

**Probability of Remaining in Engineering 7-8 Years Post-BSE**

**Population: All**

Source | SS df MS Number of obs = 11812

-------------+------------------------------ F( 36, 11775) = 39.15

Model | 315.057086 36 8.75158573 Prob > F = 0.0000

Residual | 2632.44246 11775 .223561992 R-squared = 0.1069

-------------+------------------------------ Adj R-squared = 0.1042

Total | 2947.49955 11811 .249555461 Root MSE = .47282

---------------------------------------------------------------------------------

engagedeng | Coef. Std. Err. t P>|t| [95% Conf. Interval]

----------------+----------------------------------------------------------------

female | -.0620179 .0118986 -5.21 0.000 -.085341 -.0386947

|

refyr_rn |

1995 | .0277074 .0176002 1.57 0.115 -.0067918 .0622066

1997 | .0053221 .0175142 0.30 0.761 -.0290086 .0396528

1999 | .0022784 .0181611 0.13 0.900 -.0333204 .0378773

2003 | -.0330852 .0163098 -2.03 0.043 -.065055 -.0011154

2006 | -.0309328 .0172186 -1.80 0.072 -.064684 .0028185

2008 | .083808 .0178631 4.69 0.000 .0487934 .1188225

2010 | -.039357 .0166335 -2.37 0.018 -.0719614 -.0067526

|

cit |

2 | -.0378227 .0174769 -2.16 0.030 -.0720802 -.0035652

3 | -.0551609 .0204511 -2.70 0.007 -.0952484 -.0150734

4 | -.1117108 .0188103 -5.94 0.000 -.1485821 -.0748394

|

race |

2 | .0057634 .0195064 0.30 0.768 -.0324723 .043999

3 | -.0638071 .0204735 -3.12 0.002 -.1039386 -.0236757

4 | -.0693435 .0160213 -4.33 0.000 -.1007478 -.0379391

|

ba1field |

527250 | .1672949 .0270293 6.19 0.000 .1143129 .2202768

537230 | -.1117166 .0428179 -2.61 0.009 -.1956467 -.0277865

537260 | .2873646 .0245913 11.69 0.000 .2391617 .3355675

547270 | -.2107158 .0269817 -7.81 0.000 -.2636044 -.1578272

547280 | .1274551 .0230191 5.54 0.000 .0823339 .1725763

557330 | .0459685 .0282451 1.63 0.104 -.0093965 .1013335

567350 | .2589829 .0234523 11.04 0.000 .2130125 .3049534

577220 | .0662135 .0508829 1.30 0.193 -.0335255 .1659524

577240 | -.0226731 .0437247 -0.52 0.604 -.1083807 .0630346

577290 | .0204977 .0453468 0.45 0.651 -.0683896 .1093849

577300 | .1783324 .0488552 3.65 0.000 .0825682 .2740965

577310 | -.0272773 .0421751 -0.65 0.518 -.1099476 .0553929

577320 | -.1672132 .0896292 -1.87 0.062 -.3429012 .0084748

577340 | .1796296 .0411389 4.37 0.000 .0989905 .2602686

577360 | .2987706 .0783238 3.81 0.000 .145243 .4522982

577370 | .4429592 .0837707 5.29 0.000 .2787547 .6071637

577380 | .1406161 .0654768 2.15 0.032 .0122706 .2689615

577390 | .0049617 .0680985 0.07 0.942 -.1285226 .138446

577400 | .1513566 .0637569 2.37 0.018 .0263825 .2763308

577410 | -.0197025 .0392782 -0.50 0.616 -.0966942 .0572893

|

parentba | -.0140034 .0091099 -1.54 0.124 -.0318603 .0038536

missingparentba | -.0300907 .0931267 -0.32 0.747 -.2126344 .152453

_cons | .4458713 .0244217 18.26 0.000 .3980008 .4937419

---------------------------------------------------------------------------------

**Probability of Remaining in Engineering 7-8 Years Post-BSE**

**Population: Working Full Time**

Source | SS df MS Number of obs = 10585

-------------+------------------------------ F( 36, 10548) = 38.13

Model | 300.895311 36 8.35820309 Prob > F = 0.0000

Residual | 2312.34754 10548 .21922142 R-squared = 0.1151

-------------+------------------------------ Adj R-squared = 0.1121

Total | 2613.24285 10584 .246905031 Root MSE = .46821

---------------------------------------------------------------------------------

engagedeng | Coef. Std. Err. t P>|t| [95% Conf. Interval]

----------------+----------------------------------------------------------------

female | -.0091954 .0131462 -0.70 0.484 -.0349644 .0165736

|

refyr_rn |

1995 | .0339274 .0183543 1.85 0.065 -.0020505 .0699052

1997 | .0065097 .0182712 0.36 0.722 -.0293053 .0423247

1999 | -.0014229 .0188584 -0.08 0.940 -.0383889 .0355431

2003 | -.0122377 .0171229 -0.71 0.475 -.0458017 .0213264

2006 | -.0247374 .0181044 -1.37 0.172 -.0602254 .0107506

2008 | .0890914 .0187122 4.76 0.000 .0524119 .1257708

2010 | -.0562468 .0174443 -3.22 0.001 -.0904408 -.0220528

|

cit |

2 | -.0127916 .0186604 -0.69 0.493 -.0493695 .0237863

3 | -.0620096 .0218445 -2.84 0.005 -.1048289 -.0191903

4 | -.103136 .0207367 -4.97 0.000 -.1437837 -.0624882

|

race |

2 | -.013728 .0204949 -0.67 0.503 -.0539019 .026446

3 | -.0569794 .0216168 -2.64 0.008 -.0993523 -.0146064

4 | -.0742637 .017229 -4.31 0.000 -.1080359 -.0404915

|

ba1field |

527250 | .1755731 .0282422 6.22 0.000 .120213 .2309331

537230 | -.1200615 .0450116 -2.67 0.008 -.2082928 -.0318302

537260 | .297392 .0255951 11.62 0.000 .2472207 .3475632

547270 | -.2337807 .0279608 -8.36 0.000 -.2885892 -.1789722

547280 | .1297992 .0239537 5.42 0.000 .0828455 .176753

557330 | .0395779 .0297861 1.33 0.184 -.0188085 .0979644

567350 | .264196 .0243667 10.84 0.000 .2164328 .3119593

577220 | .0730838 .0529297 1.38 0.167 -.0306685 .1768361

577240 | -.0415567 .0476393 -0.87 0.383 -.1349386 .0518253

577290 | .0356169 .0477311 0.75 0.456 -.0579451 .1291789

577300 | .1597541 .0511699 3.12 0.002 .0594514 .2600569

577310 | -.0190333 .0441554 -0.43 0.666 -.1055863 .0675196

577320 | -.1632288 .0989811 -1.65 0.099 -.3572505 .0307929

577340 | .1922799 .0440283 4.37 0.000 .1059761 .2785837

577360 | .3405935 .0826258 4.12 0.000 .1786313 .5025557

577370 | .4551312 .0876428 5.19 0.000 .2833348 .6269277

577380 | .1777055 .070049 2.54 0.011 .0403962 .3150148

577390 | -.0269843 .0729996 -0.37 0.712 -.1700773 .1161086

577400 | .192775 .0673235 2.86 0.004 .0608082 .3247418

577410 | -.0292035 .0411146 -0.71 0.478 -.1097959 .0513889

|

parentba | -.0079464 .0095306 -0.83 0.404 -.0266283 .0107354

missingparentba | .0267032 .1027683 0.26 0.795 -.1747421 .2281486

_cons | .4585793 .0254118 18.05 0.000 .4087673 .5083913

---------------------------------------------------------------------------------

**Probability of Leaving the Labor Force: 7-8 Years Post-BSE**

Source | SS df MS Number of obs = 11812

-------------+------------------------------ F( 36, 11775) = 16.38

Model | 17.685066 36 .491251832 Prob > F = 0.0000

Residual | 353.217784 11775 .029997264 R-squared = 0.0477

-------------+------------------------------ Adj R-squared = 0.0448

Total | 370.90285 11811 .031403171 Root MSE = .1732

---------------------------------------------------------------------------------

olf | Coef. Std. Err. t P>|t| [95% Conf. Interval]

----------------+----------------------------------------------------------------

female | .0833751 .0043585 19.13 0.000 .0748317 .0919185

|

refyr_rn |

1995 | -.0079058 .006447 -1.23 0.220 -.020543 .0047314

1997 | .009579 .0064155 1.49 0.135 -.0029965 .0221544

1999 | -.0027741 .0066525 -0.42 0.677 -.0158141 .0102659

2003 | -.0008615 .0059743 -0.14 0.885 -.0125722 .0108491

2006 | -.0073851 .0063072 -1.17 0.242 -.0197483 .0049781

2008 | -.0131118 .0065433 -2.00 0.045 -.0259378 -.0002858

2010 | -.0140749 .0060929 -2.31 0.021 -.026018 -.0021317

|

cit |

2 | .0087322 .0064018 1.36 0.173 -.0038165 .0212809

3 | .011715 .0074913 1.56 0.118 -.0029692 .0263992

4 | .0523909 .0068903 7.60 0.000 .0388848 .065897

|

race |

2 | .0063731 .0071453 0.89 0.372 -.0076328 .020379

3 | -.0188667 .0074995 -2.52 0.012 -.0335671 -.0041664

4 | -.002843 .0058687 -0.48 0.628 -.0143465 .0086606

|

ba1field |

527250 | .0087629 .009901 0.89 0.376 -.0106446 .0281704

537230 | .0133834 .0156844 0.85 0.394 -.0173606 .0441273

537260 | .0080881 .0090079 0.90 0.369 -.0095689 .025745

547270 | -.01079 .0098835 -1.09 0.275 -.0301633 .0085833

547280 | .0073511 .008432 0.87 0.383 -.009177 .0238792

557330 | .0224877 .0103463 2.17 0.030 .0022073 .0427682

567350 | -.0008372 .0085907 -0.10 0.922 -.0176764 .016002

577220 | .0039369 .0186386 0.21 0.833 -.0325979 .0404717

577240 | .0163557 .0160165 1.02 0.307 -.0150393 .0477508

577290 | .0611699 .0166107 3.68 0.000 .0286102 .0937297

577300 | -.0235077 .0178958 -1.31 0.189 -.0585865 .0115711

577310 | .0216279 .0154489 1.40 0.162 -.0086546 .0519103

577320 | .0061861 .0328315 0.19 0.851 -.0581691 .0705414

577340 | .0412012 .0150693 2.73 0.006 .0116628 .0707396

577360 | .0393536 .0286903 1.37 0.170 -.0168842 .0955914

577370 | -.0043242 .0306856 -0.14 0.888 -.0644729 .0558246

577380 | -.0220823 .0239844 -0.92 0.357 -.0690958 .0249312

577390 | .0437224 .0249448 1.75 0.080 -.0051734 .0926183

577400 | .03109 .0233544 1.33 0.183 -.0146886 .0768685

577410 | .0144999 .0143878 1.01 0.314 -.0137025 .0427023

|

parentba | .0075122 .003337 2.25 0.024 .0009711 .0140532

missingparentba | .0737841 .0341127 2.16 0.031 .0069176 .1406506

_cons | .0052462 .0089458 0.59 0.558 -.012289 .0227814

---------------------------------------------------------------------------------

**Probability of Remaining in Engineering 9-16 Years Post-BSE if still in Engineering 7-9 Years Post-BSE**

**Population: All**

Source | SS df MS Number of obs = 884

-------------+------------------------------ F( 33, 850) = 2.50

Model | 16.4228761 33 .497662913 Prob > F = 0.0000

Residual | 169.327456 850 .199208772 R-squared = 0.0884

-------------+------------------------------ Adj R-squared = 0.0530

Total | 185.750332 883 .210362777 Root MSE = .44633

---------------------------------------------------------------------------------

engagedeng | Coef. Std. Err. t P>|t| [95% Conf. Interval]

----------------+----------------------------------------------------------------

female | -.0071649 .047388 -0.15 0.880 -.1001761 .0858463

|

refyr_rn |

2003 | -.0781561 .1047119 -0.75 0.456 -.2836804 .1273681

2006 | -.050745 .0927123 -0.55 0.584 -.232717 .1312269

2008 | -.1016897 .1705162 -0.60 0.551 -.4363718 .2329924

2010 | -.1005324 .0319882 -3.14 0.002 -.1633176 -.0377473

|

cit |

2 | -.0173456 .0604176 -0.29 0.774 -.1359307 .1012395

3 | .0583051 .0788705 0.74 0.460 -.0964986 .2131089

4 | -.3927656 .1771993 -2.22 0.027 -.7405651 -.0449662

|

race |

2 | -.0354156 .0740674 -0.48 0.633 -.1807921 .1099609

3 | .0455522 .0844747 0.54 0.590 -.1202512 .2113556

4 | -.1038072 .0634912 -1.63 0.102 -.2284251 .0208107

|

ba1field |

527250 | .0653664 .091939 0.71 0.477 -.1150877 .2458204

537230 | -.0385834 .142528 -0.27 0.787 -.3183314 .2411647

537260 | .1320745 .0865911 1.53 0.128 -.0378829 .302032

547270 | .2387248 .1151239 2.07 0.038 .0127643 .4646852

547280 | .0572178 .0839297 0.68 0.496 -.1075159 .2219516

557330 | -.1329704 .1016535 -1.31 0.191 -.3324916 .0665508

567350 | .1119506 .0838301 1.34 0.182 -.0525876 .2764888

577220 | .0007332 .2180401 0.00 0.997 -.4272269 .4286932

577240 | .3000942 .2967422 1.01 0.312 -.2823392 .8825275

577290 | -.1057102 .1525791 -0.69 0.489 -.4051862 .1937659

577300 | -.3617628 .1532429 -2.36 0.018 -.6625417 -.060984

577310 | .2412283 .294983 0.82 0.414 -.3377521 .8202087

577320 | -.586763 .2731519 -2.15 0.032 -1.122894 -.0506316

577340 | .1853755 .1561509 1.19 0.235 -.121111 .4918621

577360 | .2809839 .1843644 1.52 0.128 -.0808789 .6428467

577370 | .324946 .2561597 1.27 0.205 -.1778338 .8277258

577380 | .3055397 .3598642 0.85 0.396 -.400787 1.011866

577390 | .3255338 .1974223 1.65 0.100 -.0619585 .7130261

577400 | .3142777 .1531098 2.05 0.040 .0137602 .6147952

577410 | -.0568063 .1308822 -0.43 0.664 -.3136966 .200084

|

parentba | .0072939 .031344 0.23 0.816 -.0542269 .0688147

missingparentba | .5002395 .2789517 1.79 0.073 -.0472754 1.047754

_cons | .6871664 .0817844 8.40 0.000 .5266433 .8476896

**Probability of Remaining in Engineering 9-16 Years Post-BSE if still in Engineering 7-9 Years Post-BSE**

**Population: Working Full Time**

Source | SS df MS Number of obs = 848

-------------+------------------------------ F( 33, 814) = 2.56

Model | 16.0989462 33 .487846854 Prob > F = 0.0000

Residual | 154.865529 814 .190252493 R-squared = 0.0942

-------------+------------------------------ Adj R-squared = 0.0574

Total | 170.964475 847 .201847078 Root MSE = .43618

---------------------------------------------------------------------------------

engagedeng | Coef. Std. Err. t P>|t| [95% Conf. Interval]

----------------+----------------------------------------------------------------

female | .0905064 .0507833 1.78 0.075 -.0091754 .1901881

|

refyr_rn |

2003 | -.0923428 .1020778 -0.90 0.366 -.2927095 .1080239

2006 | -.0647581 .0906694 -0.71 0.475 -.2427315 .1132153

2008 | -.0977734 .1680904 -0.58 0.561 -.427715 .2321683

2010 | -.1189551 .032079 -3.71 0.000 -.1819225 -.0559877

|

cit |

2 | -.0334123 .0596252 -0.56 0.575 -.1504496 .083625

3 | .0511222 .0768808 0.66 0.506 -.0997858 .2020302

4 | -.3486338 .2387422 -1.46 0.145 -.8172567 .1199891

|

race |

2 | -.0198516 .0768087 -0.26 0.796 -.1706182 .1309149

3 | .073912 .0837213 0.88 0.378 -.0904231 .2382471

4 | -.1247951 .0629242 -1.98 0.048 -.248308 -.0012822

|

ba1field |

527250 | -.1088073 .0986377 -1.10 0.270 -.3024215 .084807

537230 | -.3655958 .1575522 -2.32 0.021 -.6748522 -.0563394

537260 | -.0447692 .0940798 -0.48 0.634 -.2294368 .1398984

547270 | .1119123 .1205391 0.93 0.353 -.1246919 .3485164

547280 | -.1315256 .0922133 -1.43 0.154 -.3125295 .0494782

557330 | -.3196426 .1077001 -2.97 0.003 -.5310452 -.10824

567350 | -.0610173 .0916453 -0.67 0.506 -.2409062 .1188716

577220 | -.1880263 .2153478 -0.87 0.383 -.6107286 .2346761

577240 | .0499124 .290531 0.17 0.864 -.5203659 .6201907

577290 | -.3251046 .1533837 -2.12 0.034 -.6261787 -.0240305

577300 | -.2798543 .1893727 -1.48 0.140 -.6515708 .0918621

577310 | .0617169 .2883528 0.21 0.831 -.5042858 .6277195

577320 | -.8390489 .2681025 -3.13 0.002 -1.365303 -.3127952

577340 | .0295903 .1581346 0.19 0.852 -.2808093 .3399899

577360 | .0997516 .1834678 0.54 0.587 -.2603741 .4598773

577370 | .1342383 .2514246 0.53 0.594 -.3592788 .6277553

577380 | .1325024 .3507002 0.38 0.706 -.555881 .8208857

577390 | .1029712 .1962737 0.52 0.600 -.282291 .4882334

577400 | .0718936 .1544331 0.47 0.642 -.2312405 .3750277

577410 | -.2420754 .1335283 -1.81 0.070 -.5041757 .020025

|

parentba | -.0285436 .03145 -0.91 0.364 -.0902763 .0331891

missingparentba | .48711 .270339 1.80 0.072 -.0435337 1.017754

_cons | .8960412 .0906378 9.89 0.000 .7181298 1.073953

**Probability of Leaving the Labor Force 9-16 Years Post-BSE if still in Engineering 7-9 Years Post-BSE**

Source | SS df MS Number of obs = 884

-------------+------------------------------ F( 33, 850) = 9.42

Model | 6.98752774 33 .211743265 Prob > F = 0.0000

Residual | 19.1099878 850 .022482339 R-squared = 0.2677

-------------+------------------------------ Adj R-squared = 0.2393

Total | 26.0975155 883 .02955551 Root MSE = .14994

---------------------------------------------------------------------------------

olf | Coef. Std. Err. t P>|t| [95% Conf. Interval]

----------------+----------------------------------------------------------------

female | .1053047 .0159197 6.61 0.000 .0740581 .1365512

|

refyr_rn |

2003 | -.0252369 .0351773 -0.72 0.473 -.0942815 .0438076

2006 | -.0151227 .0311461 -0.49 0.627 -.076255 .0460097

2008 | .0208983 .0572838 0.36 0.715 -.0915361 .1333326

2010 | -.0096743 .0107462 -0.90 0.368 -.0307665 .011418

|

cit |

2 | -.0256388 .0202969 -1.26 0.207 -.0654767 .0141991

3 | -.0170561 .026496 -0.64 0.520 -.0690614 .0349493

4 | .480073 .059529 8.06 0.000 .363232 .596914

|

race |

2 | .0371085 .0248825 1.49 0.136 -.0117298 .0859468

3 | .0138708 .0283787 0.49 0.625 -.0418298 .0695714

4 | -.0179683 .0213295 -0.84 0.400 -.059833 .0238963

|

ba1field |

527250 | -.2292893 .0308863 -7.42 0.000 -.2899117 -.1686669

537230 | -.2575962 .0478814 -5.38 0.000 -.3515758 -.1636166

537260 | -.2200324 .0290897 -7.56 0.000 -.2771285 -.1629363

547270 | -.1732556 .0386752 -4.48 0.000 -.2491656 -.0973456

547280 | -.2198346 .0281956 -7.80 0.000 -.2751759 -.1644934

557330 | -.1983083 .0341498 -5.81 0.000 -.2653362 -.1312804

567350 | -.2273563 .0281622 -8.07 0.000 -.2826319 -.1720807

577220 | -.2325521 .0732492 -3.17 0.002 -.3763226 -.0887816

577240 | -.2985518 .0996887 -2.99 0.003 -.4942167 -.102887

577290 | -.2633782 .051258 -5.14 0.000 -.3639853 -.1627711

577300 | .1740391 .051481 3.38 0.001 .0729943 .2750839

577310 | -.213674 .0990977 -2.16 0.031 -.4081788 -.0191692

577320 | -.3060837 .0917637 -3.34 0.001 -.4861936 -.1259737

577340 | -.1941301 .0524579 -3.70 0.000 -.2970923 -.0911679

577360 | -.221684 .0619361 -3.58 0.000 -.3432495 -.1001184

577370 | -.2308617 .0860552 -2.68 0.007 -.3997674 -.061956

577380 | -.2104532 .1208941 -1.74 0.082 -.4477392 .0268328

577390 | -.2707221 .0663228 -4.08 0.000 -.4008977 -.1405465

577400 | -.2897049 .0514363 -5.63 0.000 -.3906619 -.1887479

577410 | -.2302161 .0439691 -5.24 0.000 -.3165167 -.1439154

|

parentba | -.0443693 .0105298 -4.21 0.000 -.0650368 -.0237017

missingparentba | -.0384748 .0937121 -0.41 0.681 -.222409 .1454594

_cons | .2548225 .027475 9.27 0.000 .2008958 .3087492

---------------------------------------------------------------------------------

**Full Regression Results – Table 4**

**Probability of Remaining in Engineering 1-2 Years Post-BSE**

**Population: All**

Source | SS df MS Number of obs = 16857

-------------+------------------------------ F( 40, 16816) = 51.30

Model | 435.222153 40 10.8805538 Prob > F = 0.0000

Residual | 3566.83549 16816 .212109627 R-squared = 0.1087

-------------+------------------------------ Adj R-squared = 0.1066

Total | 4002.05764 16856 .237426296 Root MSE = .46055

---------------------------------------------------------------------------------

engagedeng | Coef. Std. Err. t P>|t| [95% Conf. Interval]

----------------+----------------------------------------------------------------

femco2 | .1049038 .0203204 5.16 0.000 .0650737 .144734

femco3 | -.0074291 .0206413 -0.36 0.719 -.0478883 .0330301

femco4 | -.030335 .0242055 -1.25 0.210 -.0777802 .0171103

femco5 | -.0190605 .0198202 -0.96 0.336 -.0579102 .0197892

femco6 | .0012445 .0177746 0.07 0.944 -.0335955 .0360845

|

refyr_rn |

1995 | -.0122858 .0145143 -0.85 0.397 -.0407355 .0161638

1997 | .0191904 .0155064 1.24 0.216 -.0112038 .0495847

1999 | .070135 .0154235 4.55 0.000 .0399032 .1003667

2003 | .0591993 .0155808 3.80 0.000 .0286593 .0897392

2006 | .0787274 .0153614 5.13 0.000 .0486174 .1088374

2008 | .0764794 .0150201 5.09 0.000 .0470384 .1059204

2010 | .1165704 .0147235 7.92 0.000 .0877108 .1454299

|

cit |

2 | -.0347544 .0147833 -2.35 0.019 -.0637312 -.0057777

3 | -.0576155 .0211168 -2.73 0.006 -.0990066 -.0162244

4 | .0336113 .0180292 1.86 0.062 -.0017279 .0689505

|

race |

2 | -.0505829 .0138237 -3.66 0.000 -.0776788 -.0234869

3 | -.0753765 .0149963 -5.03 0.000 -.1047709 -.0459821

4 | -.0911405 .0121071 -7.53 0.000 -.1148717 -.0674093

|

ba1field |

527250 | .0625432 .0221595 2.82 0.005 .0191083 .1059781

537230 | .0423497 .0447199 0.95 0.344 -.0453061 .1300055

537260 | .1587183 .0205557 7.72 0.000 .1184269 .1990097

547270 | -.3926194 .0228605 -17.17 0.000 -.4374284 -.3478105

547280 | .0199862 .0197685 1.01 0.312 -.0187622 .0587345

557330 | -.0415625 .0241536 -1.72 0.085 -.088906 .0057811

567350 | .1358603 .0197021 6.90 0.000 .0972421 .1744786

577220 | -.0220951 .0476366 -0.46 0.643 -.1154678 .0712776

577240 | -.1982232 .0263294 -7.53 0.000 -.2498316 -.1466148

577290 | -.1741542 .0344635 -5.05 0.000 -.2417063 -.1066021

577300 | .1143362 .0405006 2.82 0.005 .0349508 .1937215

577310 | -.0190361 .0300832 -0.63 0.527 -.0780023 .0399301

577320 | -.0280246 .0944287 -0.30 0.767 -.2131148 .1570655

577340 | -.0119408 .0334711 -0.36 0.721 -.0775478 .0536661

577360 | .1927229 .0770126 2.50 0.012 .0417702 .3436757

577370 | .107253 .0866367 1.24 0.216 -.062564 .2770699

577380 | -.0524794 .0780927 -0.67 0.502 -.2055492 .1005905

577390 | .0295368 .0552858 0.53 0.593 -.0788292 .1379027

577400 | .1278339 .0634383 2.02 0.044 .0034882 .2521795

577410 | -.0923715 .0331831 -2.78 0.005 -.1574138 -.0273291

|

parentba | -.0510193 .0076285 -6.69 0.000 -.0659719 -.0360667

missingparentba | -.3428871 .1135083 -3.02 0.003 -.5653754 -.1203989

_cons | .5929586 .0211017 28.10 0.000 .5515971 .6343201

**Probability of Remaining in Engineering 1-2 Years Post-BSE**

**Population: Working Full Time**

Source | SS df MS Number of obs = 13382

-------------+------------------------------ F( 40, 13341) = 54.09

Model | 404.212561 40 10.105314 Prob > F = 0.0000

Residual | 2492.59709 13341 .18683735 R-squared = 0.1395

-------------+------------------------------ Adj R-squared = 0.1370

Total | 2896.80965 13381 .216486783 Root MSE = .43225

---------------------------------------------------------------------------------

engagedeng | Coef. Std. Err. t P>|t| [95% Conf. Interval]

----------------+----------------------------------------------------------------

femco1 | 0 (omitted)

femco2 | .1140194 .0209804 5.43 0.000 .0728949 .155144

femco3 | .0025959 .0213499 0.12 0.903 -.0392529 .0444447

femco4 | .0142836 .0260175 0.55 0.583 -.0367145 .0652817

femco5 | -.0097378 .0214009 -0.46 0.649 -.0516867 .0322111

femco6 | .0429689 .0194172 2.21 0.027 .0049085 .0810293

|

refyr_rn |

1995 | -.0155022 .0152645 -1.02 0.310 -.0454227 .0144183

1997 | .0109787 .016206 0.68 0.498 -.0207874 .0427447

1999 | .0552014 .0160487 3.44 0.001 .0237437 .0866592

2003 | .0793721 .0166079 4.78 0.000 .0468183 .111926

2006 | .0663416 .0161835 4.10 0.000 .0346196 .0980637

2008 | .0747364 .0157231 4.75 0.000 .0439168 .105556

2010 | .0875374 .0160445 5.46 0.000 .056088 .1189869

|

cit |

2 | -.0016783 .0158636 -0.11 0.916 -.0327731 .0294165

3 | .0105734 .0233285 0.45 0.650 -.0351537 .0563005

4 | -.0079645 .0232902 -0.34 0.732 -.0536166 .0376877

|

race |

2 | -.0294213 .014533 -2.02 0.043 -.057908 -.0009346

3 | -.0527366 .0161478 -3.27 0.001 -.0843886 -.0210847

4 | -.0800704 .0131506 -6.09 0.000 -.1058474 -.0542934

|

ba1field |

527250 | .1010901 .0237767 4.25 0.000 .0544844 .1476959

537230 | .0400313 .0456452 0.88 0.380 -.0494399 .1295024

537260 | .1850934 .0221278 8.36 0.000 .1417198 .2284669

547270 | -.4663778 .0245052 -19.03 0.000 -.5144115 -.4183441

547280 | .0175366 .0214255 0.82 0.413 -.0244603 .0595336

557330 | -.0588462 .0256573 -2.29 0.022 -.109138 -.0085543

567350 | .1489692 .0212556 7.01 0.000 .1073052 .1906332

577220 | .0090608 .0496062 0.18 0.855 -.0881745 .106296

577240 | -.1528986 .0311052 -4.92 0.000 -.2138691 -.0919281

577290 | -.1378882 .0386967 -3.56 0.000 -.2137392 -.0620372

577300 | .1834897 .0419175 4.38 0.000 .1013254 .265654

577310 | -.01108 .0320902 -0.35 0.730 -.0739813 .0518213

577320 | .1307262 .1090989 1.20 0.231 -.083123 .3445754

577340 | .0268991 .0365337 0.74 0.462 -.0447121 .0985103

577360 | .2629118 .0810344 3.24 0.001 .1040728 .4217508

577370 | .1014798 .0864737 1.17 0.241 -.0680209 .2709804

577380 | -.093294 .0844856 -1.10 0.270 -.2588978 .0723098

577390 | -.0058342 .0638905 -0.09 0.927 -.1310687 .1194003

577400 | .1463429 .0672582 2.18 0.030 .0145073 .2781785

577410 | -.0678311 .0351063 -1.93 0.053 -.1366444 .0009821

|

parentba | -.0214972 .0079617 -2.70 0.007 -.0371033 -.005891

missingparentba | .0625349 .2297098 0.27 0.785 -.3877288 .5127986

_cons | .6253056 .0228084 27.42 0.000 .5805979 .6700132

**Probability of Leaving the Labor Force: 1-2 Years Post-BSE**

Source | SS df MS Number of obs = 16857

-------------+------------------------------ F( 40, 16816) = 18.78

Model | 41.2087344 40 1.03021836 Prob > F = 0.0000

Residual | 922.559371 16816 .054861999 R-squared = 0.0428

-------------+------------------------------ Adj R-squared = 0.0405

Total | 963.768105 16856 .057176561 Root MSE = .23423

---------------------------------------------------------------------------------

olf | Coef. Std. Err. t P>|t| [95% Conf. Interval]

----------------+----------------------------------------------------------------

femco1 | 0 (omitted)

femco2 | -.0101728 .0103345 -0.98 0.325 -.0304295 .0100838

femco3 | .0036296 .0104977 0.35 0.730 -.0169469 .0242062

femco4 | .0414293 .0123103 3.37 0.001 .0172998 .0655588

femco5 | .0124571 .0100801 1.24 0.217 -.0073009 .0322151

femco6 | .0031986 .0090397 0.35 0.723 -.0145202 .0209174

|

refyr_rn |

1995 | .00092 .0073816 0.12 0.901 -.0135488 .0153888

1997 | .0078193 .0078862 0.99 0.321 -.0076385 .0232771

1999 | -.0003946 .007844 -0.05 0.960 -.0157697 .0149805

2003 | .0166087 .007924 2.10 0.036 .0010769 .0321406

2006 | .0037834 .0078124 0.48 0.628 -.0115298 .0190966

2008 | .0068004 .0076389 0.89 0.373 -.0081726 .0217734

2010 | .0184409 .007488 2.46 0.014 .0037636 .0331181

|

cit |

2 | .0119103 .0075184 1.58 0.113 -.0028266 .0266472

3 | .0321281 .0107395 2.99 0.003 .0110776 .0531787

4 | .0492272 .0091692 5.37 0.000 .0312545 .0671998

|

race |

2 | .0173705 .0070304 2.47 0.013 .0035901 .0311508

3 | .026239 .0076268 3.44 0.001 .0112897 .0411883

4 | .0330777 .0061574 5.37 0.000 .0210086 .0451468

|

ba1field |

527250 | .0230365 .0112698 2.04 0.041 .0009466 .0451265

537230 | -.041755 .0227435 -1.84 0.066 -.0863346 .0028246

537260 | -.0148892 .0104541 -1.42 0.154 -.0353804 .005602

547270 | -.0269768 .0116263 -2.32 0.020 -.0497655 -.004188

547280 | -.0150192 .0100538 -1.49 0.135 -.0347257 .0046873

557330 | -.0047643 .0122839 -0.39 0.698 -.028842 .0193135

567350 | -.0199418 .01002 -1.99 0.047 -.0395821 -.0003015

577220 | .0044303 .0242268 0.18 0.855 -.0430568 .0519174

577240 | .1634972 .0133905 12.21 0.000 .1372504 .189744

577290 | .0297255 .0175273 1.70 0.090 -.0046299 .0640809

577300 | .0023017 .0205976 0.11 0.911 -.0380718 .0426751

577310 | -.0074652 .0152996 -0.49 0.626 -.0374539 .0225236

577320 | .0732746 .0480241 1.53 0.127 -.0208578 .167407

577340 | .0448834 .0170226 2.64 0.008 .0115173 .0782495

577360 | -.0155861 .0391667 -0.40 0.691 -.092357 .0611848

577370 | -.0060963 .0440613 -0.14 0.890 -.0924611 .0802685

577380 | .0682321 .039716 1.72 0.086 -.0096155 .1460797

577390 | .0474374 .028117 1.69 0.092 -.0076749 .1025497

577400 | -.0159974 .0322632 -0.50 0.620 -.0792366 .0472418

577410 | .0082615 .0168761 0.49 0.624 -.0248174 .0413405

|

parentba | .0349207 .0038797 9.00 0.000 .0273162 .0425253

missingparentba | .0549196 .0577276 0.95 0.341 -.0582325 .1680718

_cons | .0191443 .0107318 1.78 0.074 -.0018912 .0401797

**Probability of Remaining in Engineering 3-4 Years Post-BSE**

**Population: All**

Source | SS df MS Number of obs = 14506

-------------+------------------------------ F( 41, 14464) = 46.18

Model | 400.543704 41 9.76935864 Prob > F = 0.0000

Residual | 3059.82567 14464 .211547682 R-squared = 0.1158

-------------+------------------------------ Adj R-squared = 0.1132

Total | 3460.36937 14505 .2385639 Root MSE = .45994

---------------------------------------------------------------------------------

engagedeng | Coef. Std. Err. t P>|t| [95% Conf. Interval]

----------------+----------------------------------------------------------------

femco1 | -.0326615 .0307513 -1.06 0.288 -.092938 .027615

femco2 | .071677 .0222195 3.23 0.001 .028124 .1152299

femco3 | -.0184024 .0291934 -0.63 0.528 -.0756252 .0388203

femco4 | -.0570192 .0276718 -2.06 0.039 -.1112595 -.0027788

femco5 | -.0551715 .0188062 -2.93 0.003 -.0920341 -.018309

femco6 | -.0135218 .0252257 -0.54 0.592 -.0629673 .0359237

|

refyr_rn |

1995 | .0002775 .016781 0.02 0.987 -.0326153 .0331704

1997 | -.0050552 .0165898 -0.30 0.761 -.0375733 .027463

1999 | -.0287082 .0174195 -1.65 0.099 -.0628526 .0054362

2003 | .0108678 .017431 0.62 0.533 -.0232991 .0450347

2006 | .0454301 .0166877 2.72 0.006 .01272 .0781401

2008 | .0329765 .0167317 1.97 0.049 .0001802 .0657728

2010 | .0323509 .0158752 2.04 0.042 .0012336 .0634683

|

cit |

2 | -.0186629 .0152048 -1.23 0.220 -.0484663 .0111405

3 | .0094146 .023493 0.40 0.689 -.0366347 .0554638

4 | -.0506852 .017859 -2.84 0.005 -.0856911 -.0156794

|

race |

2 | -.0601117 .0154456 -3.89 0.000 -.090387 -.0298364

3 | -.0515532 .0162632 -3.17 0.002 -.083431 -.0196753

4 | -.122122 .0134347 -9.09 0.000 -.1484557 -.0957884

|

ba1field |

527250 | .1496911 .0240884 6.21 0.000 .1024748 .1969074

537230 | .0116555 .043114 0.27 0.787 -.0728533 .0961644

537260 | .2682791 .0220245 12.18 0.000 .2251083 .31145

547270 | -.3085684 .0240516 -12.83 0.000 -.3557127 -.2614241

547280 | .0694099 .0207676 3.34 0.001 .0287027 .1101172

557330 | -.0208779 .0250854 -0.83 0.405 -.0700485 .0282926

567350 | .1988038 .0208644 9.53 0.000 .1579068 .2397008

577220 | .0190648 .0508204 0.38 0.708 -.0805497 .1186792

577240 | -.1440241 .0326043 -4.42 0.000 -.2079327 -.0801154

577290 | -.0629939 .038452 -1.64 0.101 -.1383647 .0123769

577300 | .1700091 .045712 3.72 0.000 .0804076 .2596106

577310 | .0289094 .033486 0.86 0.388 -.0367274 .0945462

577320 | -.0846935 .1236163 -0.69 0.493 -.3269972 .1576102

577340 | .0502644 .035936 1.40 0.162 -.0201746 .1207035

577360 | .2168041 .0655799 3.31 0.001 .0882592 .345349

577370 | .0927145 .1113585 0.83 0.405 -.1255625 .3109915

577380 | .1346992 .0882411 1.53 0.127 -.0382647 .3076631

577390 | .0659887 .0664207 0.99 0.320 -.0642044 .1961818

577400 | .2828494 .0653632 4.33 0.000 .1547292 .4109696

577410 | -.0608984 .0351268 -1.73 0.083 -.1297514 .0079547

|

parentba | -.0136595 .0080539 -1.70 0.090 -.0294462 .0021272

missingparentba | .043868 .1145061 0.38 0.702 -.1805786 .2683146

_cons | .5542496 .0226175 24.51 0.000 .5099164 .5985827

---------------------------------------------------------------------------------

**Probability of Remaining in Engineering 3-4 Years Post-BSE**

**Population: Working Full Time**

Source | SS df MS Number of obs = 12501

-------------+------------------------------ F( 41, 12459) = 45.50

Model | 365.141114 41 8.90588083 Prob > F = 0.0000

Residual | 2438.75478 12459 .195742418 R-squared = 0.1302

-------------+------------------------------ Adj R-squared = 0.1274

Total | 2803.8959 12500 .224311672 Root MSE = .44243

---------------------------------------------------------------------------------

engagedeng | Coef. Std. Err. t P>|t| [95% Conf. Interval]

----------------+----------------------------------------------------------------

femco1 | .0074244 .0333995 0.22 0.824 -.0580437 .0728925

femco2 | .1014184 .0232877 4.36 0.000 .0557709 .1470658

femco3 | -.0044071 .0303277 -0.15 0.884 -.063854 .0550398

femco4 | -.006563 .029463 -0.22 0.824 -.0643151 .051189

femco5 | -.0299111 .0197975 -1.51 0.131 -.0687172 .0088951

femco6 | .0434108 .0272387 1.59 0.111 -.0099812 .0968028

|

refyr_rn |

1995 | -.0140981 .0174231 -0.81 0.418 -.0482501 .020054

1997 | -.0306406 .0171773 -1.78 0.074 -.0643109 .0030296

1999 | -.0633748 .0179149 -3.54 0.000 -.0984907 -.0282589

2003 | -.0164998 .0180219 -0.92 0.360 -.0518254 .0188258

2006 | .023529 .0173705 1.35 0.176 -.0105198 .0575778

2008 | .011517 .0173842 0.66 0.508 -.0225587 .0455927

2010 | -.0066655 .0165416 -0.40 0.687 -.0390896 .0257587

|

cit |

2 | .0053619 .016334 0.33 0.743 -.0266553 .0373791

3 | -.0147646 .0248495 -0.59 0.552 -.0634735 .0339443

4 | -.0761447 .0201984 -3.77 0.000 -.1157367 -.0365526

|

race |

2 | -.0349915 .0161818 -2.16 0.031 -.0667102 -.0032727

3 | -.0393345 .0168907 -2.33 0.020 -.0724428 -.0062261

4 | -.1067133 .0145636 -7.33 0.000 -.1352603 -.0781663

|

ba1field |

527250 | .1747757 .0253477 6.90 0.000 .1250902 .2244611

537230 | -.0136632 .043742 -0.31 0.755 -.0994042 .0720778

537260 | .2461477 .0229804 10.71 0.000 .2011025 .2911928

547270 | -.3572053 .0250826 -14.24 0.000 -.4063712 -.3080394

547280 | .0544645 .0218568 2.49 0.013 .0116218 .0973072

557330 | -.04048 .0261673 -1.55 0.122 -.0917719 .0108119

567350 | .1953052 .0219228 8.91 0.000 .1523333 .2382772

577220 | -.0393249 .0522109 -0.75 0.451 -.1416663 .0630165

577240 | -.0606533 .0386368 -1.57 0.116 -.1363874 .0150808

577290 | -.0624015 .0410774 -1.52 0.129 -.1429195 .0181165

577300 | .1776281 .0479394 3.71 0.000 .0836595 .2715966

577310 | .052637 .0352678 1.49 0.136 -.0164933 .1217672

577320 | -.0146955 .1385462 -0.11 0.916 -.2862675 .2568765

577340 | .0483938 .0379576 1.27 0.202 -.026009 .1227965

577360 | .2711612 .0693398 3.91 0.000 .1352445 .4070779

577370 | .0421672 .1084968 0.39 0.698 -.1705034 .2548377

577380 | .1022091 .0875211 1.17 0.243 -.0693459 .273764

577390 | .0979433 .0710285 1.38 0.168 -.0412834 .2371701

577400 | .2749755 .0664466 4.14 0.000 .14473 .405221

577410 | -.0774367 .0364356 -2.13 0.034 -.148856 -.0060173

|

parentba | .0049637 .0082921 0.60 0.549 -.0112901 .0212174

missingparentba | .1069871 .1205846 0.89 0.375 -.1293773 .3433516

_cons | .6126897 .0237947 25.75 0.000 .5660484 .6593311

---------------------------------------------------------------------------------

**Probability of Leaving the Labor Force: 3-4 Years Post-BSE**

Source | SS df MS Number of obs = 14506

-------------+------------------------------ F( 41, 14464) = 16.61

Model | 29.9294903 41 .729987568 Prob > F = 0.0000

Residual | 635.597049 14464 .04394338 R-squared = 0.0450

-------------+------------------------------ Adj R-squared = 0.0423

Total | 665.526539 14505 .04588256 Root MSE = .20963

---------------------------------------------------------------------------------

olf | Coef. Std. Err. t P>|t| [95% Conf. Interval]

----------------+----------------------------------------------------------------

femco1 | .0249172 .0140154 1.78 0.075 -.0025549 .0523892

femco2 | -.0016509 .0101269 -0.16 0.871 -.0215009 .018199

femco3 | -.0038768 .0133054 -0.29 0.771 -.029957 .0222035

femco4 | .0229736 .0126119 1.82 0.069 -.0017473 .0476946

femco5 | .0182794 .0085712 2.13 0.033 .0014787 .0350802

femco6 | .0011694 .011497 0.10 0.919 -.0213662 .0237051

|

refyr_rn |

1995 | -.0049932 .0076482 -0.65 0.514 -.0199847 .0099983

1997 | -.0079488 .0075611 -1.05 0.293 -.0227695 .0068719

1999 | -.0192343 .0079392 -2.42 0.015 -.0347962 -.0036724

2003 | -.0300059 .0079444 -3.78 0.000 -.045578 -.0144337

2006 | -.0094539 .0076057 -1.24 0.214 -.024362 .0054543

2008 | -.0139096 .0076257 -1.82 0.068 -.028857 .0010379

2010 | -.0173578 .0072354 -2.40 0.016 -.03154 -.0031755

|

cit |

2 | .0109542 .0069298 1.58 0.114 -.0026292 .0245375

3 | .0090736 .0107073 0.85 0.397 -.0119141 .0300613

4 | .0531929 .0081395 6.54 0.000 .0372384 .0691474

|

race |

2 | .004849 .0070396 0.69 0.491 -.0089495 .0186475

3 | .0011812 .0074122 0.16 0.873 -.0133477 .0157101

4 | .0303885 .0061231 4.96 0.000 .0183865 .0423905

|

ba1field |

527250 | .0309217 .0109787 2.82 0.005 .0094021 .0524413

537230 | -.041812 .0196499 -2.13 0.033 -.0803283 -.0032957

537260 | -.0205213 .010038 -2.04 0.041 -.0401972 -.0008455

547270 | -.0200488 .0109619 -1.83 0.067 -.0415356 .001438

547280 | -.002111 .0094652 -0.22 0.824 -.020664 .016442

557330 | -.0074119 .0114331 -0.65 0.517 -.0298222 .0149984

567350 | -.0079527 .0095093 -0.84 0.403 -.0265922 .0106868

577220 | -.0005569 .0231622 -0.02 0.981 -.0459579 .044844

577240 | .1984666 .01486 13.36 0.000 .1693392 .2275941

577290 | .0250001 .0175251 1.43 0.154 -.0093514 .0593516

577300 | .0311914 .020834 1.50 0.134 -.0096459 .0720288

577310 | .0496509 .0152618 3.25 0.001 .0197359 .079566

577320 | -.0452528 .0563402 -0.80 0.422 -.1556868 .0651812

577340 | .0093658 .0163784 0.57 0.567 -.022738 .0414696

577360 | .0131989 .0298891 0.44 0.659 -.0453875 .0717854

577370 | -.032151 .0507535 -0.63 0.526 -.1316343 .0673324

577380 | -.0422314 .0402174 -1.05 0.294 -.1210625 .0365998

577390 | .0643082 .0302724 2.12 0.034 .0049705 .1236459

577400 | -.0191976 .0297904 -0.64 0.519 -.0775905 .0391953

577410 | .0264834 .0160096 1.65 0.098 -.0048975 .0578643

|

parentba | .0281423 .0036707 7.67 0.000 .0209472 .0353373

missingparentba | -.0247249 .0521881 -0.47 0.636 -.1270202 .0775703

_cons | .0289874 .0103083 2.81 0.005 .0087818 .049193

**Probability of Remaining in Engineering 7-8 Years Post-BSE**

**Population: All**

Source | SS df MS Number of obs = 11812

-------------+------------------------------ F( 40, 11771) = 35.75

Model | 319.281182 40 7.98202954 Prob > F = 0.0000

Residual | 2628.21836 11771 .223279107 R-squared = 0.1083

-------------+------------------------------ Adj R-squared = 0.1053

Total | 2947.49955 11811 .249555461 Root MSE = .47252

---------------------------------------------------------------------------------

engagedeng | Coef. Std. Err. t P>|t| [95% Conf. Interval]

----------------+----------------------------------------------------------------

femco1 | -.0573837 .020612 -2.78 0.005 -.0977867 -.0169807

femco2 | .0695723 .0395632 1.76 0.079 -.0079782 .1471227

femco3 | -.0682387 .0322971 -2.11 0.035 -.1315464 -.0049311

femco4 | -.1201215 .0220543 -5.45 0.000 -.1633515 -.0768914

femco5 | -.0389933 .0274502 -1.42 0.155 -.0928003 .0148137

femco6 | 0 (omitted)

|

refyr_rn |

1995 | .0276121 .0175917 1.57 0.117 -.0068705 .0620947

1997 | .0053346 .0175055 0.30 0.761 -.0289791 .0396483

1999 | -.0159523 .0192059 -0.83 0.406 -.0535989 .0216944

2003 | -.0316976 .0171421 -1.85 0.064 -.0652989 .0019037

2006 | -.0176929 .0180302 -0.98 0.326 -.0530351 .0176493

2008 | .0972181 .0186616 5.21 0.000 .0606382 .133798

2010 | -.0434717 .0177684 -2.45 0.014 -.0783007 -.0086428

|

cit |

2 | -.035475 .0174746 -2.03 0.042 -.069728 -.0012219

3 | -.0516622 .0204681 -2.52 0.012 -.0917831 -.0115412

4 | -.1122813 .0188068 -5.97 0.000 -.1491458 -.0754169

|

race |

2 | .0046923 .0194967 0.24 0.810 -.0335245 .042909

3 | -.062955 .0204654 -3.08 0.002 -.1030707 -.0228394

4 | -.0706373 .016021 -4.41 0.000 -.1020411 -.0392335

|

ba1field |

527250 | .1672542 .0270321 6.19 0.000 .1142668 .2202415

537230 | -.1146193 .0428057 -2.68 0.007 -.1985256 -.030713

537260 | .2900364 .0245858 11.80 0.000 .2418443 .3382286

547270 | -.2107064 .0269725 -7.81 0.000 -.263577 -.1578359

547280 | .1282127 .0230102 5.57 0.000 .0831088 .1733165

557330 | .0450285 .02826 1.59 0.111 -.0103658 .1004229

567350 | .2595065 .0234426 11.07 0.000 .2135551 .3054579

577220 | .06923 .0508645 1.36 0.174 -.0304728 .1689327

577240 | -.0186266 .0437736 -0.43 0.670 -.1044301 .0671769

577290 | .0230012 .0453227 0.51 0.612 -.0658389 .1118412

577300 | .1808338 .048841 3.70 0.000 .0850974 .2765703

577310 | -.0253178 .0421566 -0.60 0.548 -.1079516 .0573161

577320 | -.162662 .0896006 -1.82 0.069 -.338294 .01297

577340 | .1772388 .0411274 4.31 0.000 .0966223 .2578554

577360 | .2981508 .0782827 3.81 0.000 .1447039 .4515978

577370 | .4452024 .083723 5.32 0.000 .2810915 .6093133

577380 | .1435341 .0654626 2.19 0.028 .0152165 .2718516

577390 | .0160177 .0681167 0.24 0.814 -.1175023 .1495376

577400 | .1512183 .0637194 2.37 0.018 .0263177 .2761189

577410 | -.0181017 .0392564 -0.46 0.645 -.0950507 .0588473

|

parentba | -.0129928 .0091102 -1.43 0.154 -.0308504 .0048648

missingparentba | -.0269558 .0930867 -0.29 0.772 -.2094212 .1555096

_cons | .4436758 .0245041 18.11 0.000 .3956437 .4917078

---------------------------------------------------------------------------------

**Probability of Remaining in Engineering 7-8 Years Post-BSE**

**Population: Working Full Time**

Source | SS df MS Number of obs = 10585

-------------+------------------------------ F( 40, 10544) = 34.96

Model | 305.969296 40 7.6492324 Prob > F = 0.0000

Residual | 2307.27356 10544 .218823365 R-squared = 0.1171

-------------+------------------------------ Adj R-squared = 0.1137

Total | 2613.24285 10584 .246905031 Root MSE = .46779

---------------------------------------------------------------------------------

engagedeng | Coef. Std. Err. t P>|t| [95% Conf. Interval]

----------------+----------------------------------------------------------------

femco1 | .0140553 .0230519 0.61 0.542 -.0311308 .0592413

femco2 | .1212539 .0429199 2.83 0.005 .0371229 .205385

femco3 | .0225892 .0368595 0.61 0.540 -.0496623 .0948407

femco4 | -.0925709 .0241253 -3.84 0.000 -.1398611 -.0452806

femco5 | -.0035262 .0298932 -0.12 0.906 -.0621225 .0550702

femco6 | 0 (omitted)

|

refyr_rn |

1995 | .0332869 .0183428 1.81 0.070 -.0026683 .0692422

1997 | .006238 .0182566 0.34 0.733 -.0295483 .0420243

1999 | -.0153961 .0197721 -0.78 0.436 -.054153 .0233609

2003 | -.0135029 .0178211 -0.76 0.449 -.0484356 .0214299

2006 | -.0054916 .018852 -0.29 0.771 -.042445 .0314618

2008 | .1075993 .0193869 5.55 0.000 .0695972 .1456013

2010 | -.0547291 .0184994 -2.96 0.003 -.0909915 -.0184668

|

cit |

2 | -.0105739 .0186502 -0.57 0.571 -.0471319 .0259842

3 | -.0585442 .0218597 -2.68 0.007 -.1013933 -.015695

4 | -.1038336 .0207219 -5.01 0.000 -.1444524 -.0632148

|

race |

2 | -.0146732 .0204808 -0.72 0.474 -.0548195 .025473

3 | -.0548724 .0216081 -2.54 0.011 -.0972284 -.0125164

4 | -.0754006 .0172181 -4.38 0.000 -.1091512 -.0416499

|

ba1field |

527250 | .1730554 .0282437 6.13 0.000 .1176924 .2284184

537230 | -.1233048 .0449777 -2.74 0.006 -.2114696 -.03514

537260 | .29936 .0255859 11.70 0.000 .2492068 .3495132

547270 | -.2344443 .0279428 -8.39 0.000 -.2892175 -.1796711

547280 | .1303224 .023938 5.44 0.000 .0833993 .1772455

557330 | .0378318 .029779 1.27 0.204 -.0205406 .0962043

567350 | .2639493 .0243494 10.84 0.000 .2162199 .3116788

577220 | .0753006 .052892 1.42 0.155 -.0283777 .1789789

577240 | -.0416236 .0476976 -0.87 0.383 -.13512 .0518728

577290 | .0341224 .0476967 0.72 0.474 -.0593723 .127617

577300 | .1624689 .0511497 3.18 0.001 .0622058 .262732

577310 | -.019397 .0441224 -0.44 0.660 -.1058852 .0670912

577320 | -.1577628 .0989214 -1.59 0.111 -.3516675 .0361418

577340 | .1885572 .0440067 4.28 0.000 .1022958 .2748186

577360 | .3399621 .0825641 4.12 0.000 .1781208 .5018034

577370 | .4591563 .0875687 5.24 0.000 .2875052 .6308075

577380 | .1800172 .0699947 2.57 0.010 .0428145 .31722

577390 | -.0128177 .0730002 -0.18 0.861 -.1559119 .1302764

577400 | .1929325 .0672693 2.87 0.004 .061072 .3247929

577410 | -.0266574 .0410821 -0.65 0.516 -.107186 .0538712

|

parentba | -.006638 .009528 -0.70 0.486 -.0253147 .0120388

missingparentba | .0293336 .1026938 0.29 0.775 -.1719656 .2306329

_cons | .4549021 .0254833 17.85 0.000 .4049499 .5048543

---------------------------------------------------------------------------------

**Probability of Leaving the Labor Force: 7-8 Years Post-BSE**

Source | SS df MS Number of obs = 11812

-------------+------------------------------ F( 40, 11771) = 15.28

Model | 18.3064708 40 .457661769 Prob > F = 0.0000

Residual | 352.596379 11771 .029954666 R-squared = 0.0494

-------------+------------------------------ Adj R-squared = 0.0461

Total | 370.90285 11811 .031403171 Root MSE = .17307

---------------------------------------------------------------------------------

olf | Coef. Std. Err. t P>|t| [95% Conf. Interval]

----------------+----------------------------------------------------------------

femco1 | .1057222 .0075497 14.00 0.000 .0909236 .1205209

femco2 | .0781185 .014491 5.39 0.000 .0497137 .1065233

femco3 | .0987476 .0118296 8.35 0.000 .0755595 .1219356

femco4 | .069528 .008078 8.61 0.000 .0536939 .0853621

femco5 | .0567983 .0100543 5.65 0.000 .0370901 .0765065

femco6 | 0 (omitted)

|

refyr_rn |

1995 | -.0083446 .0064434 -1.30 0.195 -.0209747 .0042856

1997 | .0091673 .0064118 1.43 0.153 -.003401 .0217356

1999 | .0009943 .0070346 0.14 0.888 -.0127947 .0147834

2003 | -.0000991 .0062787 -0.02 0.987 -.0124064 .0122083

2006 | -.0015129 .006604 -0.23 0.819 -.0144578 .0114321

2008 | -.0071467 .0068353 -1.05 0.296 -.020545 .0062516

2010 | -.0055632 .0065081 -0.85 0.393 -.0183202 .0071938

|

cit |

2 | .0088443 .0064005 1.38 0.167 -.0037018 .0213903

3 | .0125566 .007497 1.67 0.094 -.0021388 .0272519

4 | .0528838 .0068885 7.68 0.000 .0393813 .0663864

|

race |

2 | .0062992 .0071412 0.88 0.378 -.0076987 .020297

3 | -.018274 .007496 -2.44 0.015 -.0329674 -.0035806

4 | -.0022534 .0058681 -0.38 0.701 -.0137558 .0092491

|

ba1field |

527250 | .0074536 .0099012 0.75 0.452 -.0119544 .0268616

537230 | .0120469 .0156787 0.77 0.442 -.0186859 .0427797

537260 | .0079651 .0090052 0.88 0.376 -.0096865 .0256167

547270 | -.0118341 .0098794 -1.20 0.231 -.0311993 .0075311

547280 | .0072654 .0084281 0.86 0.389 -.0092551 .0237858

557330 | .0207272 .010351 2.00 0.045 .0004376 .0410168

567350 | -.001259 .0085865 -0.15 0.883 -.0180899 .0155719

577220 | .0037046 .0186304 0.20 0.842 -.0328141 .0402233

577240 | .0152876 .0160332 0.95 0.340 -.0161401 .0467154

577290 | .0612774 .0166006 3.69 0.000 .0287374 .0938173

577300 | -.0222156 .0178893 -1.24 0.214 -.0572816 .0128504

577310 | .020806 .0154409 1.35 0.178 -.0094608 .0510728

577320 | .0090491 .0328185 0.28 0.783 -.0552806 .0733789

577340 | .0404084 .015064 2.68 0.007 .0108806 .0699363

577360 | .0384212 .028673 1.34 0.180 -.0177827 .0946251

577370 | -.0036749 .0306657 -0.12 0.905 -.0637848 .0564349

577380 | -.0240539 .0239774 -1.00 0.316 -.0710535 .0229457

577390 | .045338 .0249495 1.82 0.069 -.0035671 .0942431

577400 | .0311656 .0233389 1.34 0.182 -.0145825 .0769136

577410 | .0143535 .0143787 1.00 0.318 -.013831 .0425381

|

parentba | .0076244 .0033369 2.28 0.022 .0010836 .0141652

missingparentba | .0712195 .0340954 2.09 0.037 .0043869 .1380522

_cons | .0023956 .0089752 0.27 0.790 -.0151974 .0199886

**Probability of Remaining in Engineering 9-16 Years Post-BSE if still in Engineering 7-9 Years Post-BSE**

**Population: All**

Source | SS df MS Number of obs = 884

-------------+------------------------------ F( 35, 848) = 2.78

Model | 19.1178042 35 .546222978 Prob > F = 0.0000

Residual | 166.632528 848 .196500623 R-squared = 0.1029

-------------+------------------------------ Adj R-squared = 0.0659

Total | 185.750332 883 .210362777 Root MSE = .44328

---------------------------------------------------------------------------------

engagedeng | Coef. Std. Err. t P>|t| [95% Conf. Interval]

----------------+----------------------------------------------------------------

fem84 | -.1313154 .065411 -2.01 0.045 -.2597019 -.0029289

fem85_94 | -.0623422 .0978392 -0.64 0.524 -.2543775 .1296932

fem95 | .3289248 .1287475 2.55 0.011 .0762236 .581626

|

refyr_rn |

2003 | -.0918941 .104882 -0.88 0.381 -.2977528 .1139646

2006 | -.0622424 .0938883 -0.66 0.508 -.2465231 .1220384

2008 | -.1068094 .1714963 -0.62 0.534 -.4434164 .2297976

2010 | -.1370065 .0338901 -4.04 0.000 -.2035249 -.0704882

|

cit |

2 | -.025229 .0600515 -0.42 0.675 -.1430961 .092638

3 | .0626681 .078349 0.80 0.424 -.0911125 .2164487

4 | -.3797655 .1760472 -2.16 0.031 -.7253049 -.0342261

|

race |

2 | -.0240737 .0738684 -0.33 0.745 -.1690602 .1209127

3 | .0578114 .083964 0.69 0.491 -.1069902 .222613

4 | -.0930608 .0631293 -1.47 0.141 -.2169689 .0308473

|

ba1field |

527250 | .0660995 .0913176 0.72 0.469 -.1131356 .2453345

537230 | -.113615 .1430003 -0.79 0.427 -.3942911 .1670611

537260 | .1235342 .0860948 1.43 0.152 -.0454497 .2925181

547270 | .2347098 .1144477 2.05 0.041 .0100758 .4593437

547280 | .0471207 .0834083 0.56 0.572 -.1165903 .2108316

557330 | -.1488403 .1010773 -1.47 0.141 -.3472313 .0495508

567350 | .0942182 .0833959 1.13 0.259 -.0694684 .2579048

577220 | -.0179845 .2166264 -0.08 0.934 -.4431713 .4072022

577240 | .3543787 .2950859 1.20 0.230 -.2248057 .9335631

577290 | -.1776817 .1528607 -1.16 0.245 -.4777115 .1223481

577300 | -.3156771 .1531435 -2.06 0.040 -.6162618 -.0150924

577310 | .2278521 .2931026 0.78 0.437 -.3474395 .8031437

577320 | -.8515331 .2807203 -3.03 0.002 -1.402521 -.300545

577340 | .17355 .1551843 1.12 0.264 -.1310404 .4781404

577360 | .2549303 .1832574 1.39 0.165 -.104761 .6146216

577370 | .3064374 .2544716 1.20 0.229 -.1930306 .8059054

577380 | .278043 .3575292 0.78 0.437 -.4237029 .9797889

577390 | .3261812 .1967969 1.66 0.098 -.0600849 .7124473

577400 | .339021 .152413 2.22 0.026 .0398699 .638172

577410 | -.0586196 .1299941 -0.45 0.652 -.3137675 .1965284

|

parentba | .0069667 .0311338 0.22 0.823 -.0541416 .0680751

missingparentba | .486422 .2770848 1.76 0.080 -.0574305 1.030274

_cons | .7149903 .0817289 8.75 0.000 .5545757 .8754048

**Probability of Remaining in Engineering 9-16 Years Post-BSE if still in Engineering 7-9 Years Post-BSE**

**Population: Working Full Time**

Source | SS df MS Number of obs = 848

-------------+------------------------------ F( 35, 812) = 2.58

Model | 17.1185519 35 .489101483 Prob > F = 0.0000

Residual | 153.845924 812 .189465423 R-squared = 0.1001

-------------+------------------------------ Adj R-squared = 0.0613

Total | 170.964475 847 .201847078 Root MSE = .43528

---------------------------------------------------------------------------------

engagedeng | Coef. Std. Err. t P>|t| [95% Conf. Interval]

----------------+----------------------------------------------------------------

fem84 | .0310066 .0730628 0.42 0.671 -.1124075 .1744208

fem85_94 | -.0058225 .0997174 -0.06 0.953 -.2015568 .1899119

fem95 | .2708374 .1323509 2.05 0.041 .0110472 .5306275

|

refyr_rn |

2003 | -.0923192 .1026018 -0.90 0.369 -.2937153 .1090769

2006 | -.0592213 .0921657 -0.64 0.521 -.2401324 .1216898

2008 | -.0838404 .1695145 -0.49 0.621 -.4165786 .2488978

2010 | -.1342274 .0337128 -3.98 0.000 -.200402 -.0680529

|

cit |

2 | -.0356974 .0595168 -0.60 0.549 -.1525224 .0811275

3 | .0529651 .0767474 0.69 0.490 -.0976817 .2036119

4 | -.3387379 .2383167 -1.42 0.156 -.8065274 .1290516

|

race |

2 | -.0081714 .0770552 -0.11 0.916 -.1594223 .1430795

3 | .076621 .083718 0.92 0.360 -.0877081 .2409501

4 | -.115328 .0629302 -1.83 0.067 -.2388531 .008197

|

ba1field |

527250 | -.1089926 .0984371 -1.11 0.269 -.3022139 .0842286

537230 | -.3967544 .1579206 -2.51 0.012 -.7067351 -.0867737

537260 | -.0504309 .0940456 -0.54 0.592 -.235032 .1341702

547270 | .1058108 .1203409 0.88 0.380 -.1304051 .3420267

547280 | -.1381796 .0921022 -1.50 0.134 -.3189661 .042607

557330 | -.3282832 .1076262 -3.05 0.002 -.5395415 -.1170248

567350 | -.0741105 .091653 -0.81 0.419 -.2540153 .1057942

577220 | -.1978901 .2149798 -0.92 0.358 -.6198716 .2240915

577240 | .0848056 .2904534 0.29 0.770 -.4853224 .6549337

577290 | -.3733284 .1544716 -2.42 0.016 -.6765392 -.0701176

577300 | -.2785053 .1889843 -1.47 0.141 -.6494607 .0924501

577310 | .058814 .2879521 0.20 0.838 -.5064043 .6240323

577320 | -1.011877 .2779926 -3.64 0.000 -1.557546 -.4662082

577340 | .023248 .1579579 0.15 0.883 -.2868059 .333302

577360 | .084089 .1832728 0.46 0.646 -.2756553 .4438332

577370 | .124513 .2509599 0.50 0.620 -.3680937 .6171197

577380 | .1189105 .3501055 0.34 0.734 -.5683081 .806129

577390 | .1166933 .1967367 0.59 0.553 -.269479 .5028657

577400 | .0843414 .1547266 0.55 0.586 -.2193699 .3880527

577410 | -.2434143 .1332567 -1.83 0.068 -.5049825 .018154

|

parentba | -.0274182 .0314069 -0.87 0.383 -.0890665 .03423

missingparentba | .4745355 .2698359 1.76 0.079 -.0551226 1.004194

_cons | .9085078 .0908445 10.00 0.000 .7301901 1.086825

---------------------------------------------------------------------------------

**Probability of Leaving the Labor Force 9-16 Years Post-BSE if still in Engineering 7-9 Years Post-BSE**

Source | SS df MS Number of obs = 884

-------------+------------------------------ F( 35, 848) = 9.94

Model | 7.59141405 35 .216897544 Prob > F = 0.0000

Residual | 18.5061015 848 .021823233 R-squared = 0.2909

-------------+------------------------------ Adj R-squared = 0.2616

Total | 26.0975155 883 .02955551 Root MSE = .14773

---------------------------------------------------------------------------------

olf | Coef. Std. Err. t P>|t| [95% Conf. Interval]

----------------+----------------------------------------------------------------

fem84 | .1832813 .0217986 8.41 0.000 .1404958 .2260668

fem85_94 | .052089 .0326055 1.60 0.111 -.0119079 .1160858

fem95 | -.0503511 .0429058 -1.17 0.241 -.1345652 .033863

|

refyr_rn |

2003 | -.0079351 .0349525 -0.23 0.820 -.0765387 .0606685

2006 | .0057457 .0312888 0.18 0.854 -.055667 .0671583

2008 | .0471605 .0571521 0.83 0.410 -.0650157 .1593366

2010 | .0109866 .0112941 0.97 0.331 -.011181 .0331542

|

cit |

2 | -.0233233 .0200125 -1.17 0.244 -.0626032 .0159565

3 | -.0198066 .0261102 -0.76 0.448 -.0710549 .0314416

4 | .4723169 .0586687 8.05 0.000 .3571639 .5874698

|

race |

2 | .0378503 .0246171 1.54 0.125 -.0104673 .0861678

3 | .0087238 .0279815 0.31 0.755 -.0461972 .0636449

4 | -.0216696 .0210382 -1.03 0.303 -.0629627 .0196235

|

ba1field |

527250 | -.230487 .0304321 -7.57 0.000 -.2902181 -.1707559

537230 | -.2264077 .0476557 -4.75 0.000 -.3199446 -.1328708

537260 | -.2135986 .0286916 -7.44 0.000 -.2699134 -.1572837

547270 | -.1672555 .0381403 -4.39 0.000 -.2421161 -.092395

547280 | -.2147945 .0277963 -7.73 0.000 -.2693521 -.1602369

557330 | -.189787 .0336846 -5.63 0.000 -.2559019 -.1236721

567350 | -.22022 .0277922 -7.92 0.000 -.2747695 -.1656705

577220 | -.2226908 .072192 -3.08 0.002 -.3643867 -.0809949

577240 | -.3218953 .0983391 -3.27 0.001 -.5149118 -.1288788

577290 | -.2385538 .0509417 -4.68 0.000 -.3385404 -.1385671

577300 | .1449201 .0510359 2.84 0.005 .0447486 .2450917

577310 | -.2010618 .0976781 -2.06 0.040 -.392781 -.0093426

577320 | -.2070618 .0935516 -2.21 0.027 -.3906817 -.0234418

577340 | -.1852781 .0517161 -3.58 0.000 -.2867845 -.0837716

577360 | -.2089556 .0610716 -3.42 0.001 -.3288248 -.0890864

577370 | -.2213191 .0848041 -2.61 0.009 -.3877696 -.0548685

577380 | -.1943373 .1191486 -1.63 0.103 -.4281981 .0395235

577390 | -.2559087 .0655837 -3.90 0.000 -.3846341 -.1271833

577400 | -.3067802 .0507925 -6.04 0.000 -.4064739 -.2070864

577410 | -.2286064 .0433213 -5.28 0.000 -.3136359 -.1435769

|

parentba | -.0446496 .0103755 -4.30 0.000 -.0650143 -.0242849

missingparentba | -.0350225 .0923401 -0.38 0.705 -.2162645 .1462194

_cons | .2389869 .0272366 8.77 0.000 .1855279 .292446

**Full Regression Results – Table A1**

**Probability of Remaining in Engineering 1-2 Years Post-BSE**

**Population: All**

Source | SS df MS Number of obs = 16857

-------------+------------------------------ F( 51, 16805) = 40.48

Model | 437.839612 51 8.58509044 Prob > F = 0.0000

Residual | 3564.21803 16805 .212092712 R-squared = 0.1094

-------------+------------------------------ Adj R-squared = 0.1067

Total | 4002.05764 16856 .237426296 Root MSE = .46054

---------------------------------------------------------------------------------

engagedeng | Coef. Std. Err. t P>|t| [95% Conf. Interval]

----------------+----------------------------------------------------------------

cfemXb1y_1_1991 | .1413426 .0375034 3.77 0.000 .0678319 .2148533

cfemXb1y_1_1992 | .1046047 .0449916 2.32 0.020 .0164164 .1927931

cfemXb1y_1_1993 | .1117116 .0338266 3.30 0.001 .0454078 .1780154

cfemXb1y_1_1994 | .0448463 .0423401 1.06 0.290 -.0381447 .1278373

cfemXb1y_1_1995 | .00898 .0338137 0.27 0.791 -.0572985 .0752585

cfemXb1y_1_1996 | -.0668539 .0398572 -1.68 0.093 -.1449782 .0112704

cfemXb1y_1_1997 | .011007 .0318494 0.35 0.730 -.0514212 .0734352

cfemXb1y_1_1998 | -.0443441 .0390332 -1.14 0.256 -.1208533 .032165

cfemXb1y_1_1999 | 0 (omitted)

cfemXb1y_1_2000 | 0 (omitted)

cfemXb1y_1_2001 | -.0228213 .0309901 -0.74 0.461 -.0835651 .0379225

cfemXb1y_1_2002 | -.0458563 .0371418 -1.23 0.217 -.1186582 .0269456

cfemXb1y_1_2003 | 0 (omitted)

cfemXb1y_1_2004 | .0173591 .0294827 0.59 0.556 -.0404302 .0751484

cfemXb1y_1_2005 | -.040626 .0328766 -1.24 0.217 -.1050676 .0238156

cfemXb1y_1_2006 | .04166 .0319423 1.30 0.192 -.0209503 .1042703

cfemXb1y_1_2007 | -.0136206 .0371706 -0.37 0.714 -.0864789 .0592376

cfemXb1y_1_2008 | .0020696 .0304369 0.07 0.946 -.05759 .0617292

cfemXb1y_1_2009 | -.0408432 .0364921 -1.12 0.263 -.1123715 .0306851

|

refyr_rn |

1995 | -.0062485 .0157368 -0.40 0.691 -.0370943 .0245973

1997 | .0248877 .016138 1.54 0.123 -.0067445 .0565199

1999 | .0719472 .0162593 4.43 0.000 .0400774 .1038171

2003 | .0635885 .0164168 3.87 0.000 .0314098 .0957673

2006 | .0792422 .0159105 4.98 0.000 .048056 .1104284

2008 | .0763892 .0156864 4.87 0.000 .0456421 .1071362

2010 | .1225084 .0153314 7.99 0.000 .0924572 .1525595

|

cit |

2 | -.0348727 .0147878 -2.36 0.018 -.0638584 -.0058871

3 | -.056982 .0211265 -2.70 0.007 -.0983921 -.0155719

4 | .0335185 .0180497 1.86 0.063 -.0018608 .0688978

|

race |

2 | -.0510269 .0138247 -3.69 0.000 -.0781248 -.0239289

3 | -.075551 .0150041 -5.04 0.000 -.1049605 -.0461415

4 | -.0916513 .012117 -7.56 0.000 -.115402 -.0679006

|

ba1field |

527250 | .0628494 .0221689 2.84 0.005 .019396 .1063029

537230 | .040794 .044737 0.91 0.362 -.0468953 .1284833

537260 | .1580752 .0205574 7.69 0.000 .1177805 .1983698

547270 | -.3930747 .0228657 -17.19 0.000 -.4378938 -.3482556

547280 | .0189789 .019771 0.96 0.337 -.0197742 .0577321

557330 | -.0423084 .0241547 -1.75 0.080 -.0896542 .0050375

567350 | .1354502 .0197026 6.87 0.000 .0968309 .1740694

577220 | -.0237483 .0476452 -0.50 0.618 -.1171379 .0696413

577240 | -.1978526 .0263516 -7.51 0.000 -.2495046 -.1462006

577290 | -.1744554 .0344722 -5.06 0.000 -.2420246 -.1068861

577300 | .1142125 .0405147 2.82 0.005 .0347995 .1936255

577310 | -.018966 .0300927 -0.63 0.529 -.0779509 .0400188

577320 | -.0249304 .0944684 -0.26 0.792 -.2100983 .1602376

577340 | -.0125942 .033495 -0.38 0.707 -.078248 .0530596

577360 | .1942147 .0770399 2.52 0.012 .0432085 .3452209

577370 | .1045773 .0866539 1.21 0.228 -.0652734 .274428

577380 | -.053736 .0781037 -0.69 0.491 -.2068274 .0993553

577390 | .0298362 .055299 0.54 0.590 -.0785556 .138228

577400 | .1262736 .0634449 1.99 0.047 .0019149 .2506323

577410 | -.0915467 .0332014 -2.76 0.006 -.1566249 -.0264684

|

parentba | -.0510027 .0076329 -6.68 0.000 -.0659639 -.0360414

missingparentba | -.3425993 .1136282 -3.02 0.003 -.5653226 -.1198761

_cons | .5905281 .0213226 27.69 0.000 .5487334 .6323227

---------------------------------------------------------------------------------

**Probability of Remaining in Engineering 1-2 Years Post-BSE**

**Population: Working Full Time**

Source | SS df MS Number of obs = 13382

-------------+------------------------------ F( 51, 13330) = 42.67

Model | 406.556263 51 7.97169143 Prob > F = 0.0000

Residual | 2490.25338 13330 .186815708 R-squared = 0.1403

-------------+------------------------------ Adj R-squared = 0.1371

Total | 2896.80965 13381 .216486783 Root MSE = .43222

---------------------------------------------------------------------------------

engagedeng | Coef. Std. Err. t P>|t| [95% Conf. Interval]

----------------+----------------------------------------------------------------

cfemXb1y_1_1991 | .1612006 .0388416 4.15 0.000 .0850656 .2373357

cfemXb1y_1_1992 | .094441 .0470268 2.01 0.045 .0022618 .1866201

cfemXb1y_1_1993 | .127431 .0346348 3.68 0.000 .0595419 .1953201

cfemXb1y_1_1994 | .0468384 .0434147 1.08 0.281 -.0382605 .1319373

cfemXb1y_1_1995 | .0200608 .0344462 0.58 0.560 -.0474587 .0875803

cfemXb1y_1_1996 | -.033136 .044239 -0.75 0.454 -.1198507 .0535786

cfemXb1y_1_1997 | .0051864 .0322514 0.16 0.872 -.0580308 .0684036

cfemXb1y_1_1998 | .0180203 .0422139 0.43 0.669 -.064725 .1007656

cfemXb1y_1_1999 | 0 (omitted)

cfemXb1y_1_2000 | 0 (omitted)

cfemXb1y_1_2001 | .0093132 .0331553 0.28 0.779 -.0556759 .0743022

cfemXb1y_1_2002 | -.0390025 .041079 -0.95 0.342 -.1195232 .0415182

cfemXb1y_1_2003 | 0 (omitted)

cfemXb1y_1_2004 | .0316151 .0307896 1.03 0.305 -.0287369 .0919672

cfemXb1y_1_2005 | -.0450277 .0366964 -1.23 0.220 -.116958 .0269025

cfemXb1y_1_2006 | .0821007 .0335179 2.45 0.014 .0164008 .1478007

cfemXb1y_1_2007 | .0566672 .041018 1.38 0.167 -.023734 .1370684

cfemXb1y_1_2008 | .0308214 .033809 0.91 0.362 -.035449 .0970918

cfemXb1y_1_2009 | -.0155487 .0415288 -0.37 0.708 -.096951 .0658537

|

refyr_rn |

1995 | -.009599 .0166121 -0.58 0.563 -.0421611 .022963

1997 | .0143508 .0168893 0.85 0.396 -.0187546 .0474563

1999 | .0576502 .0168947 3.41 0.001 .0245341 .0907663

2003 | .0855216 .0175014 4.89 0.000 .0512163 .1198268

2006 | .0671347 .0167561 4.01 0.000 .0342904 .099979

2008 | .072984 .0163876 4.45 0.000 .0408619 .1051061

2010 | .0959226 .0167522 5.73 0.000 .063086 .1287593

|

cit |

2 | -.0018025 .0158705 -0.11 0.910 -.0329109 .0293059

3 | .011504 .0233434 0.49 0.622 -.0342524 .0572604

4 | -.0073026 .023306 -0.31 0.754 -.0529857 .0383806

|

race |

2 | -.0298524 .0145349 -2.05 0.040 -.0583428 -.001362

3 | -.0531578 .0161539 -3.29 0.001 -.0848218 -.0214939

4 | -.0804391 .013163 -6.11 0.000 -.1062405 -.0546378

|

ba1field |

527250 | .1012986 .0237946 4.26 0.000 .0546579 .1479394

537230 | .0391059 .0456753 0.86 0.392 -.0504241 .1286359

537260 | .1844068 .02213 8.33 0.000 .1410289 .2277848

547270 | -.4674795 .0245116 -19.07 0.000 -.5155257 -.4194332

547280 | .0170385 .0214297 0.80 0.427 -.0249667 .0590438

557330 | -.0594858 .025659 -2.32 0.020 -.1097811 -.0091905

567350 | .1485837 .0212561 6.99 0.000 .1069188 .1902487

577220 | .0075707 .0496187 0.15 0.879 -.0896889 .1048304

577240 | -.1530794 .0311408 -4.92 0.000 -.2141198 -.092039

577290 | -.1390467 .0387109 -3.59 0.000 -.2149256 -.0631677

577300 | .1834603 .0419506 4.37 0.000 .1012311 .2656895

577310 | -.0120267 .0321074 -0.37 0.708 -.0749617 .0509083

577320 | .1317456 .1091349 1.21 0.227 -.0821743 .3456654

577340 | .0260681 .036552 0.71 0.476 -.045579 .0977153

577360 | .2634044 .0810564 3.25 0.001 .1045224 .4222865

577370 | .0994013 .0864801 1.15 0.250 -.070112 .2689146

577380 | -.0945356 .084501 -1.12 0.263 -.2601695 .0710983

577390 | -.0039297 .0639073 -0.06 0.951 -.1291971 .1213378

577400 | .1445287 .0672616 2.15 0.032 .0126865 .276371

577410 | -.0671178 .0351206 -1.91 0.056 -.1359592 .0017235

|

parentba | -.0212789 .0079673 -2.67 0.008 -.0368961 -.0056618

missingparentba | .05967 .2297474 0.26 0.795 -.3906676 .5100076

_cons | .6226534 .0230452 27.02 0.000 .5774816 .6678251

---------------------------------------------------------------------------------

**Probability of Leaving the Labor Force: 1-2 Years Post-BSE**

Source | SS df MS Number of obs = 16857

-------------+------------------------------ F( 51, 16805) = 15.33

Model | 42.8571274 51 .840335831 Prob > F = 0.0000

Residual | 920.910978 16805 .05479982 R-squared = 0.0445

-------------+------------------------------ Adj R-squared = 0.0416

Total | 963.768105 16856 .057176561 Root MSE = .23409

---------------------------------------------------------------------------------

olf | Coef. Std. Err. t P>|t| [95% Conf. Interval]

----------------+----------------------------------------------------------------

cfemXb1y_1_1991 | -.0148766 .0190633 -0.78 0.435 -.0522427 .0224894

cfemXb1y_1_1992 | -.0322991 .0228696 -1.41 0.158 -.0771259 .0125277

cfemXb1y_1_1993 | -.0049076 .0171943 -0.29 0.775 -.0386103 .0287951

cfemXb1y_1_1994 | .0073435 .0215218 0.34 0.733 -.0348415 .0495284

cfemXb1y_1_1995 | -.0286318 .0171878 -1.67 0.096 -.0623217 .0050581

cfemXb1y_1_1996 | .0695602 .0202597 3.43 0.001 .029849 .1092714

cfemXb1y_1_1997 | -.0064153 .0161893 -0.40 0.692 -.038148 .0253174

cfemXb1y_1_1998 | .0516029 .0198409 2.60 0.009 .0127127 .0904931

cfemXb1y_1_1999 | 0 (omitted)

cfemXb1y_1_2000 | 0 (omitted)

cfemXb1y_1_2001 | .0356872 .0157525 2.27 0.023 .0048107 .0665638

cfemXb1y_1_2002 | .0289777 .0188795 1.53 0.125 -.008028 .0659835

cfemXb1y_1_2003 | 0 (omitted)

cfemXb1y_1_2004 | -.0062072 .0149863 -0.41 0.679 -.035582 .0231675

cfemXb1y_1_2005 | .0202781 .0167114 1.21 0.225 -.0124781 .0530342

cfemXb1y_1_2006 | .0188392 .0162365 1.16 0.246 -.0129861 .0506645

cfemXb1y_1_2007 | .0402266 .0188941 2.13 0.033 .0031922 .077261

cfemXb1y_1_2008 | -.0094354 .0154713 -0.61 0.542 -.0397608 .02089

cfemXb1y_1_2009 | -.0351462 .0185492 -1.89 0.058 -.0715046 .0012122

|

refyr_rn |

1995 | -.0023552 .0079991 -0.29 0.768 -.0180344 .0133239

1997 | .0047091 .0082031 0.57 0.566 -.0113698 .020788

1999 | -.001593 .0082647 -0.19 0.847 -.0177927 .0146067

2003 | .0142721 .0083448 1.71 0.087 -.0020846 .0306288

2006 | .0037548 .0080874 0.46 0.642 -.0120974 .019607

2008 | .0007181 .0079735 0.09 0.928 -.0149108 .0163471

2010 | .0209076 .0077931 2.68 0.007 .0056324 .0361828

|

cit |

2 | .0118672 .0075168 1.58 0.114 -.0028664 .0266008

3 | .0323539 .0107388 3.01 0.003 .0113048 .0534029

4 | .0479616 .0091748 5.23 0.000 .029978 .0659451

|

race |

2 | .0173672 .0070272 2.47 0.013 .0035931 .0311413

3 | .0258851 .0076267 3.39 0.001 .010936 .0408342

4 | .0330835 .0061592 5.37 0.000 .0210109 .0451562

|

ba1field |

527250 | .0235915 .0112686 2.09 0.036 .0015037 .0456792

537230 | -.0408585 .0227402 -1.80 0.072 -.0854317 .0037146

537260 | -.0143856 .0104495 -1.38 0.169 -.0348677 .0060964

547270 | -.0267729 .0116228 -2.30 0.021 -.0495549 -.003991

547280 | -.0145826 .0100497 -1.45 0.147 -.0342811 .005116

557330 | -.0046822 .012278 -0.38 0.703 -.0287484 .0193841

567350 | -.0198386 .010015 -1.98 0.048 -.0394691 -.0002082

577220 | .0042176 .0242184 0.17 0.862 -.0432531 .0516882

577240 | .1642457 .0133947 12.26 0.000 .1379906 .1905008

577290 | .0300603 .0175225 1.72 0.086 -.0042857 .0644062

577300 | .0032588 .0205939 0.16 0.874 -.0371074 .0436251

577310 | -.0082431 .0152964 -0.54 0.590 -.0382255 .0217394

577320 | .0739256 .048019 1.54 0.124 -.0201967 .1680479

577340 | .0473634 .0170258 2.78 0.005 .013991 .0807357

577360 | -.0171111 .03916 -0.44 0.662 -.0938687 .0596466

577370 | -.0069935 .0440468 -0.16 0.874 -.0933299 .0793429

577380 | .0688548 .0397007 1.73 0.083 -.0089627 .1466724

577390 | .0492475 .0281089 1.75 0.080 -.0058488 .1043439

577400 | -.0148463 .0322495 -0.46 0.645 -.0780588 .0483662

577410 | .0072127 .0168765 0.43 0.669 -.0258671 .0402925

|

parentba | .0348519 .0038799 8.98 0.000 .027247 .0424568

missingparentba | .0566342 .0577581 0.98 0.327 -.0565777 .1698462

_cons | .0205876 .0108385 1.90 0.058 -.0006569 .0418322

---------------------------------------------------------------------------------

**Probability of Remaining in Engineering 3-4 Years Post-BSE**

**Population: All**

Source | SS df MS Number of obs = 14506

-------------+------------------------------ F( 51, 14454) = 37.51

Model | 404.478602 51 7.93095297 Prob > F = 0.0000

Residual | 3055.89077 14454 .211421805 R-squared = 0.1169

-------------+------------------------------ Adj R-squared = 0.1138

Total | 3460.36937 14505 .2385639 Root MSE = .45981

---------------------------------------------------------------------------------

engagedeng | Coef. Std. Err. t P>|t| [95% Conf. Interval]

----------------+----------------------------------------------------------------

cfemXb1y_1_1982 | 0 (omitted)

cfemXb1y_1_1983 | 0 (omitted)

cfemXb1y_1_1984 | 0 (omitted)

cfemXb1y_1_1985 | 0 (omitted)

cfemXb1y_1_1986 | 0 (omitted)

cfemXb1y_1_1987 | 0 (omitted)

cfemXb1y_1_1988 | 0 (omitted)

cfemXb1y_1_1989 | -.1103167 .0408087 -2.70 0.007 -.190307 -.0303263

cfemXb1y_1_1990 | .0541126 .0428364 1.26 0.207 -.0298522 .1380774

cfemXb1y_1_1991 | .1046496 .0425503 2.46 0.014 .0212456 .1880536

cfemXb1y_1_1992 | .0502411 .0463778 1.08 0.279 -.0406652 .1411475

cfemXb1y_1_1993 | .0818928 .0411049 1.99 0.046 .001322 .1624637

cfemXb1y_1_1994 | .0482182 .0408219 1.18 0.238 -.031798 .1282343

cfemXb1y_1_1995 | -.0021837 .0389678 -0.06 0.955 -.0785655 .0741981

cfemXb1y_1_1996 | -.0347896 .039393 -0.88 0.377 -.1120049 .0424258

cfemXb1y_1_1997 | 0 (omitted)

cfemXb1y_1_1998 | 0 (omitted)

cfemXb1y_1_1999 | -.0919416 .0367835 -2.50 0.012 -.164042 -.0198413

cfemXb1y_1_2000 | -.0207298 .0371883 -0.56 0.577 -.0936236 .052164

cfemXb1y_1_2001 | 0 (omitted)

cfemXb1y_1_2002 | -.0917796 .0350256 -2.62 0.009 -.1604344 -.0231249

cfemXb1y_1_2003 | -.0938501 .0387315 -2.42 0.015 -.1697689 -.0179314

cfemXb1y_1_2004 | -.0258256 .0326233 -0.79 0.429 -.0897715 .0381202

cfemXb1y_1_2005 | -.0173905 .0356288 -0.49 0.625 -.0872274 .0524465

cfemXb1y_1_2006 | -.0503742 .0335194 -1.50 0.133 -.1160766 .0153281

cfemXb1y_1_2007 | .0266344 .0347783 0.77 0.444 -.0415355 .0948044

cfemXb1y_1_2008 | 0 (omitted)

cfemXb1y_1_2009 | 0 (omitted)

|

refyr_rn |

1995 | -.0008254 .017127 -0.05 0.962 -.0343965 .0327456

1997 | -.0039313 .0169008 -0.23 0.816 -.0370591 .0291964

1999 | -.0287037 .0174144 -1.65 0.099 -.0628382 .0054308

2003 | .01077 .0174261 0.62 0.537 -.0233873 .0449273

2006 | .0529808 .0171546 3.09 0.002 .0193557 .0866059

2008 | .0254865 .0171932 1.48 0.138 -.0082144 .0591873

2010 | .0322235 .0158711 2.03 0.042 .0011141 .0633328

|

cit |

2 | -.0189761 .0152067 -1.25 0.212 -.0487833 .010831

3 | .0106164 .0235069 0.45 0.652 -.0354602 .0566931

4 | -.0486893 .0178906 -2.72 0.007 -.0837572 -.0136214

|

race |

2 | -.0596098 .0154467 -3.86 0.000 -.0898873 -.0293324

3 | -.0510379 .0162763 -3.14 0.002 -.0829415 -.0191342

4 | -.1225003 .0134432 -9.11 0.000 -.1488508 -.0961498

|

ba1field |

527250 | .1483938 .0241079 6.16 0.000 .1011393 .1956483

537230 | .0096288 .0431446 0.22 0.823 -.0749401 .0941976

537260 | .2680508 .0220301 12.17 0.000 .2248691 .3112326

547270 | -.3096132 .0240545 -12.87 0.000 -.3567631 -.2624632

547280 | .0687391 .02077 3.31 0.001 .0280272 .1094509

557330 | -.0224182 .0251019 -0.89 0.372 -.0716212 .0267847

567350 | .198004 .020868 9.49 0.000 .1571001 .2389079

577220 | .0171606 .0508155 0.34 0.736 -.0824442 .1167655

577240 | -.1479276 .0326445 -4.53 0.000 -.211915 -.0839403

577290 | -.0620573 .0384482 -1.61 0.107 -.1374208 .0133061

577300 | .170922 .0457152 3.74 0.000 .0813142 .2605297

577310 | .0238719 .0335327 0.71 0.477 -.0418566 .0896003

577320 | -.0874593 .1236222 -0.71 0.479 -.3297746 .154856

577340 | .048605 .0359408 1.35 0.176 -.0218436 .1190536

577360 | .2136272 .0655731 3.26 0.001 .0850955 .3421588

577370 | .0977407 .1113513 0.88 0.380 -.1205222 .3160035

577380 | .1362838 .0882272 1.54 0.122 -.0366528 .3092204

577390 | .0632848 .0664589 0.95 0.341 -.0669832 .1935527

577400 | .2790907 .065364 4.27 0.000 .1509688 .4072126

577410 | -.0592925 .0351288 -1.69 0.091 -.1281496 .0095645

|

parentba | -.0142267 .0080574 -1.77 0.077 -.0300201 .0015668

missingparentba | .0542987 .114532 0.47 0.635 -.1701986 .278796

_cons | .5552961 .0226185 24.55 0.000 .5109609 .5996313

---------------------------------------------------------------------------------

**Probability of Remaining in Engineering 3-4 Years Post-BSE**

**Population: Working Full Time**

Source | SS df MS Number of obs = 12501

-------------+------------------------------ F( 51, 12449) = 36.90

Model | 368.196131 51 7.21953198 Prob > F = 0.0000

Residual | 2435.69977 12449 .195654251 R-squared = 0.1313

-------------+------------------------------ Adj R-squared = 0.1278

Total | 2803.8959 12500 .224311672 Root MSE = .44233

---------------------------------------------------------------------------------

engagedeng | Coef. Std. Err. t P>|t| [95% Conf. Interval]

----------------+----------------------------------------------------------------

cfemXb1y_1_1989 | -.060592 .0449608 -1.35 0.178 -.1487221 .0275381

cfemXb1y_1_1990 | .0790715 .0460251 1.72 0.086 -.0111448 .1692878

cfemXb1y_1_1991 | .1337869 .0451322 2.96 0.003 .0453208 .2222529

cfemXb1y_1_1992 | .0901664 .0482927 1.87 0.062 -.0044947 .1848274

cfemXb1y_1_1993 | .1027412 .0423847 2.42 0.015 .0196606 .1858218

cfemXb1y_1_1994 | .0785926 .0435914 1.80 0.071 -.0068533 .1640385

cfemXb1y_1_1995 | -.0085793 .0400875 -0.21 0.831 -.087157 .0699984

cfemXb1y_1_1996 | -.0001322 .041559 -0.00 0.997 -.0815943 .0813298

cfemXb1y_1_1997 | 0 (omitted)

cfemXb1y_1_1998 | 0 (omitted)

cfemXb1y_1_1999 | -.0678228 .0393554 -1.72 0.085 -.1449655 .0093198

cfemXb1y_1_2000 | .0562568 .0397509 1.42 0.157 -.0216611 .1341747

cfemXb1y_1_2001 | 0 (omitted)

cfemXb1y_1_2002 | -.0535689 .03714 -1.44 0.149 -.1263691 .0192313

cfemXb1y_1_2003 | -.0802032 .0406739 -1.97 0.049 -.1599303 -.0004761

cfemXb1y_1_2004 | -.0074615 .0343221 -0.22 0.828 -.0747381 .0598151

cfemXb1y_1_2005 | .0124888 .0379488 0.33 0.742 -.0618968 .0868743

cfemXb1y_1_2006 | .0210862 .037375 0.56 0.573 -.0521747 .094347

cfemXb1y_1_2007 | .0645668 .0365347 1.77 0.077 -.0070468 .1361805

|

refyr_rn |

1995 | -.0157909 .0177639 -0.89 0.374 -.0506109 .0190292

1997 | -.0291368 .0174765 -1.67 0.096 -.0633935 .00512

1999 | -.0634858 .0179111 -3.54 0.000 -.0985943 -.0283773

2003 | -.0166708 .0180182 -0.93 0.355 -.0519893 .0186477

2006 | .0303106 .0178306 1.70 0.089 -.0046402 .0652613

2008 | .004617 .0178358 0.26 0.796 -.030344 .039578

2010 | -.0067994 .0165386 -0.41 0.681 -.0392176 .0256188

|

cit |

2 | .0045636 .0163422 0.28 0.780 -.0274696 .0365968

3 | -.0146427 .0248657 -0.59 0.556 -.0633834 .034098

4 | -.0748688 .0202274 -3.70 0.000 -.1145177 -.0352199

|

race |

2 | -.034773 .0161845 -2.15 0.032 -.0664972 -.0030488

3 | -.0378316 .0169137 -2.24 0.025 -.0709851 -.004678

4 | -.1065457 .0145819 -7.31 0.000 -.1351285 -.0779628

|

ba1field |

527250 | .1735948 .0253818 6.84 0.000 .1238426 .2233471

537230 | -.0147915 .0437606 -0.34 0.735 -.1005689 .070986

537260 | .2447542 .0229912 10.65 0.000 .199688 .2898204

547270 | -.3592876 .0250915 -14.32 0.000 -.4084708 -.3101045

547280 | .0525316 .0218654 2.40 0.016 .0096721 .0953912

557330 | -.0431126 .026192 -1.65 0.100 -.094453 .0082278

567350 | .193605 .0219351 8.83 0.000 .1506088 .2366012

577220 | -.041995 .0522174 -0.80 0.421 -.1443493 .0603592

577240 | -.0660329 .0386757 -1.71 0.088 -.1418433 .0097775

577290 | -.060196 .0410849 -1.47 0.143 -.1407288 .0203368

577300 | .1785762 .0479492 3.72 0.000 .0845882 .2725641

577310 | .0475904 .0353325 1.35 0.178 -.0216669 .1168477

577320 | -.0189502 .1385849 -0.14 0.891 -.2905981 .2526977

577340 | .0465181 .0379813 1.22 0.221 -.0279311 .1209673

577360 | .2674255 .0693458 3.86 0.000 .1314969 .403354

577370 | .0450607 .1085052 0.42 0.678 -.1676264 .2577477

577380 | .1007456 .0875108 1.15 0.250 -.070789 .2722803

577390 | .0972226 .0710214 1.37 0.171 -.0419903 .2364354

577400 | .2708203 .0664604 4.07 0.000 .1405477 .4010929

577410 | -.0779337 .0364438 -2.14 0.032 -.1493692 -.0064981

|

parentba | .0045825 .0082951 0.55 0.581 -.0116772 .0208422

missingparentba | .1065955 .1205583 0.88 0.377 -.1297174 .3429084

_cons | .6146054 .0238033 25.82 0.000 .5679473 .6612635

---------------------------------------------------------------------------------

**Probability of Leaving the Labor Force: 3-4 Years Post-BSE**

Source | SS df MS Number of obs = 14506

-------------+------------------------------ F( 51, 14454) = 13.68

Model | 30.6364906 51 .600715502 Prob > F = 0.0000

Residual | 634.890049 14454 .043924868 R-squared = 0.0460

-------------+------------------------------ Adj R-squared = 0.0427

Total | 665.526539 14505 .04588256 Root MSE = .20958

---------------------------------------------------------------------------------

olf | Coef. Std. Err. t P>|t| [95% Conf. Interval]

----------------+----------------------------------------------------------------

cfemXb1y_1_1989 | .0467869 .0186009 2.52 0.012 .0103268 .083247

cfemXb1y_1_1990 | .0003775 .0195251 0.02 0.985 -.0378942 .0386492

cfemXb1y_1_1991 | .0019888 .0193947 0.10 0.918 -.0360273 .0400048

cfemXb1y_1_1992 | -.0019836 .0211393 -0.09 0.925 -.0434193 .0394521

cfemXb1y_1_1993 | -.001642 .0187359 -0.09 0.930 -.0383667 .0350827

cfemXb1y_1_1994 | -.005461 .0186069 -0.29 0.769 -.0419329 .0310108

cfemXb1y_1_1995 | -.0288547 .0177617 -1.62 0.104 -.06367 .0059606

cfemXb1y_1_1996 | .0215596 .0179556 1.20 0.230 -.0136357 .0567548

cfemXb1y_1_1997 | 0 (omitted)

cfemXb1y_1_1998 | 0 (omitted)

cfemXb1y_1_1999 | .0059003 .0167661 0.35 0.725 -.0269635 .0387641

cfemXb1y_1_2000 | .0401461 .0169506 2.37 0.018 .0069206 .0733715

cfemXb1y_1_2001 | 0 (omitted)

cfemXb1y_1_2002 | .0139371 .0159649 0.87 0.383 -.0173562 .0452303

cfemXb1y_1_2003 | .0031461 .0176541 0.18 0.859 -.0314581 .0377504

cfemXb1y_1_2004 | .0339309 .0148699 2.28 0.023 .004784 .0630778

cfemXb1y_1_2005 | .0165426 .0162398 1.02 0.308 -.0152895 .0483747

cfemXb1y_1_2006 | .0213181 .0152784 1.40 0.163 -.0086295 .0512656

cfemXb1y_1_2007 | -.0207647 .0158522 -1.31 0.190 -.051837 .0103075

|

refyr_rn |

1995 | -.0053206 .0078066 -0.68 0.496 -.0206224 .0099813

1997 | -.0077183 .0077035 -1.00 0.316 -.0228181 .0073816

1999 | -.0193129 .0079376 -2.43 0.015 -.0348716 -.0037541

2003 | -.030019 .0079429 -3.78 0.000 -.0455881 -.0144499

2006 | -.0077232 .0078192 -0.99 0.323 -.0230498 .0076033

2008 | -.0158015 .0078368 -2.02 0.044 -.0311626 -.0004404

2010 | -.0173289 .0072341 -2.40 0.017 -.0315087 -.003149

|

cit |

2 | .0113776 .0069313 1.64 0.101 -.0022087 .0249639

3 | .0082185 .0107146 0.77 0.443 -.0127835 .0292205

4 | .0525235 .0081546 6.44 0.000 .0365393 .0685076

|

race |

2 | .004717 .0070407 0.67 0.503 -.0090837 .0185176

3 | .001734 .0074188 0.23 0.815 -.0128079 .0162758

4 | .0304201 .0061275 4.96 0.000 .0184094 .0424308

|

ba1field |

527250 | .0315845 .0109885 2.87 0.004 .0100456 .0531234

537230 | -.0418284 .0196656 -2.13 0.033 -.0803755 -.0032814

537260 | -.020954 .0100414 -2.09 0.037 -.0406365 -.0012715

547270 | -.0203419 .0109642 -1.86 0.064 -.0418331 .0011494

547280 | -.0025708 .0094671 -0.27 0.786 -.0211275 .0159859

557330 | -.0080464 .0114416 -0.70 0.482 -.0304734 .0143806

567350 | -.0082465 .0095117 -0.87 0.386 -.0268908 .0103977

577220 | -.0011807 .023162 -0.05 0.959 -.0465813 .0442198

577240 | .198138 .0148795 13.32 0.000 .1689721 .2273038

577290 | .0248026 .017525 1.42 0.157 -.0095486 .0591537

577300 | .0300367 .0208373 1.44 0.149 -.0108071 .0708804

577310 | .0504711 .0152844 3.30 0.001 .0205117 .0804306

577320 | -.0459811 .0563478 -0.82 0.415 -.1564299 .0644678

577340 | .009859 .016382 0.60 0.547 -.0222519 .0419699

577360 | .0125702 .0298886 0.42 0.674 -.0460154 .0711557

577370 | -.0336491 .0507546 -0.66 0.507 -.1331347 .0658364

577380 | -.0432776 .0402145 -1.08 0.282 -.1221032 .035548

577390 | .0640161 .0302924 2.11 0.035 .0046392 .1233931

577400 | -.0179909 .0297933 -0.60 0.546 -.0763897 .0404078

577410 | .0260133 .0160119 1.62 0.104 -.0053721 .0573988

|

parentba | .0284755 .0036726 7.75 0.000 .0212768 .0356743

missingparentba | -.02738 .0522044 -0.52 0.600 -.1297073 .0749473

_cons | .0291026 .0103097 2.82 0.005 .0088943 .0493109

---------------------------------------------------------------------------------

**Probability of Remaining in Engineering 7-8 Years Post-BSE**

**Population: All**

Source | SS df MS Number of obs = 11812

-------------+------------------------------ F( 51, 11760) = 28.78

Model | 327.021874 51 6.41219361 Prob > F = 0.0000

Residual | 2620.47767 11760 .222829734 R-squared = 0.1109

-------------+------------------------------ Adj R-squared = 0.1071

Total | 2947.49955 11811 .249555461 Root MSE = .47205

---------------------------------------------------------------------------------

engagedeng | Coef. Std. Err. t P>|t| [95% Conf. Interval]

----------------+----------------------------------------------------------------

cfemXb1y_1_1985 | -.046681 .0444842 -1.05 0.294 -.1338775 .0405155

cfemXb1y_1_1986 | -.0083624 .0485993 -0.17 0.863 -.1036252 .0869003

cfemXb1y_1_1987 | -.0976071 .0470671 -2.07 0.038 -.1898665 -.0053477

cfemXb1y_1_1988 | -.0398636 .0521068 -0.77 0.444 -.1420015 .0622743

cfemXb1y_1_1989 | -.1142727 .0463791 -2.46 0.014 -.2051835 -.023362

cfemXb1y_1_1990 | -.0203727 .0517299 -0.39 0.694 -.121772 .0810266

cfemXb1y_1_1991 | .0583218 .0525625 1.11 0.267 -.0447095 .1613531

cfemXb1y_1_1992 | .0826995 .0551481 1.50 0.134 -.0253999 .1907989

cfemXb1y_1_1993 | 0 (omitted)

cfemXb1y_1_1994 | 0 (omitted)

cfemXb1y_1_1995 | -.0623239 .0427272 -1.46 0.145 -.1460763 .0214286

cfemXb1y_1_1996 | -.0731127 .0451877 -1.62 0.106 -.1616881 .0154628

cfemXb1y_1_1997 | 0 (omitted)

cfemXb1y_1_1998 | -.0874956 .0373165 -2.34 0.019 -.1606422 -.0143491

cfemXb1y_1_1999 | -.1561079 .0445981 -3.50 0.000 -.2435276 -.0686882

cfemXb1y_1_2000 | -.1404609 .0420364 -3.34 0.001 -.2228592 -.0580625

cfemXb1y_1_2001 | -.1060656 .0426372 -2.49 0.013 -.1896416 -.0224895

cfemXb1y_1_2002 | -.1686716 .0366388 -4.60 0.000 -.2404897 -.0968534

cfemXb1y_1_2003 | .0895348 .0365092 2.45 0.014 .0179707 .161099

|

refyr_rn |

1995 | .0337584 .0190235 1.77 0.076 -.0035308 .0710476

1997 | .0114941 .0189227 0.61 0.544 -.0255976 .0485858

1999 | -.0120344 .0195435 -0.62 0.538 -.0503428 .026274

2003 | -.0280692 .0175172 -1.60 0.109 -.0624059 .0062675

2006 | -.0151662 .0189233 -0.80 0.423 -.052259 .0219266

2008 | .1015921 .0196627 5.17 0.000 .0630499 .1401344

2010 | -.0401113 .0181309 -2.21 0.027 -.0756509 -.0045717

|

cit |

2 | -.0361835 .0174834 -2.07 0.039 -.0704539 -.001913

3 | -.0543116 .0204874 -2.65 0.008 -.0944703 -.0141529

4 | -.1073532 .018825 -5.70 0.000 -.1442534 -.070453

|

race |

2 | .0033794 .019483 0.17 0.862 -.0348105 .0415694

3 | -.06655 .0204705 -3.25 0.001 -.1066756 -.0264245

4 | -.0706567 .0160231 -4.41 0.000 -.1020646 -.0392487

|

ba1field |

527250 | .1693685 .0270155 6.27 0.000 .1164137 .2223232

537230 | -.1110352 .0428027 -2.59 0.009 -.1949356 -.0271347

537260 | .2925759 .0245772 11.90 0.000 .2444006 .3407512

547270 | -.2057448 .0269715 -7.63 0.000 -.2586134 -.1528762

547280 | .1320488 .0230021 5.74 0.000 .0869609 .1771367

557330 | .0471422 .0282508 1.67 0.095 -.0082341 .1025185

567350 | .2618032 .0234322 11.17 0.000 .2158723 .3077342

577220 | .0716718 .0508252 1.41 0.159 -.027954 .1712975

577240 | -.0186094 .0438887 -0.42 0.672 -.1046386 .0674198

577290 | .028353 .0453117 0.63 0.532 -.0604654 .1171714

577300 | .1774557 .048839 3.63 0.000 .0817232 .2731883

577310 | -.019976 .0421434 -0.47 0.636 -.1025841 .0626321

577320 | -.1740436 .0895661 -1.94 0.052 -.3496079 .0015207

577340 | .1779132 .0411164 4.33 0.000 .0973183 .2585081

577360 | .3000411 .0782473 3.83 0.000 .1466634 .4534188

577370 | .4494135 .0836646 5.37 0.000 .285417 .6134101

577380 | .148129 .0654878 2.26 0.024 .019762 .276496

577390 | .0143947 .0681538 0.21 0.833 -.1191981 .1479875

577400 | .1583983 .0636911 2.49 0.013 .0335532 .2832434

577410 | -.0109207 .0392604 -0.28 0.781 -.0878776 .0660363

|

parentba | -.0143184 .0091097 -1.57 0.116 -.032175 .0035381

missingparentba | -.0227093 .0930538 -0.24 0.807 -.2051102 .1596915

_cons | .4377977 .0247809 17.67 0.000 .3892229 .4863724

---------------------------------------------------------------------------------

**Probability of Remaining in Engineering 7-8 Years Post-BSE**

**Population: Working Full Time**

Source | SS df MS Number of obs = 10585

-------------+------------------------------ F( 51, 10533) = 28.09

Model | 312.913769 51 6.13556411 Prob > F = 0.0000

Residual | 2300.32908 10533 .218392584 R-squared = 0.1197

-------------+------------------------------ Adj R-squared = 0.1155

Total | 2613.24285 10584 .246905031 Root MSE = .46732

---------------------------------------------------------------------------------

engagedeng | Coef. Std. Err. t P>|t| [95% Conf. Interval]

----------------+----------------------------------------------------------------

cfemXb1y_1_1985 | -.0096052 .0506277 -0.19 0.850 -.108845 .0896347

cfemXb1y_1_1986 | .0277825 .0553324 0.50 0.616 -.0806795 .1362444

cfemXb1y_1_1987 | -.0066725 .0521052 -0.13 0.898 -.1088085 .0954634

cfemXb1y_1_1988 | .0284709 .0573454 0.50 0.620 -.0839369 .1408787

cfemXb1y_1_1989 | -.0127914 .0532294 -0.24 0.810 -.1171312 .0915484

cfemXb1y_1_1990 | .0764669 .0574153 1.33 0.183 -.0360781 .1890118

cfemXb1y_1_1991 | .1029194 .0559372 1.84 0.066 -.0067281 .212567

cfemXb1y_1_1992 | .1443418 .0617854 2.34 0.020 .0232307 .265453

cfemXb1y_1_1993 | 0 (omitted)

cfemXb1y_1_1994 | 0 (omitted)

cfemXb1y_1_1995 | .0542869 .0507764 1.07 0.285 -.0452444 .1538183

cfemXb1y_1_1996 | -.0070611 .0501249 -0.14 0.888 -.1053153 .0911932

cfemXb1y_1_1997 | 0 (omitted)

cfemXb1y_1_1998 | -.0772467 .0403854 -1.91 0.056 -.1564097 .0019164

cfemXb1y_1_1999 | -.1273171 .0493314 -2.58 0.010 -.2240161 -.0306182

cfemXb1y_1_2000 | -.1100257 .0476267 -2.31 0.021 -.2033831 -.0166684

cfemXb1y_1_2001 | -.0627247 .0466725 -1.34 0.179 -.1542116 .0287622

cfemXb1y_1_2002 | -.1469175 .0405426 -3.62 0.000 -.2263886 -.0674464

cfemXb1y_1_2003 | .1329309 .0396361 3.35 0.001 .0552367 .2106251

|

refyr_rn |

1995 | .0331958 .0195902 1.69 0.090 -.0052048 .0715963

1997 | .0035071 .0194524 0.18 0.857 -.0346233 .0416374

1999 | -.0161274 .0200621 -0.80 0.421 -.055453 .0231982

2003 | -.0145872 .0181407 -0.80 0.421 -.0501465 .0209721

2006 | -.0057121 .0196736 -0.29 0.772 -.044276 .0328519

2008 | .1052649 .0202648 5.19 0.000 .065542 .1449878

2010 | -.056136 .0188023 -2.99 0.003 -.0929921 -.0192799

|

cit |

2 | -.0104209 .0186535 -0.56 0.576 -.0469852 .0261434

3 | -.0623111 .0218671 -2.85 0.004 -.1051747 -.0194475

4 | -.0978912 .0207434 -4.72 0.000 -.1385521 -.0572302

|

race |

2 | -.0161809 .0204681 -0.79 0.429 -.0563022 .0239405

3 | -.0570507 .0216149 -2.64 0.008 -.09942 -.0146813

4 | -.076074 .0172172 -4.42 0.000 -.109823 -.042325

|

ba1field |

527250 | .1756185 .0282351 6.22 0.000 .1202723 .2309646

537230 | -.1219068 .0450166 -2.71 0.007 -.2101479 -.0336657

537260 | .3023261 .0255767 11.82 0.000 .252191 .3524612

547270 | -.2294808 .0279442 -8.21 0.000 -.2842566 -.1747049

547280 | .1332482 .0239288 5.57 0.000 .0863433 .1801532

557330 | .0404002 .0297771 1.36 0.175 -.0179686 .0987689

567350 | .2660907 .0243403 10.93 0.000 .2183792 .3138022

577220 | .078696 .0528499 1.49 0.137 -.0248998 .1822917

577240 | -.0427457 .0478976 -0.89 0.372 -.136634 .0511426

577290 | .0366192 .0476703 0.77 0.442 -.0568236 .1300619

577300 | .1565576 .0511564 3.06 0.002 .0562814 .2568338

577310 | -.0133946 .0441118 -0.30 0.761 -.0998621 .0730728

577320 | -.1501018 .0988846 -1.52 0.129 -.3439343 .0437308

577340 | .1862604 .0439961 4.23 0.000 .1000198 .272501

577360 | .3450005 .0825424 4.18 0.000 .1832017 .5067993

577370 | .4619941 .0875094 5.28 0.000 .2904591 .6335292

577380 | .181841 .0699709 2.60 0.009 .0446849 .3189972

577390 | -.0131129 .0730815 -0.18 0.858 -.1563666 .1301408

577400 | .2011371 .0672483 2.99 0.003 .0693177 .3329565

577410 | -.0198623 .0410868 -0.48 0.629 -.1004002 .0606755

|

parentba | -.0086089 .0095325 -0.90 0.366 -.0272944 .0100767

missingparentba | .0278688 .1026229 0.27 0.786 -.1732915 .2290291

_cons | .4543631 .0257214 17.66 0.000 .4039443 .5047819

---------------------------------------------------------------------------------

**Probability of Leaving the Labor Force: 7-8 Years Post-BSE**

Source | SS df MS Number of obs = 11812

-------------+------------------------------ F( 51, 11760) = 13.21

Model | 20.0954599 51 .394028626 Prob > F = 0.0000

Residual | 350.80739 11760 .02983056 R-squared = 0.0542

-------------+------------------------------ Adj R-squared = 0.0501

Total | 370.90285 11811 .031403171 Root MSE = .17272

---------------------------------------------------------------------------------

olf | Coef. Std. Err. t P>|t| [95% Conf. Interval]

----------------+----------------------------------------------------------------

cfemXb1y_1_1982 | 0 (omitted)

cfemXb1y_1_1983 | 0 (omitted)

cfemXb1y_1_1984 | 0 (omitted)

cfemXb1y_1_1985 | .0743343 .0162761 4.57 0.000 .0424304 .1062381

cfemXb1y_1_1986 | .1100606 .0177817 6.19 0.000 .0752054 .1449158

cfemXb1y_1_1987 | .0906727 .0172211 5.27 0.000 .0569164 .124429

cfemXb1y_1_1988 | .0701381 .0190651 3.68 0.000 .0327674 .1075088

cfemXb1y_1_1989 | .1495297 .0169694 8.81 0.000 .1162669 .1827925

cfemXb1y_1_1990 | .1412342 .0189272 7.46 0.000 .1041338 .1783346

cfemXb1y_1_1991 | .0698379 .0192318 3.63 0.000 .0321404 .1075355

cfemXb1y_1_1992 | .0871671 .0201778 4.32 0.000 .0476151 .126719

cfemXb1y_1_1993 | 0 (omitted)

cfemXb1y_1_1994 | 0 (omitted)

cfemXb1y_1_1995 | .1190055 .0156332 7.61 0.000 .0883618 .1496492

cfemXb1y_1_1996 | .075244 .0165335 4.55 0.000 .0428356 .1076524

cfemXb1y_1_1997 | 0 (omitted)

cfemXb1y_1_1998 | .0566291 .0136535 4.15 0.000 .0298659 .0833922

cfemXb1y_1_1999 | .1302016 .0163178 7.98 0.000 .0982161 .1621871

cfemXb1y_1_2000 | .0561225 .0153805 3.65 0.000 .0259742 .0862708

cfemXb1y_1_2001 | .0457087 .0156003 2.93 0.003 .0151295 .0762878

cfemXb1y_1_2002 | .0961696 .0134056 7.17 0.000 .0698924 .1224467

cfemXb1y_1_2003 | .0182259 .0133582 1.36 0.172 -.0079583 .0444102

cfemXb1y_1_2004 | 0 (omitted)

cfemXb1y_1_2005 | 0 (omitted)

cfemXb1y_1_2006 | 0 (omitted)

cfemXb1y_1_2007 | 0 (omitted)

cfemXb1y_1_2008 | 0 (omitted)

cfemXb1y_1_2009 | 0 (omitted)

|

refyr_rn |

1995 | -.0066879 .0069604 -0.96 0.337 -.0203314 .0069557

1997 | .0008661 .0069235 0.13 0.900 -.0127052 .0144374

1999 | -.0011444 .0071506 -0.16 0.873 -.0151609 .0128721

2003 | -.0021693 .0064093 -0.34 0.735 -.0147325 .010394

2006 | -.0070458 .0069237 -1.02 0.309 -.0206174 .0065259

2008 | -.0050961 .0071943 -0.71 0.479 -.0191981 .0090059

2010 | -.0075075 .0066338 -1.13 0.258 -.0205109 .0054958

|

cit |

2 | .0093115 .0063969 1.46 0.146 -.0032275 .0218505

3 | .0126234 .007496 1.68 0.092 -.0020701 .0273168

4 | .0522693 .0068878 7.59 0.000 .0387681 .0657705

|

race |

2 | .0068206 .0071285 0.96 0.339 -.0071525 .0207937

3 | -.0180317 .0074898 -2.41 0.016 -.032713 -.0033504

4 | -.0029829 .0058626 -0.51 0.611 -.0144745 .0085088

|

ba1field |

527250 | .0075534 .0098845 0.76 0.445 -.0118219 .0269287

537230 | .0110323 .0156609 0.70 0.481 -.0196656 .0417302

537260 | .0074112 .0089924 0.82 0.410 -.0102154 .0250379

547270 | -.012883 .0098685 -1.31 0.192 -.0322269 .0064608

547280 | .0058116 .0084161 0.69 0.490 -.0106853 .0223086

557330 | .0201326 .0103365 1.95 0.051 -.0001288 .0403939

567350 | -.0016312 .0085735 -0.19 0.849 -.0184366 .0151742

577220 | .0033405 .0185961 0.18 0.857 -.033111 .0397921

577240 | .0148636 .0160582 0.93 0.355 -.0166132 .0463403

577290 | .0578106 .0165788 3.49 0.000 .0253133 .0903079

577300 | -.0227822 .0178694 -1.27 0.202 -.0578093 .0122449

577310 | .019739 .0154196 1.28 0.201 -.0104861 .049964

577320 | .0135298 .0327708 0.41 0.680 -.0507065 .077766

577340 | .0413754 .0150438 2.75 0.006 .011887 .0708638

577360 | .0362128 .0286295 1.26 0.206 -.0199057 .0923314

577370 | -.005795 .0306116 -0.19 0.850 -.0657988 .0542088

577380 | -.0222009 .023961 -0.93 0.354 -.0691684 .0247666

577390 | .047302 .0249364 1.90 0.058 -.0015775 .0961816

577400 | .0276524 .0233036 1.19 0.235 -.0180265 .0733313

577410 | .0107294 .0143648 0.75 0.455 -.017428 .0388868

|

parentba | .0076276 .0033331 2.29 0.022 .0010941 .014161

missingparentba | .0695324 .034047 2.04 0.041 .0027948 .1362701

_cons | .0053863 .009067 0.59 0.552 -.0123865 .023159

---------------------------------------------------------------------------------
