# Supplementary material for: Are recent cohorts of women with engineering bachelors less likely to stay in engineering?
Source: Front Psychol. 2015 Aug 19;6:1144. doi: 10.3389/fpsyg.2015.01144 (PMC4541081; doi:10.3389/fpsyg.2015.01144)
Supplement: Supplementary file 2 [file DataSheet1.DOCX]

Table A1: Gender Differences in Remaining in Engineering by BSE Year (calculated as coefficients Female X BSE Year interaction terms)

	Probability of Remaining in Engineering		Probability of Leaving the Labor Force	Probability of Remaining in Engineering		Probability of Leaving the Labor Force	Probability of Remaining in Engineering		Probability of Leaving the Labor Force
Cohort (BSE	All	Working FT	All	All	Working FT	All	All	Working FT	All
years)	Panel A. 1-2 Years post-BSE			Panel B. 3-4 Years post-BSE			Panel C. 7-8 Years post-BSE		
1985							-0.0467	-0.0096	0.0743***
							(0.0445)	(0.0506)	(0.0163)
1986							-0.0084	0.0278	0.1101***
							(0.0486)	(0.0553)	(0.0178)
1987							-0.0976**	-0.0067	0.0907***
							(0.0471)	(0.0521)	(0.0172)
1988							-0.0399	0.0285	0.0701***
							(0.0521)	(0.0573)	(0.0191)
1989				-0.1103***	-0.0606	0.0468**	-0.1143**	-0.0128	0.1495***
				(0.0408)	(0.0450)	(0.0186)	(0.0464)	(0.0532)	(0.0170)
1990				0.0541	0.0791*	0.0004	-0.0204	0.0765	0.1412***
				(0.0428)	(0.0460)	(0.0195)	(0.0517)	(0.0574)	(0.0189)
1991	0.1413***	0.1612***	-0.0149	0.1046**	0.1338***	0.0020	0.0583	0.1029*	0.0698***
	(0.0375)	(0.0388)	(0.0191)	(0.0426)	(0.0451)	(0.0194)	(0.0526)	(0.0559)	(0.0192)
1992	0.1046**	0.0944**	-0.0323	0.0502	0.0902*	-0.0020	0.0827	0.1443**	0.0872***
	(0.0450)	(0.0470)	(0.0229)	(0.0464)	(0.0483)	(0.0211)	(0.0551)	(0.0618)	(0.0202)
1993	0.1117***	0.1274***	-0.0049	0.0819**	0.1027**	-0.0016			
	(0.0338)	(0.0346)	(0.0172)	(0.0411)	(0.0424)	(0.0187)			
1994	0.0448	0.0468	0.0073	0.0482	0.0786*	-0.0055			
	(0.0423)	(0.0434)	(0.0215)	(0.0408)	(0.0436)	(0.0186)			
1995	0.0090	0.0201	-0.0286*	-0.0022	-0.0086	-0.0289	-0.0623	0.0543	0.1190***
	(0.0338)	(0.0344)	(0.0172)	(0.0390)	(0.0401)	(0.0178)	(0.0427)	(0.0508)	(0.0156)
1996	-0.0669*	-0.0331	0.0696***	-0.0348	-0.0001	0.0216	-0.0731	-0.0071	0.0752***
	(0.0399)	(0.0442)	(0.0203)	(0.0394)	(0.0416)	(0.0180)	(0.0452)	(0.0501)	(0.0165)
1997	0.0110	0.0052	-0.0064						
	(0.0318)	(0.0323)	(0.0162)						
1998	-0.0443	0.0180	0.0516***				-0.0875**	-0.0772*	0.0566***
	(0.0390)	(0.0422)	(0.0198)				(0.0373)	(0.0404)	(0.0137)
1999				-0.0919**	-0.0678*	0.0059	-0.1561***	-0.1273***	0.1302***
				(0.0368)	(0.0394)	(0.0168)	(0.0446)	(0.0493)	(0.0163)
2000				-0.0207	0.0563	0.0401**	-0.1405***	-0.1100**	0.0561***
				(0.0372)	(0.0398)	(0.0170)	(0.0420)	(0.0476)	(0.0154)
2001	-0.0228	0.0093	0.0357**				-0.1061**	-0.0627	0.0457***
	(0.0310)	(0.0332)	(0.0158)				(0.0426)	(0.0467)	(0.0156)
2002	-0.0459	-0.0390	0.0290	-0.0918***	-0.0536	0.0139	-0.1687***	-0.1469***	0.0962***
	(0.0371)	(0.0411)	(0.0189)	(0.0350)	(0.0371)	(0.0160)	(0.0366)	(0.0405)	(0.0134)
2003				-0.0939**	-0.0802**	0.0031	0.0895**	0.1329***	0.0182
				(0.0387)	(0.0407)	(0.0177)	(0.0365)	(0.0396)	(0.0134)
2004	0.0174	0.0316	-0.0062	-0.0258	-0.0075	0.0339**			
	(0.0295)	(0.0308)	(0.0150)	(0.0326)	(0.0343)	(0.0149)			
2005	-0.0406	-0.0450	0.0203	-0.0174	0.0125	0.0165			
	(0.0329)	(0.0367)	(0.0167)	(0.0356)	(0.0379)	(0.0162)			
2006	0.0417	0.0821**	0.0188	-0.0504	0.0211	0.0213			
	(0.0319)	(0.0335)	(0.0162)	(0.0335)	(0.0374)	(0.0153)			
2007	-0.0136	0.0567	0.0402**	0.0266	0.0646*	-0.0208			
	(0.0372)	(0.0410)	(0.0189)	(0.0348)	(0.0365)	(0.0159)			
2008	0.0021	0.0308	-0.0094						
	(0.0304)	(0.0338)	(0.0155)						
2009	-0.0408	-0.0155	-0.0351*						
	(0.0365)	(0.0415)	(0.0185)						

See Notes Table 3
